# Analysis of 23andMe antidepressant efficacy survey data: implication of circadian rhythm and neuroplasticity in bupropion response

**DOI:** 10.1038/tp.2016.171

**Published:** 2016-09-13

**Authors:** Q S Li, C Tian, G R Seabrook, W C Drevets, V A Narayan

**Affiliations:** 1 Neuroscience Therapeutic Area, Janssen Research & Development, LLC, Titusville, NJ, USA; 2 23andMe, Inc., Mountain View, CA, USA; 3 Janssen Research & Development, Menlo Park, CA, USA

## Abstract

Genetic predisposition may contribute to the differences in drug-specific, class-specific or antidepressant-wide treatment resistance. Clinical studies with the genetic data are often limited in sample sizes. Drug response obtained from self-reports may offer an alternative approach to conduct a study with much larger sample size. Using the phenotype data collected from 23andMe ‘Antidepressant Efficacy and Side Effects' survey and genotype data from 23andMe's research participants, we conducted genome-wide association study (GWAS) on subjects of European ancestry using four groups of phenotypes (a) non-treatment-resistant depression (*n*=7795) vs treatment-resistant depression (TRD, *n*=1311), (b) selective serotonin reuptake inhibitors (SSRI) responders (*n*=6348) vs non-responders (*n*=3340), (c) citalopram/escitalopram responders (*n*=2963) vs non-responders (*n*=2005), and (d) norepinephrine–dopamine reuptake inhibitor (NDRI, bupropion) responders (*n*=2675) vs non-responders (*n*=1861). Each of these subgroups was also compared with controls (*n* ~ 190 000). The most significant association was from bupropion responders vs non-responders analysis. Variant rs1908557 (*P*=2.6 × 10^−8^, OR=1.35) passed the conventional genome-wide significance threshold (*P*=5 × 10^−8^) and was located within the intron of human spliced expressed sequence tags in chromosome 4. Gene sets associated with long-term depression, circadian rhythm and vascular endothelial growth factor (VEGF) pathway were enriched in the bupropion analysis. No single-nucleotide polymorphism passed genome-wide significance threshold in other analyses. The heritability estimates for each response group compared with controls were between 0.15 and 0.25, consistent with the known heritability for major depressive disorder.

## Introduction

Despite the Psychiatric Genomic Consortium (PGC)'s collaborative efforts of major depressive disorder (MDD) meta-analysis, variants predictive of disease susceptibility remain elusive, partly due to the heterogeneous nature of the disorder. Treatment for MDD and genetic predictor of treatment response are equally challenging. There are ~30 antidepressants available for MDD patient care and response to treatment varies in time to onset of benefit, overall efficacy, and duration of effect. Genetic variability may contribute to the differences in drug-specific, class-specific or antidepressant-wide treatment non-response/resistance. Several antidepressant efficacy genome-wide association studies (GWASs) have been conducted using samples from the Munich Antidepressant Response Signature project (a naturalistic prospective study, *n*=339, genotyped using Illumina Sentrix Human-1 (109,000 loci) and HumanHap300 (317 000 loci) BeadChip),^1^ the Genome-Based Therapeutic Drugs for Depression (GENDEP) project (*n*=394 on escitalopram and *n*=312 on nortriptyline genotyped using Illumina Human610-quad chip),^2^ the Sequenced Treatment Alternatives to Relieve Depression (STAR*D) study (*n*=1491 on citalopram genotyped using the Affymetrix 500K and 5.0 Human SNP Arrays),^3^ and the Mayo Clinic Pharmacogenomic Research Network Antidepressant Medication Pharmacogenomic Study (PGRN-AMPS) study (*n*=529 on selective serotonin reuptake inhibitor (SSRI) genotyped using Illumina Human610-Quad BeadChips).^4^ The largest antidepressant efficacy GWAS meta-analysis performed using the data from three studies among subjects of European ancestry only (STAR*D *n*=980, GENDEP *n*=706, and Munich Antidepressant Response Signature including additional samples genotyped *n*=604 resulting a total sample size of 2256 across three studies) did not identify any variants passing genome-wide significance threshold with primary outcome measurements (percentage improvement on the clinician-rated depression scale and remission in each study after 12 weeks of treatment), although a secondary analysis restricted to 1354 individuals treated with either citalopram or escitalopram revealed a variant rs12054895 (*P*=2.65 × 10^−8^) in the intergenic region of chromosome 5 associated with early improvement after 2 weeks of antidepressant treatment.^5^ Drug response information obtained from self-reported questionnaires may offer an alternative approach to conduct a study with much larger sample size.

MDD is a heterogeneous disease,^6^ which partially explained why finding disease risk variants for MDD seems to be more challenging than for schizophrenia and bipolar. Disease subtypes have been defined by clinical features such as MDD with the melancholic, atypical or anxious distress specifiers.^7^ Theoretically, different disease subtypes may be defined by different underlying molecular mechanism, which in turn determines the response to different therapeutic agents. In this study, we explored different disease subtypes as defined by response status and compared the heritability and genetic correlation to reference populations (PGC1 MDD, bipolar and schizophrenia).

## Materials and methods

### Cohort

Approximately 48 000 research participants, drawn from 23andMe (Mountain View, CA, USA), provided saliva samples for genetic testing, informed consent and answered surveys (‘Antidepressant Efficacy and Side Effects' and ‘Your Profile and Health History') online between June 2013 and June 2015 according to a human subjects protocol reviewed and approved by Ethical & Independent Review Services, an AAHRPP-accredited Institutional Review Board (http://www.eandireview.com). Studies on other selected phenotypes from 23andMe were previously reported.^8, 9, 10, 11, 12, 13, 14^

### Sample genotyping and SNP data imputation

DNA extraction and genotyping were performed as described before,^9^ on saliva samples by National Genetics Institute (NGI, Los Angeles, CA, USA). Samples have been genotyped on one of four genotyping platforms. The V1 and V2 platforms were variants of the Illumina HumanHap550+ BeadChip (Illumina, San Diego, CA, USA), including about 25 000 custom single-nucleotide polymorphisms (SNPs) selected by 23andMe, with a total of about 560 000 SNPs. The V3 platform was based on the Illumina OmniExpress+ BeadChip, with custom content to improve the overlap with the V2 array, with a total of about 950 000 SNPs. The V4 platform in current use is a fully custom array, including a lower redundancy subset of V2 and V3 SNPs with additional coverage of lower-frequency-coding variation, and about 570 000 SNPs. Samples that failed to reach 98.5% call rate were reanalyzed. Participant genotype data were imputed against the September 2013 release of 1000 Genomes^15^ Phase1 reference haplotypes, phased with ShapeIt2. Prior to imputation, we excluded SNPs with Hardy–Weinberg equilibrium *P*<10^−20^, call rate <95%, or with large allele frequency discrepancies compared to European 1000 Genomes reference data. Additional details on the imputation procedure could be found in Supplementary Text S1.

### Phenotype data and phenotypic analysis groups

The ‘Antidepressant Efficacy and Side Effects' questionnaire was designed by 23andMe in collaboration with Dr Steven Hamilton of Kaiser Permanente San Francisco Medical Center and Carol Cochran Schaffner at the University of California, San Francisco. The questionnaires asked respondents in their use of antidepressants and antipsychotics in the last 5 years and the effect qualitatively (for example, ‘How well did Wellbutrin/bupropion work for you?'. The list of drugs includes SSRIs (citalopram, escitalopram, fluoxetine, paroxetine and sertraline), serotonin-norepinephrine reuptake inhibitors (duloxetine, venlafaxine and desvenlafaxine), norepinephrine–dopamine reuptake inhibitor (NDRI) (bupropion), serotonin antagonist and reuptake inhibitor trazodone, and atypical antipsychotics (quetiapine, olanzapine and aripiprazole). The antidepressant efficacy question in the survey has five possible answers ranging from a great deal (coded as 4 for later reference), a fair amount (3), somewhat (2), a little (1) to not at all (0). The ‘Your Profile and Health History' survey asked a member's medical history.

Using phenotype data collected from 23andMe surveys (‘Antidepressant Efficacy and Side Effects' and ‘Your Profile and Health History') and genotype data from 23andMe's research participants, we performed genome-wide association analyses on four groups of phenotypes (a) non-treatment-resistant depression (*n*=7795) vs treatment-resistant depression (TRD) (*n*=1311), (b) SSRI responders (*n*=6348) vs non-responders (*n*=3340), (c) citalopram/escitalopram responders (*n*=2963) vs non-responders (*n*=2005) and (d) bupropion responders (*n*=2675) vs non-responders (*n*=1861). All subjects included in the analyses self-reported taking antidepressants for depression indication and were of European ancestry. TRD were defined as subjects who reported efficacy ⩽1 to at least 2 antidepressants and never reported efficacy ⩾3 to any antidepressant, whereas non-TRD were defined as subjects who reported efficacy ⩾3 to at least one antidepressants and never reported efficacy ⩽1 for any antidepressant. SSRI non-responders were defined as subjects who reported efficacy ⩽1 to at least one SSRI and never reported efficacy ⩾3 to any SSRI antidepressant, whereas SSRI responders were defined as subjects who reported efficacy ⩾3 to at least one SSRI and never reported efficacy ⩽1 to any SSRI antidepressant. Citalopram/escitalopram non-responders were subjects who reported efficacy ⩽1 to either citalopram or escitalopram and never reported efficacy ⩾3 to either citalopram or escitalopram, whereas citalopram/escitalopram responders were subjects who reported efficacy ⩾3 to either citalopram or escitalopram and never reported efficacy ⩽1 to either citalopram or escitalopram. Likewise, bupropion non-responders were subjects who reported efficacy ⩽1 to bupropion and bupropion responders were subjects who reported efficacy ⩾3 to bupropion.

For each of the four phenotype groups, the resistant/non-responder group and the non-resistant/responder group were also compared with healthy controls (*n*~190 000) self-reported to be free of any of the following conditions based on the survey data captured from the ‘Your Profile and Health History' survey: attention-deficit/hyperactivity disorder; anxiety; schizophrenia; depression; bipolar; obsessive-compulsive disorder; autism; post-traumatic stress disorder; and insomnia.

### Genome-wide association analysis

We restricted GWAS to a set of unrelated individuals who have >97% European ancestry, as determined through an analysis of local ancestry. Standard quality control on directly genotyped markers excluded (1) SNPs that were only genotyped on our ‘V1' and/or ‘V2' platforms due to small sample size, and SNPs on chrM or chrY; (2) SNPs that failed a test for parent-offspring transmission using trio data; (3) Hardy–Weinberg *P*<10^−20^ in Europeans; or (4) SNPs with call rate of <90% (5) SNPs with genotyping batch effect. For imputed markers, we excluded markers with avg.rsq<0.5 or min.rsq<0.3 in any imputation batch and markers with significant imputation batch effect. For case control comparisons, we compute association test results by logistic regression assuming additive allelic effects using custom scripts implemented by 23andMe in C^++^ programing language, which were also used to compute association test results in previous publications.^8, 9, 10, 11, 12, 13, 14^ For tests using imputed data, we use the imputed dosages rather than best-guess genotypes. We include covariates for age, gender, genotype platforms and the top five principal components to account for residual population structure. The association test *P*-value we report is computed using a two-sided likelihood ratio test, which is better behaved than a Wald test on the regression coefficient. A *P*-value threshold of 5 × 10^−8^ is considered to be genome-wide significant. No additional multiple testing correction was applied for considering multiple phenotype groups. Additional details on the method could be found in Supplementary Text S1.

### Genetic heritability and genetic correlation estimates

PGC phase 1 disease susceptibility summary association statistics for MDD, bipolar, and schizophrenia^16, 17, 18, 19^ were downloaded from PGC website (http://www.med.unc.edu/pgc/downloads) and included together with the summary statistics from this study as reference data sets for genetic heritability and genetic correlation estimates. Phenotypic variance explained by variants (both genotyped and imputed, mostly SNPs) (*h*^2^) for each of the phenotype groups and the genetic correlation between traits (*r*_g_) were estimated using association statistics as implemented in LD Score regression.^20^

### Gene set enrichment analysis

INRICH is a pathway analysis tool for genome-wide association studies, designed for detecting enriched association signals of linkage disequilibrium (LD)-independent genomic regions within biologically relevant gene sets.^21^ Reference gene sets used in the INRICH analysis include KEGG, Gene Ontology and Molecular Signature Database (v5.0). Top variants from responder vs non-responder analyses with nominal association *P*-value <0.0005, 0.0001, 0.00005, 0.00001 were separately fed into PLINK to clump the variants into LD-independent genomic intervals (*r*^2^ threshold using 0.2, 0.3 and 0.5, respectively), then LD-independent genomic regions were used for INRICH (version 1.0) analyses. No multiple testing corrections were applied for running INRICH against multiple reference gene sets or for using multiple parameters (*P*-value cutoff and LD threshold).

## Results

Sample sizes for each phenotypic group together with demographic variables such as gender and age as well as genotyping platform used and GWAS genomic control inflation factor lambda are listed in Supplementary Table S1. Overall, the prevalence rate for depressed patient was greater in females than in males, consistent with the extant epidemiological literature. Lambda scores (*λ*_1000_) between 1.002 and 1.013 revealed no departures from uniform distributions of *P*-values across ~12 million genotyped and imputed markers.

**Figure 1:**
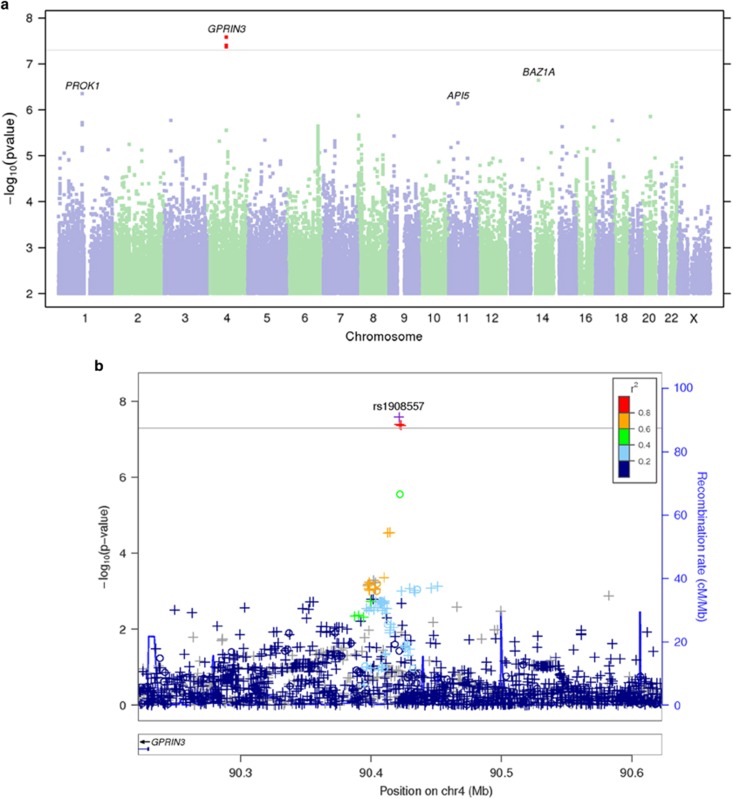
Bupropion responders vs non-responders GWAS. (**a**) Manhattan plot—the Manhattan plot depicts the distribution of association test statistics versus genomic position, with chromosomes 1 to 22, X, and Y arranged along the *x* axis. The *y* axis represents log-scaled *P*-values. Positions with *P*<5 × 10−8 (a score of about 7.3) are shown in red. Loci with smallest *P*<10−6 are labeled with the name of the nearest gene. A ‘good' Manhattan plot should show towers of single-nucleotide polymorphisms (SNPs) with small *P*-values supporting most signals that pass the genome-wide threshold. (**b**) Regional association plots—the regional association plots show association test statistics versus position in the vicinity of the strongest associations. The plots are generated with LocusZoom,^22^ using linkage disequilibrium data from the March 2012 release of 1000 Genomes data. In the plots, a ‘o' symbol indicates a genotyped SNP and a ‘+' indicates an imputed SNP. Color indicates strength of linkage disequilibrium with the index SNP. GWAS, genome-wide association study.

Out of a total of 12 genome-wide association analyses performed, only the bupropion responders vs non-responders analysis yielded a locus reaching genome-wide significance threshold (*P*=5 × 10^−8^; Figure 1a). The most significant association variant rs1908557 (*P*=2.6 × 10^−8^; OR =1.35) was located within the intron of human spliced expressed sequence tags between known genes GPRIN family member 3 (*GPRIN3*) and synuclein alpha (*SNCA*) in chromosome 4 (Figure 1b) overlapping with enhancer/promoter-associated histone mark H3K4Me1. Each copy of rs1908557-C allele was associated with higher odds of being bupropion non-responder. The frequency of C allele was relatively common (minor allele frequency=25%) in the study population. No SNP passed genome-wide significance threshold in any other GWAS analyses. Manhattan and quantile–quantile plots for all analyses (Supplementary Figure S1 and S2) as well as variants with a nominal association *P*-value in any of the 12 analyses <5 × 10^−5^ (Supplementary Dataset 1) are available online. The indexed SNPs with *P*<1 × 10^−5^ for the responders vs non-responders analyses and the responder subgroup vs healthy control group analyses are listed in Tables 1 and 2, respectively.

The heritability estimates for responders vs non-responders or non-TRD vs TRD analyses were generally unreliable with large standard errors and smaller *h*^2^ estimates (Table 3). The heritability estimates for each responder, non-responder, non-TRD, TRD group in comparison to healthy controls were between 0.14 and 0.22, consistent with the known heritability estimates for MDD (*h*^2^=0.19) estimated from PGC1 MDD samples. The genetic correlation between responder vs non-responder and non-TRD vs TRD in general are less similar than genetic correlations among responder/non-responder/non-TRD/TRD vs healthy controls, as expected.

Gene set enrichment analysis may yield signals of enriched gene sets in GWAS analysis despite the individual variants not reaching genome-wide significance. Applying INRICH^21^ enrichment analysis to the NDRI (bupropion) responders vs non-responders GWAS results implicated gene sets such as circadian rhythm, long-term depression (LTD), and vascular endothelial growth factor (VEGF) pathway being enriched among the bupropion suggestive association hits (*P*<0.0005; Table 4, Supplementary Table S2 for gene lists belonging to each enriched gene sets in Table 4, Supplementary Dataset 2 for SNP lists corresponding to gene lists from Supplementary Table S2). Additional enriched gene sets for other GWAS analyses are listed in Supplementary Table S3.

## Discussion

We identified a candidate genetic marker rs1908557 in the intergenic region between *GPRIN3* and *SNCA* for bupropion response with an association *P*-value passing genome-wide significance using a phenotype derived from survey data. This finding ultimately will require replication in clinically ascertained samples to further dissect the genetic basis of treatment response to bupropion among depression patients. The biological significance of rs1908557 is unknown except that rs1908557 is also marginally associated with two brain regions known to exhibit volumetric difference between MDD subjects and healthy controls. Specifically, patients carrying the common T allele are associated with lower hippocampal (*P*=0.016), amygdala (*P*=0.067) and nucleus accumbens volumes (*P*=0.067).^23^ In the recent ENIGMA analysis of 1728 MDD patients and 7199 controls, MDD (especially recurrent MDD and patients with earlier age of onset (AOO⩽21 years)) had significantly lower hippocampal volumes, although patients with earlier AOO also showed a trend toward smaller amygdala volumes.^24^ In our analysis the T allele was associated specifically with better bupropion response. It remains unclear whether the intergenic variant has any biological impact on the neighboring gene *SNCA*. So far the limited sample size from GTEx did not support any eQTL relationship between rs1908557 and the neighboring genes; however, the overlap of enhancer/promoter-associated histone mark H3K4Me1cannot rule out that there may be still more subtle relationship between neighbor genes and rs1908557. However, eQTL data from BRAINEAC^25^ (http://braineac.org/) suggested a subtle cis-eQTL relationship between rs1908557 and genes further away from rs1908557 such as family with sequence similarity 13 member A (*FAM13A*), multimerin 1 (*MMRN1*), HECT and RLD domain containing E3 ubiquitin protein ligase 5 (*HERC5*), and coiled-coil serine rich protein 1 (*CCSER1*, also known as *FAM190A*; see Supplementary Table S4). Additional discussion of the possible genes involved is available from Supplementary Text S2. In addition, several variants with suggestive association with bupropion response such as rs8076666 in solute carrier family 26 (anion exchanger), member 11 (*SLC26A11, P*=1.59 × 10^−6^) and rs9373491 in glutamate receptor, metabotropic 1 (*GRM1*, *P*=3.01 × 10^−6^) also showed suggestive volumetric relationships with brain volumes^23^ (Supplementary Dataset 1).

It is noteworthy that the gene set enrichment analysis of bupropion GWAS yielded the most gene sets of interest to MDD. In particular, circadian rhythm, LTD and VEGF pathway genes were enriched among the bupropion suggestive association hits (*P*<0.0005). Alteration of circadian rhythms and disturbances of sleep are common features of the major depressive syndrome. Variants from period circadian clock 3 (*PER3*, representative intronic variants rs7528457, *P*=3.81 × 10^−5^; rs12137927 *P*=6.30 × 10^−5^), RAR-related orphan receptor A *(RORA*, rs185937898, *P*=5.51 × 10^−5^), and nuclear receptor subfamily 1, group D, member 1 (*NR1D1*) were accountable for circadian rhythm gene set enrichment in the bupropion response GWAS. Acute and chronic stress, which putatively can be precipitating factors of MDD, also can affect rhythms of the circadian pacemaker. *PER3* is a member of the Period family of genes and is expressed in a circadian pattern in the suprachiasmatic nucleus, the primary circadian pacemaker in the mammalian brain. Genes in this family encode components of the circadian rhythms of locomotor activity, metabolism and behavior. *PER3* is upregulated by *CLOCK/ARNTL* heterodimers but then represses this up-regulation in a feedback loop using *PER/CRY* heterodimers to interact with *CLOCK/ARNTL*. *RORA* is a member of the NR1 subfamily of nuclear hormone receptors and aids in the transcriptional regulation of some genes involved in circadian rhythm. Rs228697 in *PER3* has been linked to morningness–eveningness preference and circadian rhythm sleep disorders, although the length polymorphism/VNTR in *PER3* has been linked to stress response and bipolar disorder..^26, 27, 28^ In addition, rs12137927 (the same SNP implicated in our study) and rs228644 from *PER3* and rs11632098 from *RORA* were reportedly linked to endorsing the presence of both a modest number (>2 to <6) and a high number of depressive symptoms (⩾6) on the Geriatric Depression Scale as compared with endorsing none-few depressive symptoms (0–2).^29^ The variant with association signal of *P*<0.0001 in the *NR1D1* interval is variant rs10305315, located downstream of *IGFBP4* (*P*=5.99 × 10^−5^). It remains unclear whether rs10305315 affects the function of *NR1D1* (no eQTL evidence from GTEx portal based on GTEx Analysis Release V6). *NR1D1* encodes a ligand-sensitive transcription factor that negatively regulates the expression of core clock proteins. The expression of this gene represses the circadian clock transcription factor aryl hydrocarbon receptor nuclear translocator-like protein 1 (*ARNTL*).

Variants from glutamate receptor, ionotropic, delta 2 (*GRID2*, rs76800659 *P*=4.21 × 10^−5^), glutamate receptor, metabotropic 1 (*GRM1*, rs2328741 *P*=1.12 × 10^−5^; rs2268666 *P*=4.75 × 10^−6^), glutamate receptor, metabotropic 5 (*GRM5*, rs308873 *P*=2.24 × 10^−5^), corticotropin releasing hormone receptor 1 (*CRHR1*) and protein kinase C, alpha (*PRKCA*, rs34337960 *P*=1.49 × 10^−5^) were accountable for LTD gene set enrichment. l-glutamate is the major excitatory neurotransmitter in the central nervous system and activates both ionotropic and metabotropic glutamate receptors. Glutamatergic neurotransmission is involved in most aspects of normal brain function and putatively is perturbed in many neuropathological conditions. In particular, glutamate has been implicated in the pathophysiology and treatment of mood disorders, particularly with respect to altered transmission in limbic–thalamocortical circuits.^30^ Rs2268666 in *GRM1* was shown to be associated with unipolar depression (UPD) phenotype in a discovery cohort of 350 patients and 370 matched controls (*P*=7.0 × 10^−5^ in allelic test and *P*=0.0002 in genotypic test with T being the risk allele, both passing multiple testing correction threshold), and was partially replicated in an independent cohort of 904 patients and 1012 controls (genotypic test *P*=0. 02, allelic test *P*=0.59).^31^ Furthermore, patients homozygous for the non-risk genotypes (C/C) showed reduced hippocampal glutamate levels as measured by ¹H-MR-spectroscopy, a more pronounced normalization of HPA-axis hyperactivity using a combined dexamethasone suppression/CRH-challenge (DEX/CRH) test,^32^ and a better antidepressant treatment outcome.^31^ Likewise, patients with each copy of C allele of rs2268666 also showed an increased likelihood of being bupropion responders in this study (*P*=4.75 × 10^−6^, OR=1.22). Knockout mice mGluR5(-/-) displayed more depression-like behaviors (learned helplessness, social withdrawal and anhedonia) than control mice following exposure to stressful stimuli, whereas lentiviral 'rescue' of mGluR5 in the nucleus accumbens decreased these depression-like behaviors in mGluR5(-/-) mice.^33^ mGluR5 may be involved in the regulation of neural network activity and synaptic plasticity. d-Serine is an endogenous co-agonist for N-methyl-d-aspartate receptors and regulates neurotransmission and synaptic plasticity including long-term potentiation and LTD.^34, 35^ d-Serine serves as an endogenous ligand for GluD2, the predominant excitatory neurotransmitter receptors in the mammalian brain, to regulate LTD at synapses.

The VEGF pathway is another gene set enriched in bupropion response analysis. The neurotrophic hypothesis of MDD hypothesizes that the neuropathology of MDD involves a downregulation of neurotrophin signaling, involving both brain-derived neurotrophic factor and the multi-competent angiogenic and neurogenic molecule VEGF in hippocampal neurogenesis. A recent meta-analysis of fourteen studies (*n*=1633) showed that VEGF levels were significantly elevated in individuals with MDD when compared to healthy controls,^36^ supporting that this mediator may be involved in neuroplasticity mechanisms underlying or compensating for the pathophysiology of MDD. Variants from fms-related tyrosine kinase 4 (*FLT4*, rs189869480, *P*=6.12 × 10^−5^), *PRKCA*, and phospholipase C, gamma 1(*PLCG1*, rs56012336, *P*=2.05 × 10^−5^) was accountable for VEGF pathway enrichment in the bupropion response GWAS. The protein encoded by *PLCG1* catalyzes the formation of inositol 1, 4, 5-trisphosphate and diacylglycerol from phosphatidylinositol 4, 5-bisphosphate, and is a major enzyme of the phosphatidylinositol second messenger system. Polymorphisms in *PLCG1*A were associated with bipolar disorder^37^ and response to lithium.^38, 39^

Many variants from candidate gene studies and GWAS analyses have been associated with antidepressant treatment response with various strength of association in mostly small sample sizes. Findings of selected variants reported from prior candidate gene and GWAS studies are discussed in Supplementary Text S3.

The consistent heritability estimates between epidemiologically ascertained depression samples and PGC1 MDD samples (which in large part were clinically ascertained) and the high genetic correlation estimated between our phenotypes and PGC1 MDD patients suggest that self-reported samples do not significantly bias the recruitment of diagnostic class, although the self-reported samples are certainly more heterogeneous and may include both MDD and minor depressive disorder samples. The apparent overlap in genetic architecture between drug/class-specific and antidepressant-wide responders/non-responders is not surprising given that these research participants have a history of self-reported depression. There are inherent challenges of interpreting single arm retrospective study where self-reported outcome assessment is the only data collection modality. These challenges include lack of diagnostic certainty, recall biases and qualitative nature of outcome assessment, and whether patients are optimally dosed and medication compliance for minimal dose exposure (for example, 6 weeks). The Antidepressant Efficacy and Side Effect Survey contained a question ‘For how long did you take Wellbutrin/bupropion?' with the following four answers ‘Less than 4 months; 4 months–1 year; 1–5 years; and Over 5 years'. The shortest time duration <4 months will not allow us to disentangle minimal medication exposure of 6 weeks. However, among the ~2500 patients reporting efficacious bupropion response, only ~150 (~6%) took the medication for <4 months. In contrast, among the ~1800 patients who reported lack of efficacy to bupropion, ~830 (46%) took the medication for longer than 4 months.

This study grouped escitalopram and citalopram into one analysis group to increase the sample size. Citalopram is a racemic mixture of a pharmacologically active *S*-enantiomer (that is, escitalopram) and the R-enantiomer, which is putatively inactive. The study explored both drug-specific and class-specific effects and focused on the groups with the largest sample sizes as well as on the treatment-resistant subgroup. Despite the interesting finding from the bupropion GWAS analysis and the consistency (though not significant at the genome-wide significance threshold) between this study and previously reported studies for key MDD, pharmacokinetics, and pharmacodynamics genes relevant to antidepressant efficacy response, the genetic variants alone are unlikely to deliver clinically actionable predictive diagnostic tests. A more comprehensive approach using a composite signature of predictors ultimately may be required to predict treatment outcome to a particular drug class with sufficient sensitivity and specificity to warrant its use in the clinic.

## Figures and Tables

**Table 1:** Index SNPs for strongest associations for responders vs non-responders and non-TRD vs TRD

*Cytoband*	*SNP*	*CHR*	*BP*	*Alleles*	P*-value*	*OR*	*95% CI*	*Gene context*
*NDRI responders vs non-responders*								
4q22.1	rs1908557	4	90421353	C/T	2.6 × 10^−8^	1.348	1.213, 1.497	GPRIN3—-[]—-SNCA
14q13.2	rs77945277	14	35310175	A/G	2.3 × 10^−7^	0.09	0.031, 0.261	[BAZ1A]
1p13.3	rs4839421	1	111021960	A/C	4.5 × 10^−7^	1.249	1.146, 1.362	PROK1—[]—KCNA10
11p12	rs75995702	11	42806727	C/G	7.3 × 10^−7^	12.857	3.496, 47.288	[]—-API5
8p23.2	rs34102224	8	5222028	C/G	1.3 × 10^−6^	1.354	1.197, 1.531	CSMD1—-[]

*SSRI responders* *vs* *non-responders*								
5p13.1	rs80164876	5	38464244	A/G	4.1 × 10^−7^	2.09	1.551, 2.815	[EGFLAM]
22q11.22	rs114465512	22	22402293	C/T	5.2 × 10^−7^	0.077	0.023, 0.251	TOP3B—[]—-VPREB1
14q32.12	rs35863382	14	91938876	C/T	6.9 × 10^−7^	0.649	0.547, 0.768	[SMEK1]
18q21.1	rs117198528	18	47220558	G/T	9.4 × 10^−7^	1.823	1.437, 2.313	LIPG—-[]—ACAA2
19q13.33	rs138472420	19	48161022	A/G	1.3 × 10^−6^	5.628	2.692, 11.767	[GLTSCR1]

*Citalopram or escitalopram responders* *vs* *non-responders*								
1p21.2	rs111365677	1	99929134	C/T	1.4 × 10^−7^	0.057	0.014, 0.231	LPPR4—-[]—-PALMD
3q23	rs142484554	3	140778295	D/I	3.3 × 10^−7^	0.685	0.592, 0.794	[SPSB4]
10q24.33	rs188843168	10	105149941	C/T	4.0 × 10^−7^	449.83	5.402, Inf	[USMG5]
2p13.3	rs6546604	2	70642807	A/G	5.3 × 10^−7^	0.782	0.709, 0.861	FAM136A—-[]—TGFA
4p14	rs34177316	4	40987299	D/I	6.4 × 10^−7^	1.355	1.203, 1.528	[APBB2]
11q24.1	rs201921722	11	123550707	D/I	6.6 × 10^−7^	0.791	0.721, 0.868	SCN3B—[]—ZNF202
6p12.3	rs80278479	6	50726179	C/G	7.6 × 10^−7^	0.069	0.018, 0.258	[TFAP2D]

*Non-TRD* *vs TRD*								
2p22.3	rs1375194	2	33826877	C/T	2.4 × 10^−7^	0.795	0.729, 0.867	FAM98A-[]—-MYADML
2q14.1	rs190662943	2	115952585	A/G	7.1 × 10^−7^	14.419	5.177, 40.161	[DPP10]
1q42.13	rs75507262	1	229349484	A/G	1.1 × 10^−6^	0.43	0.312, 0.592	RHOU—-[]—RAB4A
12q24.32	rs10847303	12	127735865	A/G	1.8 × 10^−6^	0.805	0.736, 0.881	[]
20p13	rs73086581	20	3977325	C/T	1.8 × 10^−6^	1.299	1.169, 1.444	[RNF24]

Abbreviations: BP, base pairs; CI, confidence interval; NDRI, norepinephrine–dopamine reuptake inhibitor; OR, odds ratio; SNP, single-nucleotide polymorphism; SSRI, selective serotonin reuptake inhibitor; TRD, treatment-resistant depression.

The table of index SNPs shows information for the most-associated SNP in each associated region, for at least 5 and at most 50 regions for each phenotype. Regions are defined by identifying SNPs with *P*<10^−5^, then grouped into intervals separated by gaps of at least 250 kb. The SNP with smallest *P* within each interval was chosen as index SNP for the region.

**Table 2:** Index SNPs for strongest associations for responders or non-responders vs healthy controls, and non-TRD or TRD vs healthy controls

*Cytoband*	*SNP*	*CHR*	*BP*	*Alleles*	P*-value*	*OR*	*95% CI*	*Gene context*
*NDRI non-responder vs healthy controls*								
Xq27.2	rs190783615	X	141860406	C/T	2.0 × 10^−7^	15.18	3.095, 74.439	MAGEC2—-[]—-SPANXN4
5p13.1	rs201203751	5	39203597	D/I	5.0 × 10^−7^	4.009	2.537, 6.336	[FYB]
17q11.2	rs183124483	17	26437054	A/G	5.9 × 10^−7^	0.067	0.031, 0.146	[NLK]
5p12	rs56388524	5	45757561	C/T	6.8 × 10^−7^	1.798	1.451, 2.228	HCN1—[]
5q12.3	rs143405544	5	64755604	A/G	7.0 × 10^−7^	0.149	0.054, 0.410	[ADAMTS6]
20q12	rs183042538	20	39826060	A/T	7.7 × 10^−7^	1.431	1.249, 1.638	[ZHX3]

*NDRI responders* *vs* *healthy controls*								
12p12.1	rs200855945	12	26277893	D/I	4.4 × 10^−7^	4.929	2.350, 10.340	[BHLHE41]
4q35.1	rs112538845	4	185005628	C/T	5.6 × 10^−7^	1.637	1.366, 1.961	STOX2—[]-ENPP6
2q36.2	rs78087832	2	225464777	C/T	5.7 × 10^−7^	2.731	1.933, 3.857	CUL3—[]—-DOCK10
9q33.1	rs112106319	9	117856818	A/T	1.1 × 10^−6^	0.43	0.294, 0.627	[TNC]
12q21.2	rs73425402	12	77897298	A/T	1.4 × 10^−6^	0.048	0.019, 0.121	E2F7—-[]—-NAV3

*SSRI non-responders* *vs healthy controls*								
11p15.3	rs1994321	11	12087313	G/T	5.2 × 10^−7^	0.866	0.818, 0.916	DKK3—[]—MICAL2
2p25.1	rs113378111	2	9928153	A/G	6.9 × 10^−7^	1.579	1.303, 1.912	YWHAQ—-[]—TAF1B
5q31.1	rs73788091	5	132765209	C/T	1.2 × 10^−6^	3.011	2.044, 4.437	[FSTL4]
18q22.1	rs9951011	18	65033678	A/G	1.8 × 10^−6^	0.847	0.792, 0.906	CDH19—-[]—-DSEL
1q31.1	rs78620960	1	190781499	A/G	2.0 × 10^−6^	0.831	0.771, 0.896	FAM5C—-[]

*SSRI responders vs healthy controls*								
4q31.23	rs150175932	4	151022647	C/T	1.0 × 10^−7^	15.22	3.318, 69.804	[DCLK2]
8q12.1	rs141746753	8	57846713	C/T	1.2 × 10^−6^	0.308	0.175, 0.544	PENK—-[]—IMPAD1
7q31.33	rs73720034	7	125435049	C/T	1.3 × 10^−6^	1.256	1.142, 1.381	POT1—-[]—-GRM8
2p16.1	rs6545694	2	58847953	A/G	1.7 × 10^−6^	1.096	1.056, 1.137	FANCL—-[]
2p25.3	rs61519662	2	2700391	C/T	2.4 × 10^−6^	0.908	0.872, 0.945	MYT1L—-[]—-TSSC1

*Citalopram or escitalopram non-responders vs healthy controls*								
20q13.13	rs6063349	20	47681882	C/G	6.9 × 10^−7^	0.845	0.790, 0.903	[CSE1L]
11p13	rs142641502	11	33131407	C/T	8.4 × 10^−7^	4.565	2.155, 9.671	[CSTF3]
6p25.3	rs201569130	6	1403150	D/I	8.6 × 10^−7^	3.009	1.785, 5.073	FOXF2-[]—-FOXC1
15q23	rs1548076	15	70226623	A/G	1.2 × 10^−6^	0.401	0.262, 0.615	RPLP1—-[]—-TLE3
11q13.2	rs182377406	11	67216849	A/G	1.3 × 10^−6^	5.773	3.250, 10.254	CORO1B-[]-GPR152

*Citalopram or escitalopram responders vs healthy controls*								
11q14.1	rs74860738	11	80382727	A/G	3.2 × 10^−7^	0.777	0.708, 0.854	[]
20p11.23	rs143934587	20	19146450	A/G	6.5 × 10^−7^	0.149	0.080, 0.277	C20orf78—-[]—SLC24A3
4q12	rs189864513	4	53633557	C/T	7.8 × 10^−7^	0.081	0.037, 0.177	ERVMER34-1—[]—RASL11B
3q27.3	rs11924809	3	186071445	A/G	1.3 × 10^−6^	1.215	1.122, 1.316	[DGKG]
10q24.33	rs117375960	10	104921664	A/C	1.5 × 10^−6^	0.007	0.000, 0.345	[NT5C2]

*TRD vs healthy controls*								
21q22.2	rs190544851	21	39732396	G/T	2.8 × 10^−7^	14.29	3.555, 57.454	KCNJ15—[]—ERG
6q25.3	rs57043326	6	159314933	A/G	8.2 × 10^−7^	0.377	0.240, 0.593	[C6orf99]
1p36.21	rs12068879	1	15286356	A/G	1.1 × 10^−6^	2.221	1.667, 2.958	[KAZN]
9p23	rs1322281	9	10582445	C/T	1.4 × 10^−6^	1.242	1.139, 1.354	[PTPRD]
2p22.3	rs13418410	2	33800899	A/C	1.6 × 10^−6^	1.212	1.121, 1.311	RASGRP3—[]-FAM98A

*Non-TRD vs healthy controls*								
12q13.13	rs34807503	12	51919133	D/I	4.5 × 10^−7^	0.917	0.887, 0.948	SLC4A8-[]—SCN8A
7q32.2	rs2402960	7	129405774	C/T	7.5 × 10^−7^	0.91	0.877, 0.945	NRF1-[]—UBE2H
16q21	rs200312707	16	62065679	D/I	1.4 × 10^−6^	0.919	0.888, 0.951	[CDH8]
1p36.23	rs400736	1	8078309	C/T	1.9 × 10^−6^	0.921	0.891, 0.953	[ERRFI1]
1p32.3	rs35265457	1	54273279	C/T	2.0 × 10^−6^	1.508	1.261, 1.803	[NDC1]

Abbreviations: BP, base pairs; CI, confidence interval; NDRI, norepinephrine–dopamine reuptake inhibitor; OR, odds ratio; SNP, single-nucleotide polymorphism; SSRI, selective serotonin reuptake inhibitor; TRD, treatment-resistant depression.

The table of index SNPs shows information for the most-associated SNP in each associated region, for at least 5 and at most 50 regions for each phenotype. Regions are defined by identifying SNPs with *P*<10^−5^, then grouped into intervals separated by gaps of at least 250 kb. The SNP with smallest *P* within each interval was chosen as index SNP for the region.

**Table 3:** Heritability and genetic correlation estimates

*Analysis group*	*h^2^ liability (s.e.)*	*Genetic correlation (s.e)*												
		*C*	*D*	*E*	*F*	*G*	*H*	*I*	*J*	*K*	*L*	*M*	*N*	*O*
NDRI responders vs non-responders (A)	−0.0541 (0.088)	NE	NE	NE	NE	NE	NE	NE	NE	NE	NE	NE	NE	NE
SSRI responders vs non-responders (B)	0.0456 (0.0498)	0.4414 (0.4605)	0.9475 (0.4126)*	−0.3919 (0.4517)	−0.4925 (0.1647)**	−0.3694 (0.3175)	−0.732 (0.35)*	−0.2222 (0.35)	−0.1014 (0.3363)	−0.0785 (0.3414)	−0.1453 (0.2993)	−0.8816 (1.1096)	−0.4615 (0.4316)	−0.3535 (0.3976)
citalopram or escitalopram responders vs non-responders (C)	0.1201 (0.0925)		0.3214 (0.5089)	−0.1525 (0.3902)	0.0032 (0.27)	−0.2333 (0.2966)	−0.0187 (0.3484)	−0.1311 (0.2915)	0.2141 (0.2024)	0.4319 (0.2358)	0.2783 (0.1884)	−0.1914 (0.3263)	−0.4745 (0.327)	−0.1221 (0.2005)
non-TRD vs TRD (D)	0.11 (0.0995)			−0.4937 (0.3368)	−0.334 (0.2569)	−0.018 (0.3735)	−0.6184 (0.1377)***	−0.3437 (0.3974)	0.0517 (0.2409)	0.2372 (0.2739)	−0.0087 (0.2421)	−0.8074 (0.6182)	NE	NE
NDRI non-responder vs healthy controls (E)	0.1492 (0.0617)				1.0662 (0.2294)***	1.051 (0.2761)***	1.0813 (0.2223)***	NE	1.0858 (0.2431)***	0.8947 (0.2685)***	1.0143 (0.2351)***	0.9592 (0.3818)*	0.1734 (0.1697)	0.426 (0.1849)*
SSRI non-responders vs healthy controls (F)	0.1781 (0.0323)					0.9621 (0.0906)***	1.0064 (0.1315)***	0.9651 (0.1735)***	0.9379 (0.1372)***	0.9 (0.1518)***	0.9622 (0.1236)***	1.0192 (0.1987)***	0.2945 (0.1154)*	0.2426 (0.094)**
citalopram or escitalopram non-responders vs healthy controls (G)	0.1946 (0.0639)						0.7342 (0.1935)***	1.0803 (0.2411)***	0.9699 (0.2021)***	0.8184 (0.2264)***	0.9351 (0.1831)***	1.021 (0.3517)**	0.4085 (0.2012)*	0.1689 (0.1329)
TRD vs healthy controls (H)	0.1673 (0.0537)							1.0835 (0.2958)***	0.8264 (0.2083)***	0.6599 (0.2267)**	0.8811 (0.2046)***	1.1634 (0.3555)**	0.091 (0.1904)	0.5946 (0.2576)*
NDRI responders vs healthy controls (I)	0.2211 (0.0607)								0.9303 (0.1358)***	0.9131 (0.1791)***	1.0013 (0.1117)***	0.778 (0.2128)***	0.1861 (0.1385)	0.0678 (0.1119)
SSRI responders vs healthy controls (J)	0.1441 (0.0201)									0.9667 (0.0594)***	0.9899 (0.0232)***	0.697 (0.1393)***	0.0926 (0.0883)	0.1575 (0.0791)*
citalopram or escitalopram responders vs healthy controls (K)	0.1621 (0.0354)										0.9871 (0.0718)***	0.5962 (0.1455)***	0.0101 (0.096)	0.0897 (0.1024)
non-TRD vs healthy controls (L)	0.141 (0.0151)											0.6504 (0.1241)***	0.0529 (0.0833)	0.1063 (0.0695)
PGC MDD (M)	0.1894 (0.0354)												0.5551 (0.1014)***	0.472 (0.0892)***
PGC BP (N)	0.2848 (0.0286)													0.6561 (0.0605)***
PGC SCZ (O)	0.3227 (0.0248)													

Abbreviations: MDD, major depressive disorder; NDRI, norepinephrine–dopamine reuptake inhibitor; NE, not estimable; SSRI, selective serotonin reuptake inhibitor; TRD, treatment-resistant depression.

*P<0.05; **P<0.01; ***P<0.001.

*h*^2^ estimate assumes population prevalence rate of 8% for responder/non-responders/TRD/non-TRD groups, 15% for MDD, and 1% for bipolar and schizophrenia, respectively.

**Table 4:** Pathway Enrichment of bupropion response vs non-response (p_corr_ < 0.05)

*Gene set collection*	P_*T*_	R^*2*^	P_*corr*_	*T_Size*	*Int_No*	P	*Gene set*
*NDRI responders* vs *non-responders*							
kegg.set	0.0001	0.5	0.0381924	22	3	0.00049995	04710 Circadian_rhythm_-_mammal
kegg.set	0.00005	0.2	0.0381924	67	5	0.00119988	04730 Long-term_depression
kegg.set	0.00005	0.3	0.0421916	67	5	0.00139986	04730 Long-term_depression
kegg.set	0.00005	0.5	0.0107978	67	5	0.00029997	04730 Long-term_depression
c2.cp.biocarta.v5.0.entrez.gmt.msig.set	0.0001	0.3	0.0381924	27	3	0.0009999	BIOCARTA_VEGF_PATHWAY http://www.broadinstitute.org/gsea/msigdb/cards/BIOCARTA_VEGF_PATHWAY
c2.cp.biocarta.v5.0.entrez.gmt.msig.set	0.0001	0.5	0.0405919	27	3	0.00089991	BIOCARTA_VEGF_PATHWAY http://www.broadinstitute.org/gsea/msigdb/cards/BIOCARTA_VEGF_PATHWAY
c2.cp.kegg.v5.0.entrez.gmt.msig.set	0.00005	0.2	0.0357928	67	5	0.0009999	KEGG_LONG_TERM_DEPRESSION http://www.broadinstitute.org/gsea/msigdb/cards/KEGG_LONG_TERM_DEPRESSION
c2.cp.kegg.v5.0.entrez.gmt.msig.set	0.00005	0.5	0.0171966	67	5	0.00049995	KEGG_LONG_TERM_DEPRESSION http://www.broadinstitute.org/gsea/msigdb/cards/KEGG_LONG_TERM_DEPRESSION
c2.cp.v5.0.entrez.gmt.msig.set	0.00005	0.2	0.0369926	25	3	0.00019998	PID_CDC42_REG_PATHWAY http://www.broadinstitute.org/gsea/msigdb/cards/PID_CDC42_REG_PATHWAY
c2.cp.v5.0.entrez.gmt.msig.set	0.00005	0.3	0.0379924	25	3	0.00019998	PID_CDC42_REG_PATHWAY http://www.broadinstitute.org/gsea/msigdb/cards/PID_CDC42_REG_PATHWAY
c3.mir.v5.0.entrez.gmt.msig.set	0.00005	0.2	0.04999	85	5	0.0009999	GGCCAGT,MIR-193A,MIR-193B http://www.broadinstitute.org/gsea/msigdb/cards/GGCCAGT,MIR-193A,MIR-193B
c5.mf.v5.0.entrez.gmt.msig.set	0.00005	0.2	0.0419916	45	3	0.00159984	MOLECULAR_ADAPTOR_ACTIVITY http://www.broadinstitute.org/gsea/msigdb/cards/MOLECULAR_ADAPTOR_ACTIVITY
c5.mf.v5.0.entrez.gmt.msig.set	0.00005	0.2	0.035193	39	3	0.00119988	SH3_SH2_ADAPTOR_ACTIVITY http://www.broadinstitute.org/gsea/msigdb/cards/SH3_SH2_ADAPTOR_ACTIVITY
c5.mf.v5.0.entrez.gmt.msig.set	0.00005	0.5	0.0345931	45	3	0.00119988	MOLECULAR_ADAPTOR_ACTIVITY http://www.broadinstitute.org/gsea/msigdb/cards/MOLECULAR_ADAPTOR_ACTIVITY
c5.mf.v5.0.entrez.gmt.msig.set	0.00005	0.5	0.0303939	55	3	0.00089991	PROTEIN_BINDING_BRIDGING http://www.broadinstitute.org/gsea/msigdb/cards/PROTEIN_BINDING_BRIDGING
c5.mf.v5.0.entrez.gmt.msig.set	0.00005	0.5	0.0237952	39	3	0.00069993	SH3_SH2_ADAPTOR_ACTIVITY http://www.broadinstitute.org/gsea/msigdb/cards/SH3_SH2_ADAPTOR_ACTIVITY
h.all.v5.0.entrez.gmt.msig.set	0.0001	0.5	0.0475905	195	6	0.00279972	HALLMARK_ADIPOGENESIS http://www.broadinstitute.org/gsea/msigdb/cards/HALLMARK_ADIPOGENESIS
c1.all.v5.0.entrez.gmt.msig.set	0.0005	0.2	0.0163967*	34	8	0.00009999	chr8p22 http://www.broadinstitute.org/gsea/msigdb/cards/chr8p22
c1.all.v5.0.entrez.gmt.msig.set	0.0005	0.3	0.0135973*	34	8	0.00009999	chr8p22 http://www.broadinstitute.org/gsea/msigdb/cards/chr8p22
c1.all.v5.0.entrez.gmt.msig.set	0.0005	0.5	0.0167966*	34	8	0.00009999	chr8p22 http://www.broadinstitute.org/gsea/msigdb/cards/chr8p22
c5.bp.v5.0.entrez.gmt.msig.set	0.0005	0.3	0.0413917*	16	5	0.00009999	LIPID_HOMEOSTASIS http://www.broadinstitute.org/gsea/msigdb/cards/LIPID_HOMEOSTASIS
c5.bp.v5.0.entrez.gmt.msig.set	0.0005	0.5	0.0403919	16	5	0.00019998	LIPID_HOMEOSTASIS http://www.broadinstitute.org/gsea/msigdb/cards/LIPID_HOMEOSTASIS
