# Supplementary material for: Analysis of 23andMe antidepressant efficacy survey data: implication of circadian rhythm and neuroplasticity in bupropion response
Source: Transl Psychiatry. 2016 Sep 13;6(9):e889–. doi: 10.1038/tp.2016.171 (PMC5048209; doi:10.1038/tp.2016.171)
Supplement: Supplementary Information [file tp2016171x1.docx]

Analysis of 23andMe Antidepressant Efficacy Survey Data: Implication of Circadian Rhythm and Neuroplasticity in Bupropion Response

Qingqin S. Li^1^, Chao Tian^2^, Guy R. Seabrook^3^, Wayne C. Drevets^1^, and Vaibhav A. Narayan^1^

Supplementary Information

Supplement 1

**Figure S1** Manhattan Plots

**Figure S2** Q-Q Plots

**Figure S3** Tissue gene expression pattern

**Table S1** Sample size and basic demographic and genomic control inflation factor

**Table S2** Genes accountable for gene set enrichment in bupropion responders vs. non-responders analysis

**Table S3** Enriched gene sets with corrected P <= 0.05

**Table S4** Exon-specific eQTL findings from BRAINEAC

**Text S1** Genome wide Association Analysis

**Text S2** Possible causative genes near top bupropion hit rs1908557

**Text S3** Discussion of variants reported in the prior candidate gene or GWAS studies

**Text S4** Additional discussions

**Figure S1** Manhattan Plots - the Manhattan plot depicts the distribution of association test statistics versus genomic position, with chromosomes 1 to 22, X, and Y arranged along the X axis. The Y axis represents log-scaled P values. Positions with P<5×10−8 (a score of about 7.3) are shown in red. Loci with smallest P<10−6 are labeled with the name of the nearest gene. A “good” Manhattan plot should show towers of SNPs with small P values supporting most signals that pass the genome wide threshold. (A) NDRI responders vs. non-responders, (B) SSRI responders vs. non-responders, (C) citalopram or escitalopram responders vs. non-responders, (D) non-TRD vs. TRD, (E) NDRI non-responders vs. healthy controls, (F) NDRI responders vs. healthy controls, (G) SSRI non-responders vs. healthy controls, (H) SSRI responders vs. healthy controls, (I) citalopram or escitalopram non-responders vs. healthy controls, (J) citalopram or escitalopram responders vs. healthy controls, (K) TRD vs. healthy controls, and (L) non-TRD vs. healthy controls

## A NDRI responders vs. non-responders (same as Figure 1A)

##
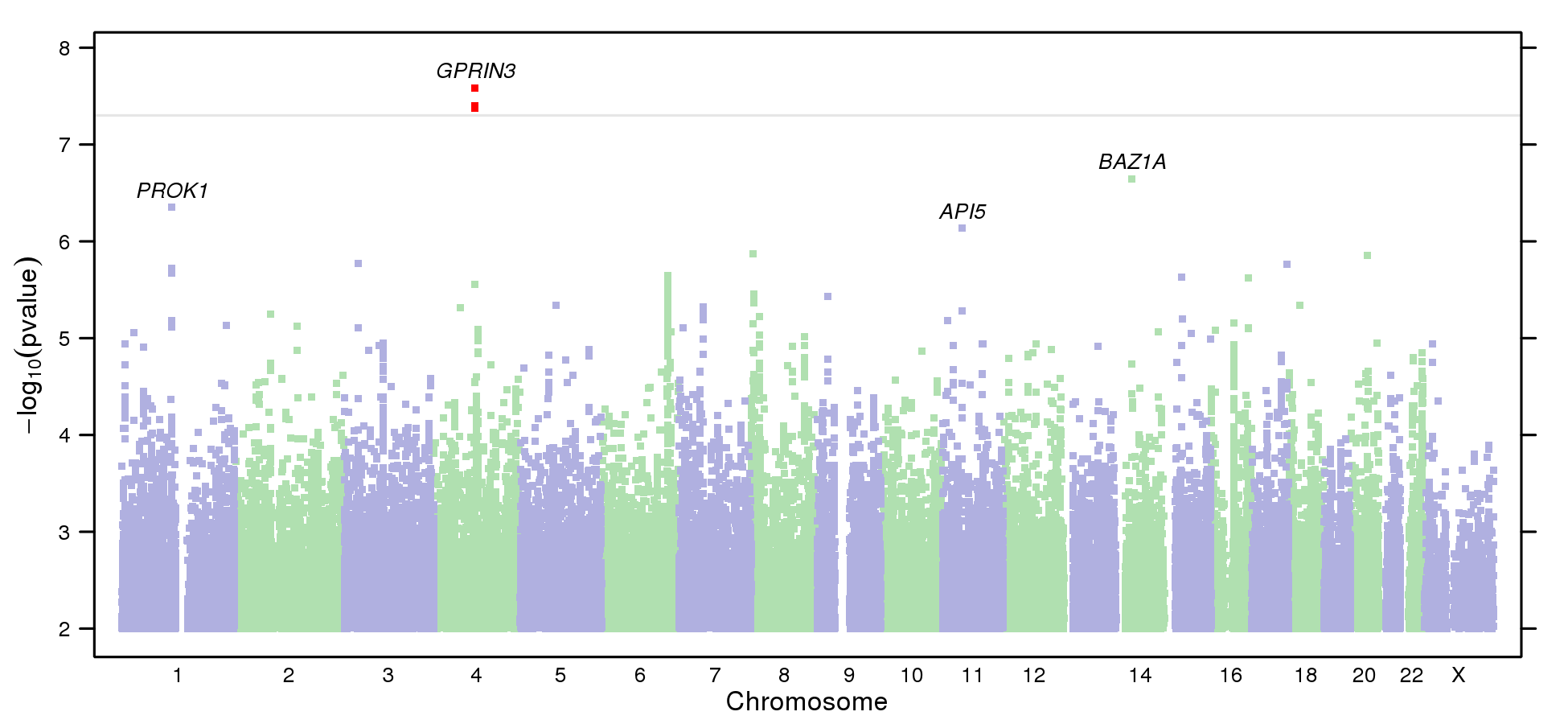


## B SSRI responders vs. non-responders

##
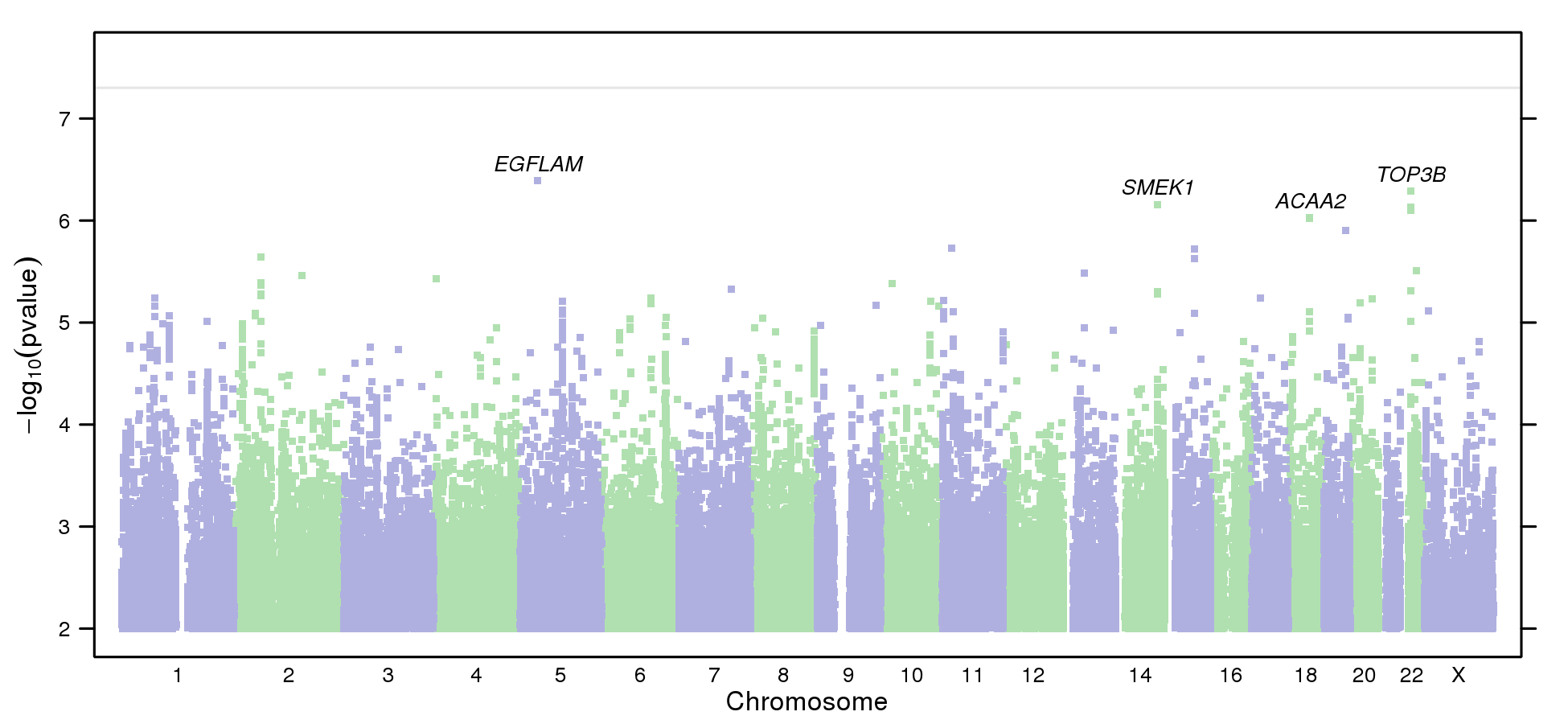


## C citalopram or escitalopram responders vs. non-responders

##
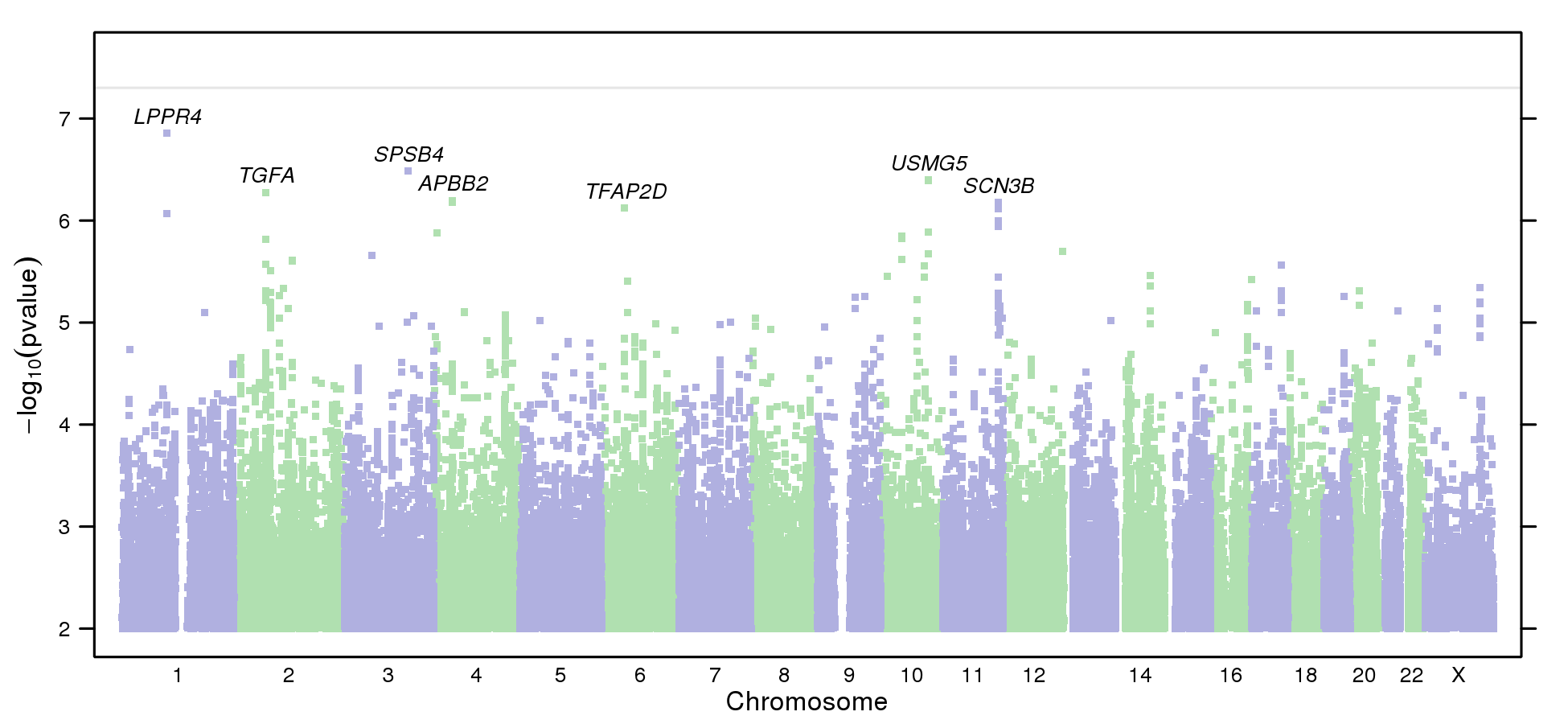


## D non-TRD vs. TRD

##
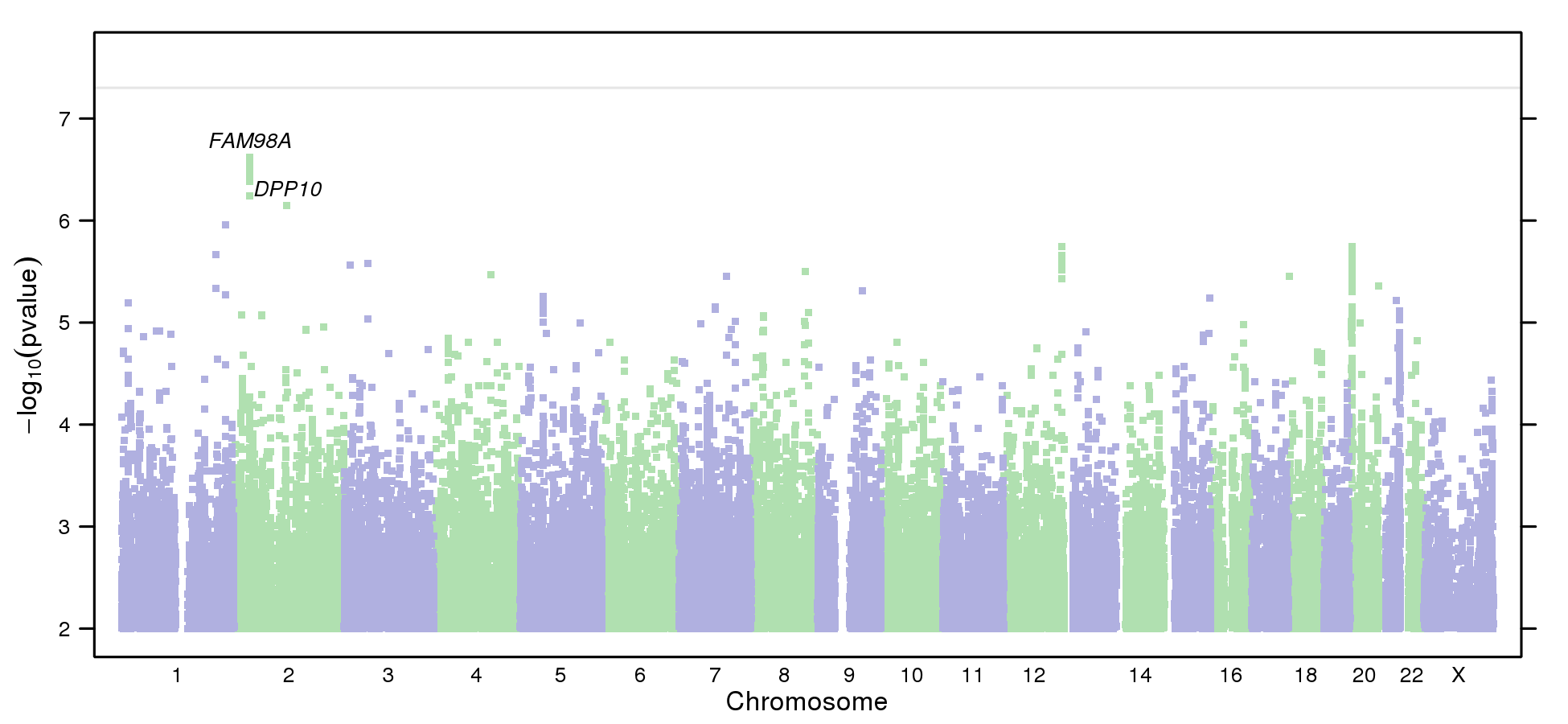


## E NDRI non-responders vs. healthy controls
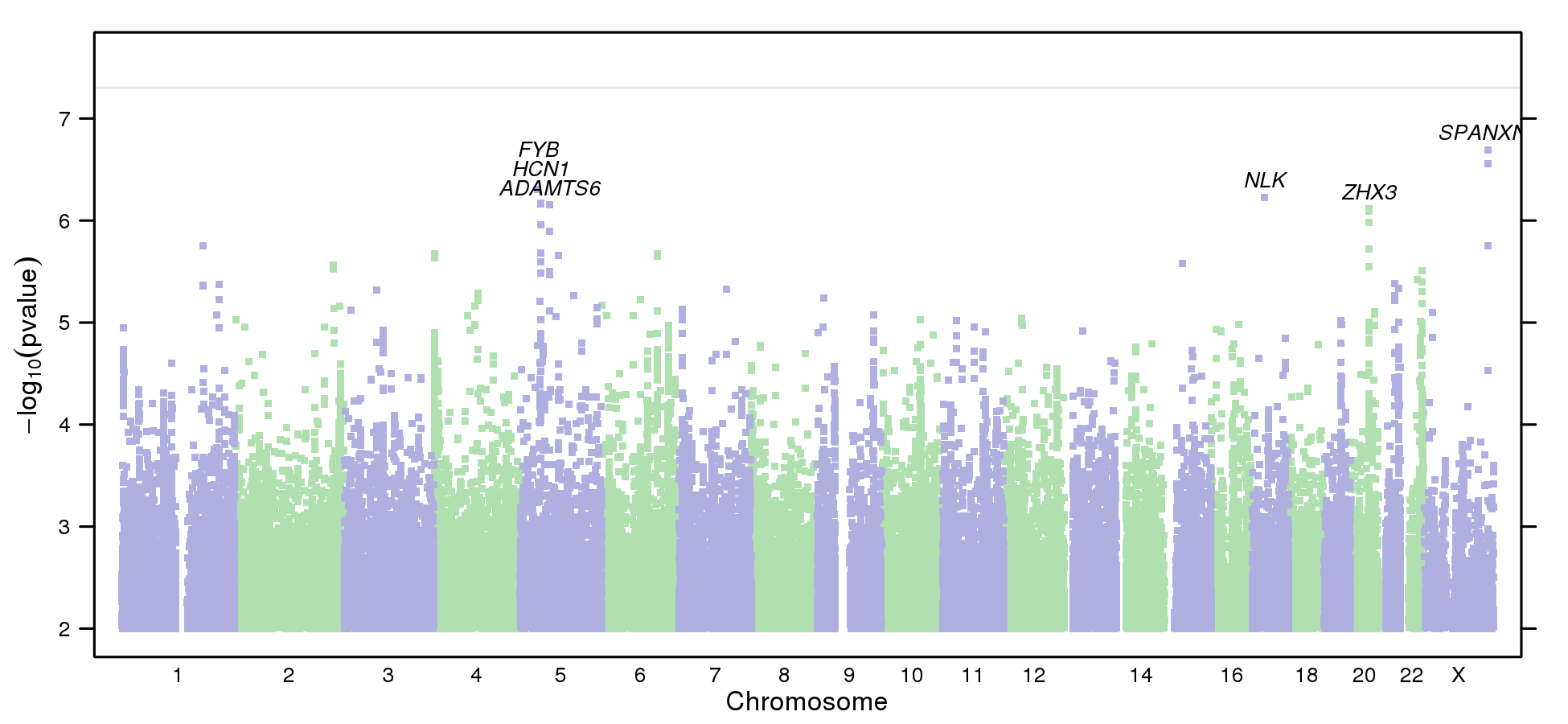


## F NDRI responders vs. healthy controls

##
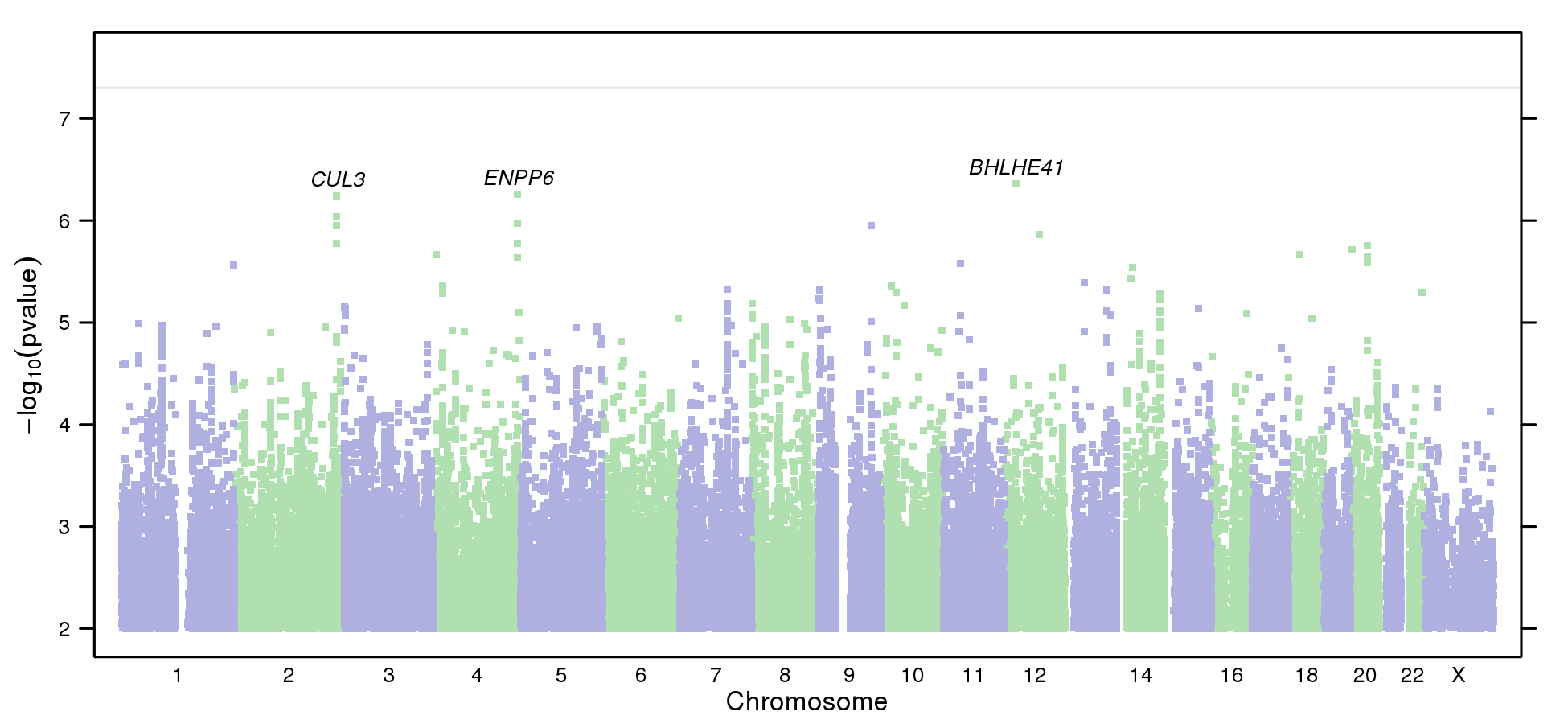


## G SSRI non-responders vs. healthy controls

##
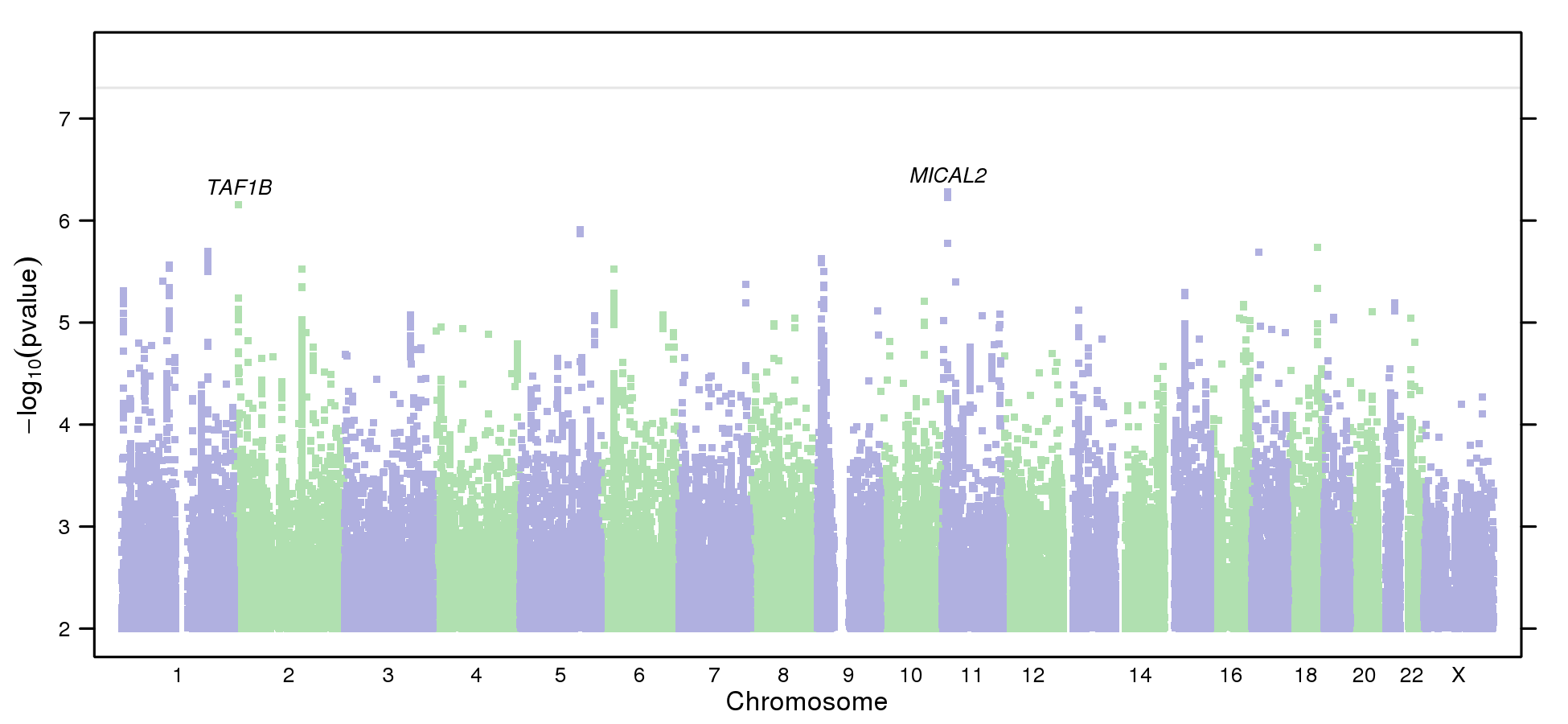


## H SSRI responders vs. healthy controls

##
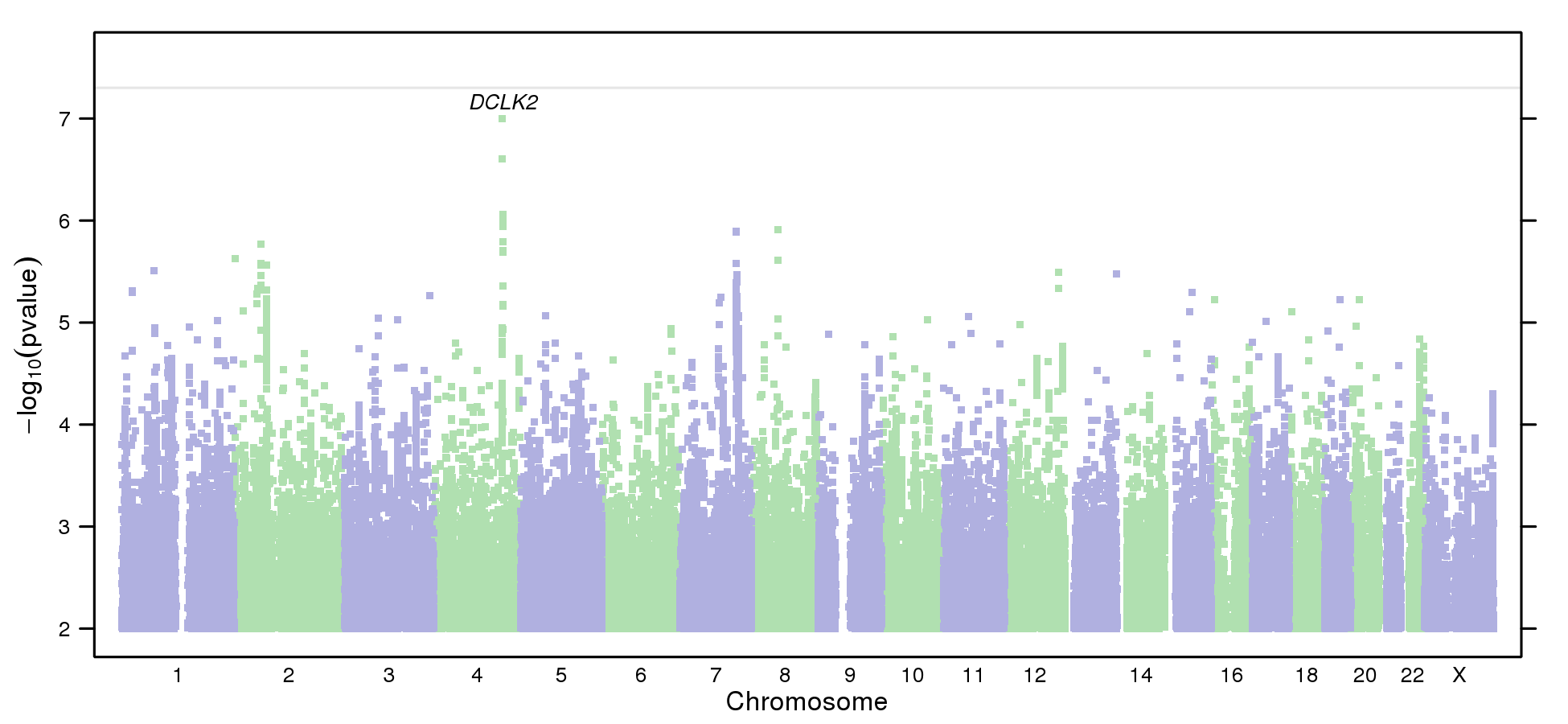


## I citalopram or escitalopram non-responders vs. healthy controls

##
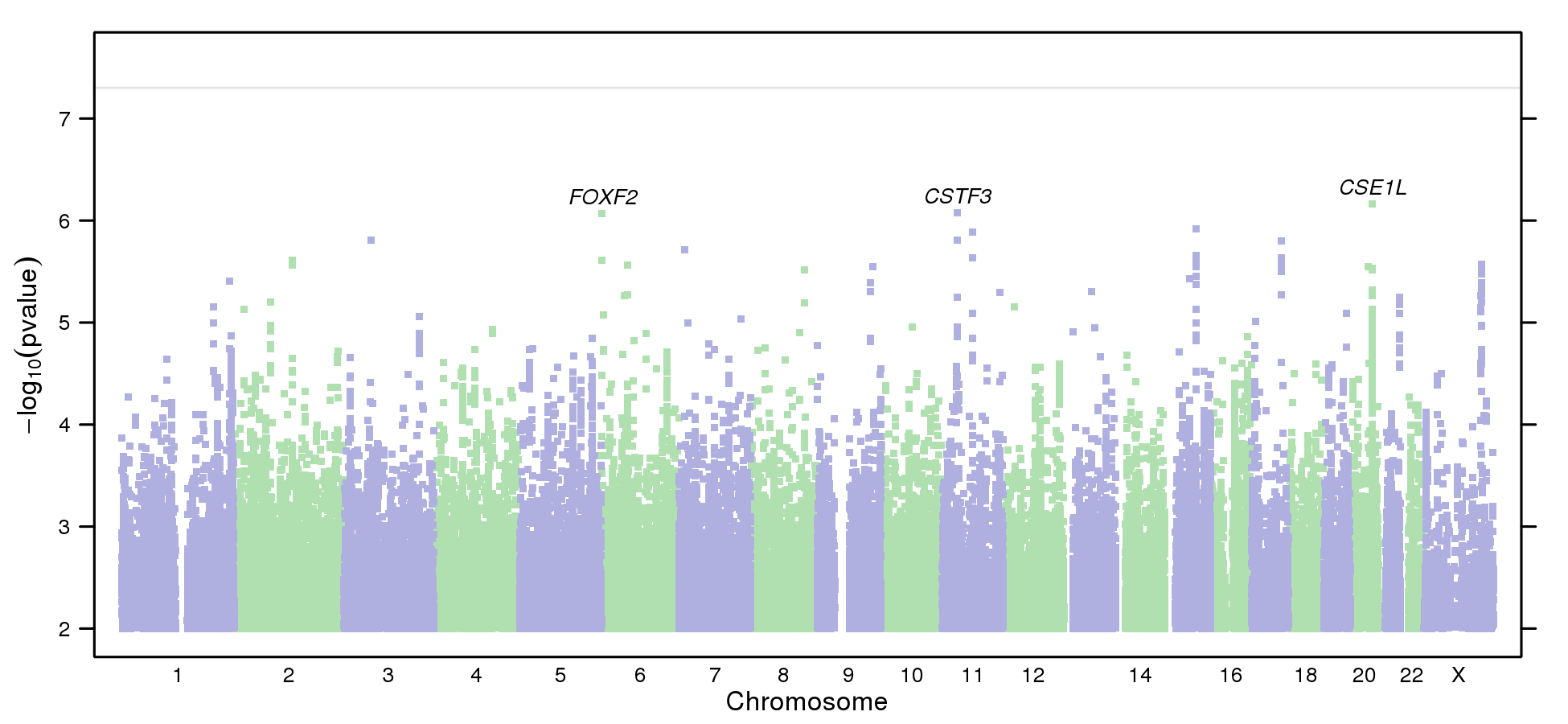


## J citalopram or escitalopram responders vs. healthy controls

##
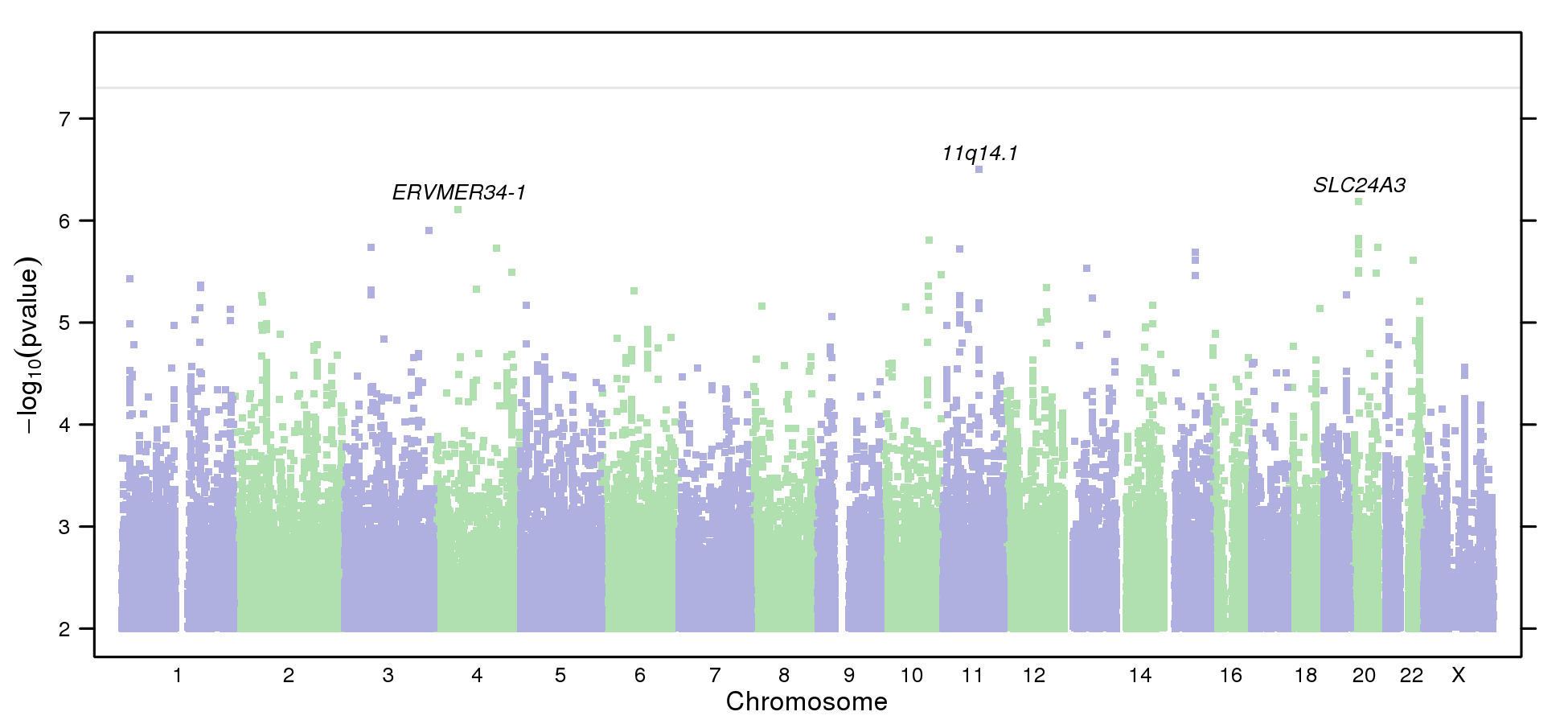


## K TRD vs. healthy controls

##
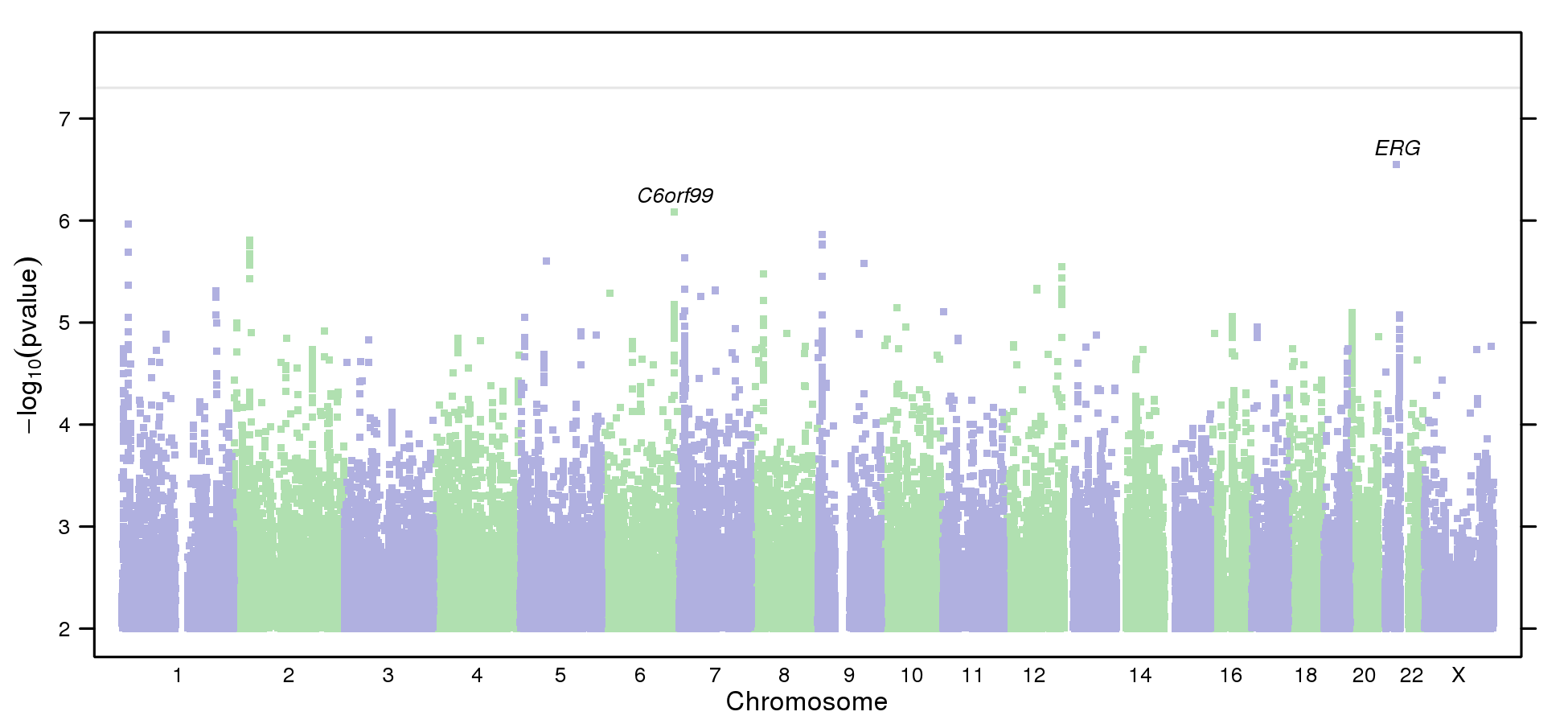


## L non-TRD vs. healthy controls

##
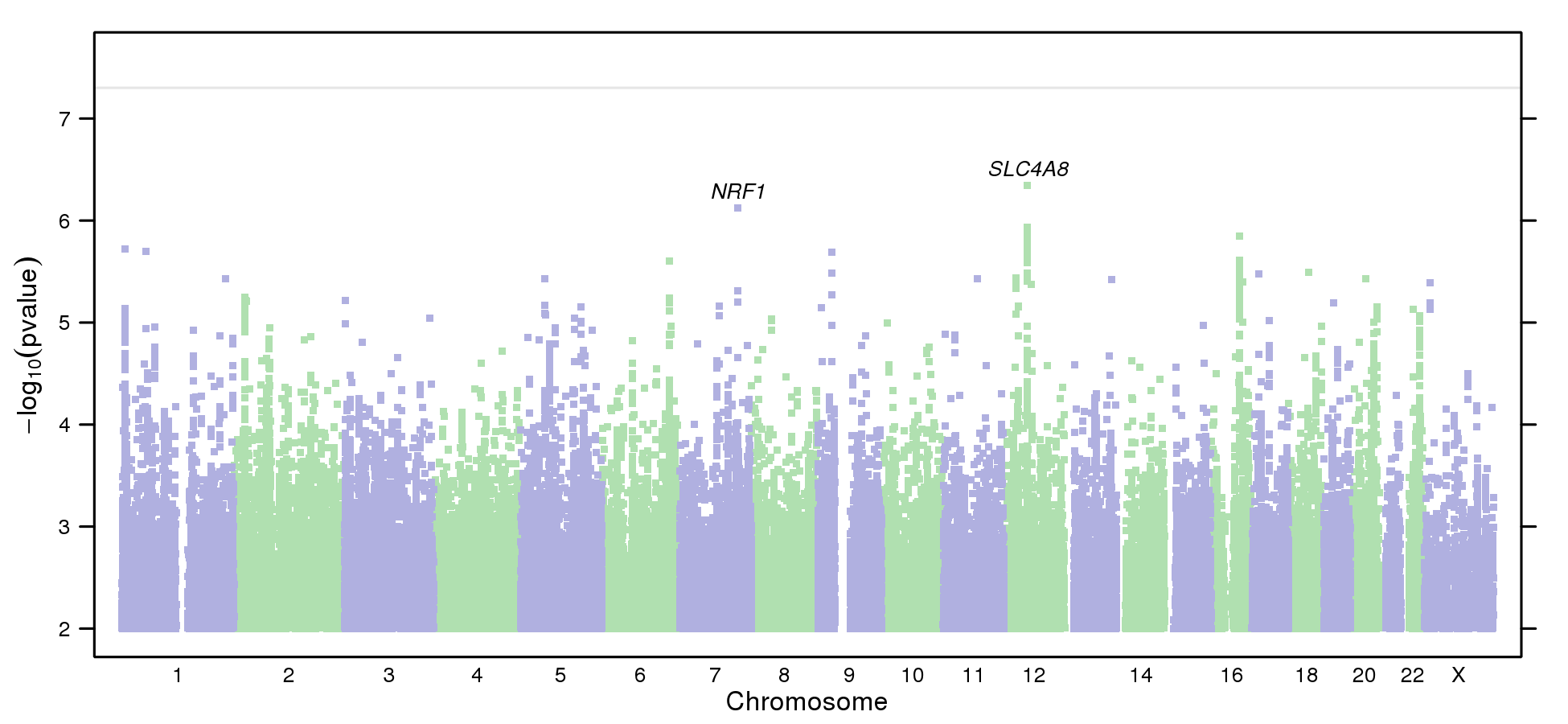


**Figure S2** Q-Q Plots - the Q-Q plot depicts observed versus expected quantiles for the GWAS P values, where the expected distribution of P values is uniform under the null hypothesis, plotted on a log scale. A solid red line is shown with a slope of 1, and dashed red lines represent a 95% confidence envelope under the assumption that the test results are independent. A “good” Q-Q plot follows the null distribution for larger P values (P>0.01) then diverges from the null distribution for small P values. The test statistics in the Q-Q plot have already been adjusted using genomic control. (A) NDRI responders vs. non-responders, (B) SSRI responders vs. non-responders, (C) citalopram or escitalopram responders vs. non-responders, (D) non-TRD vs. TRD, (E) NDRI non-responders vs. healthy controls, (F) NDRI responders vs. healthy controls, (G) SSRI non-responders vs. healthy controls, (H) SSRI responders vs. healthy controls, (I) citalopram or escitalopram non-responders vs. healthy controls, (J) citalopram or escitalopram responders vs. healthy controls, (K) TRD vs. healthy controls, and (L) non-TRD vs. healthy controls.

A


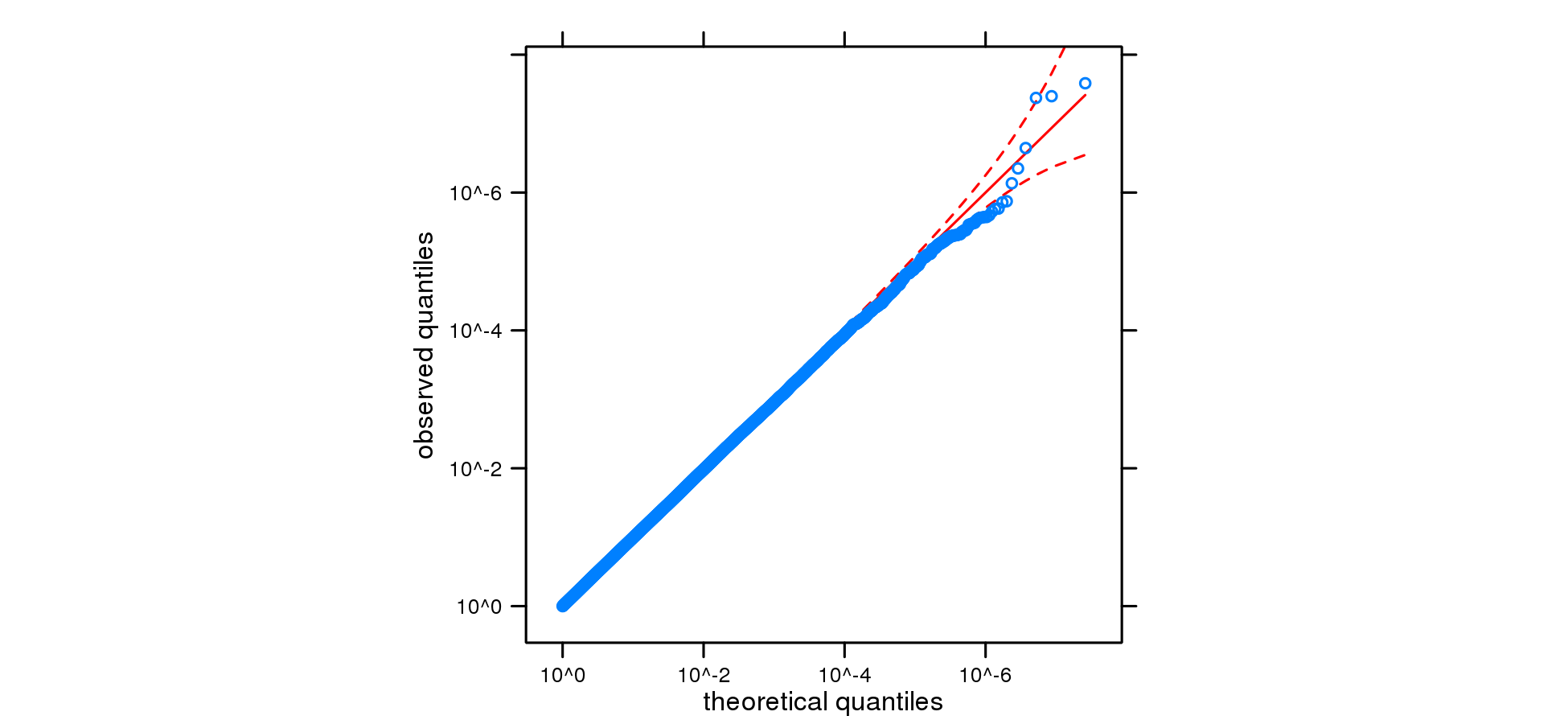


B


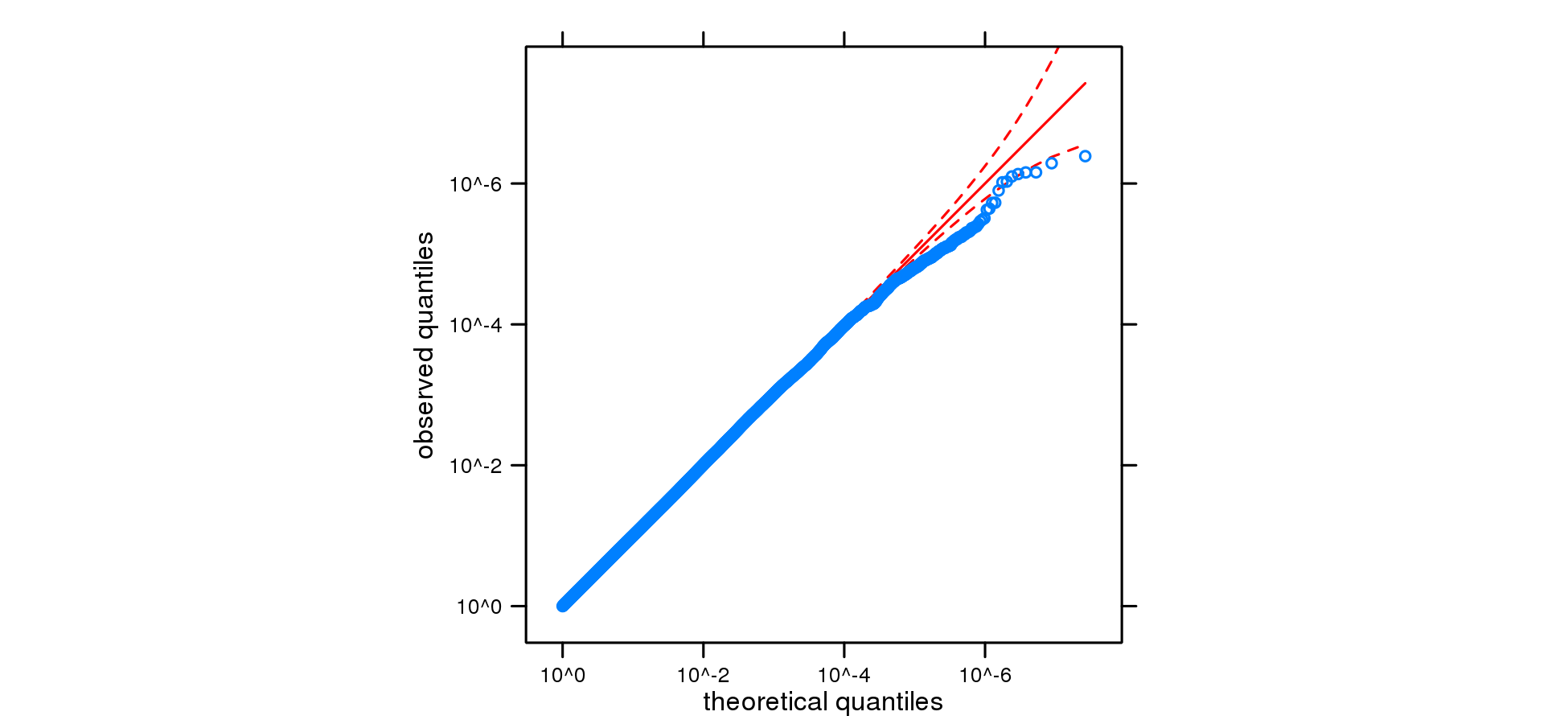


C


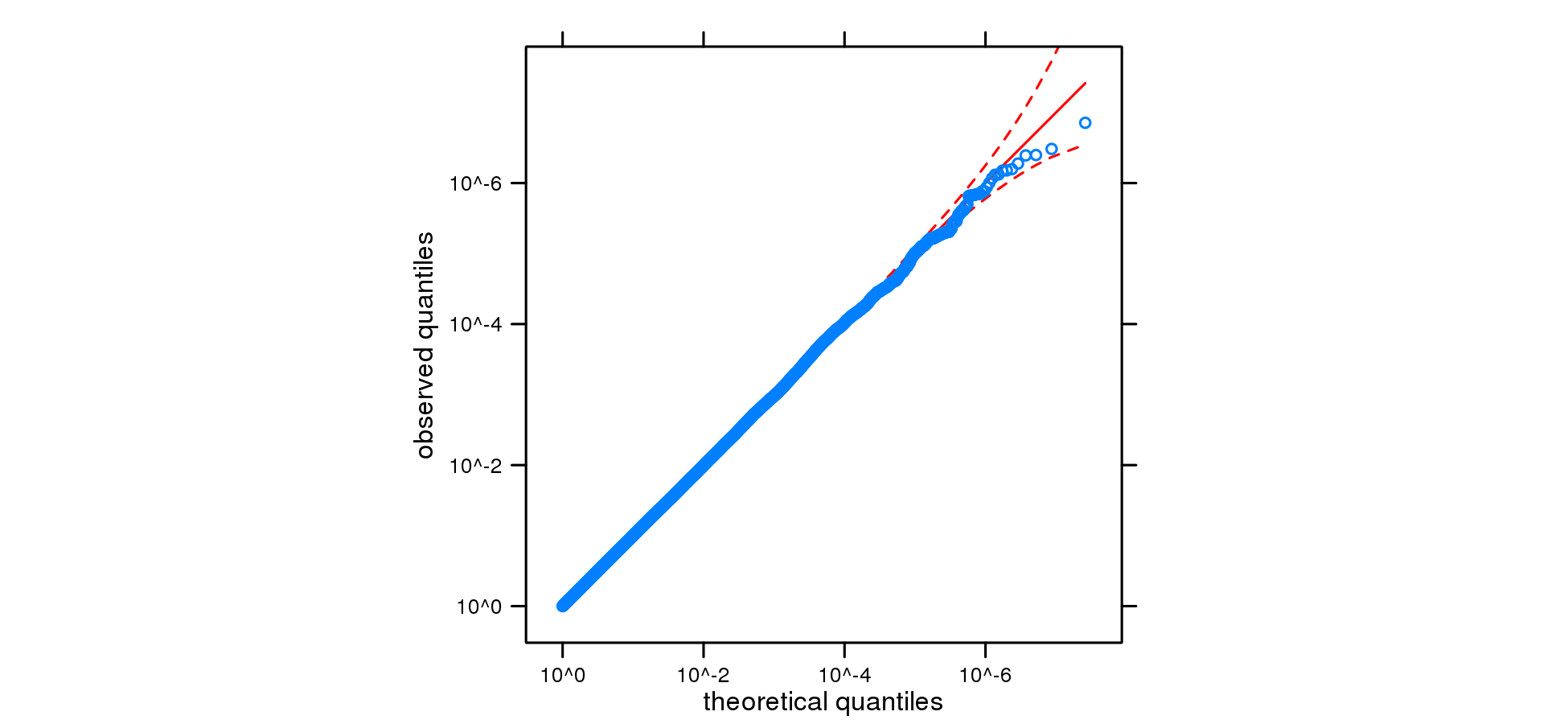


D


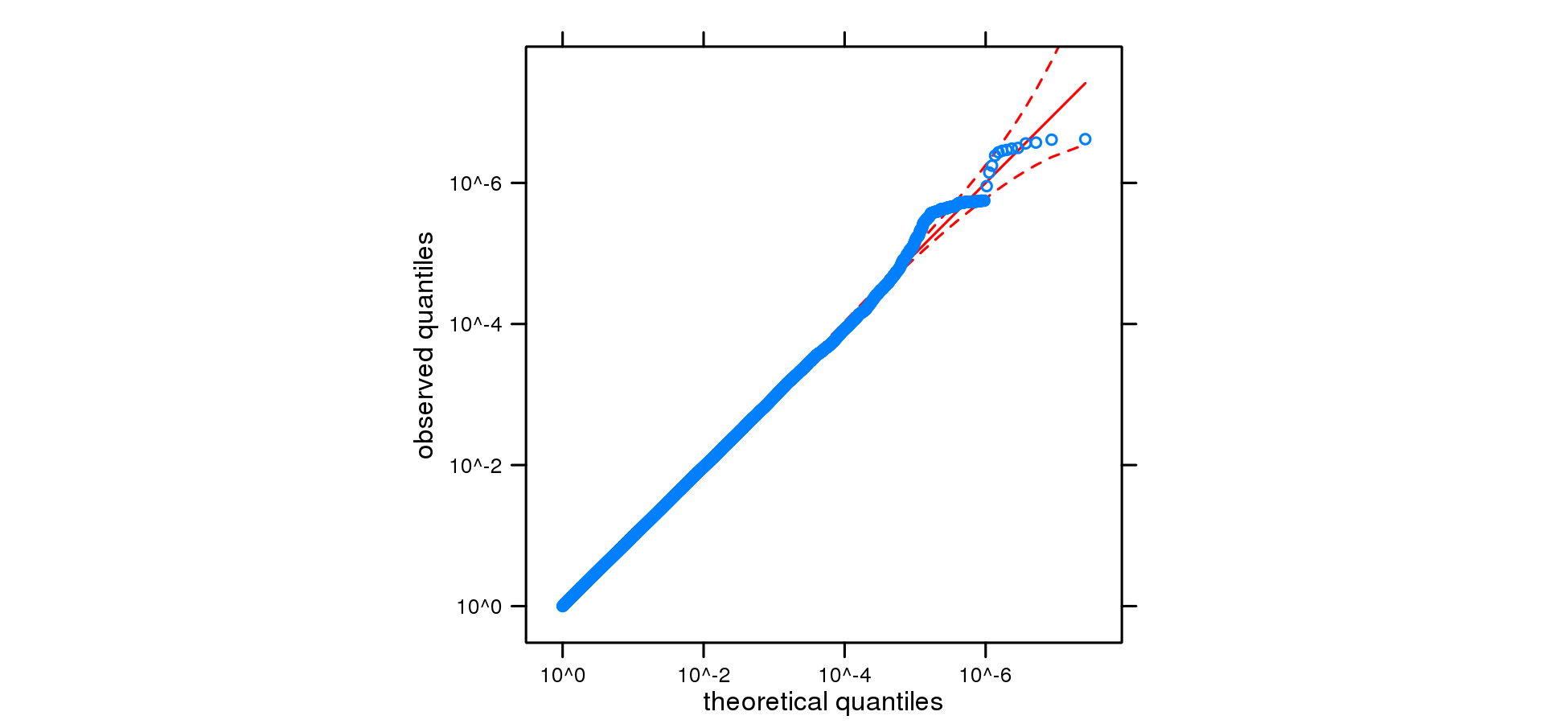


E


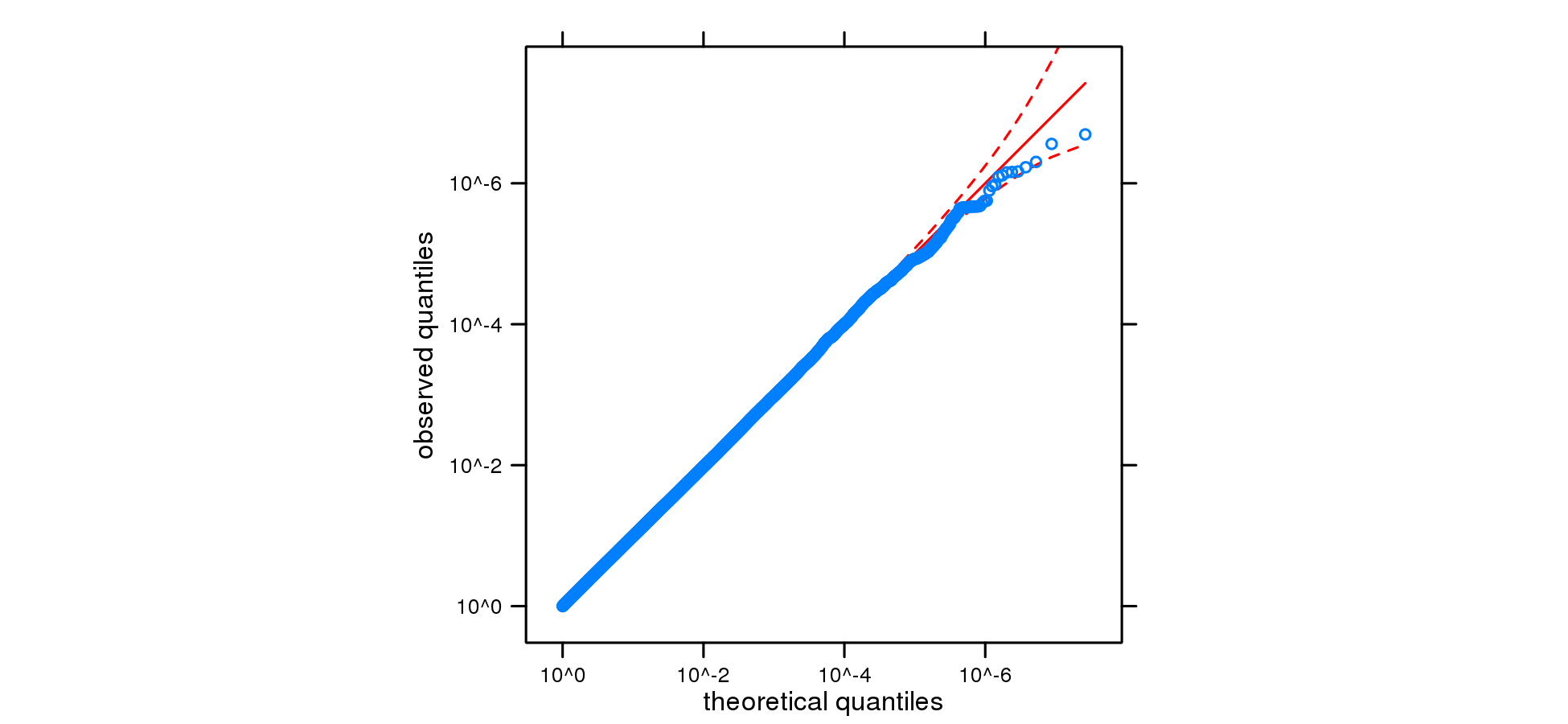


F


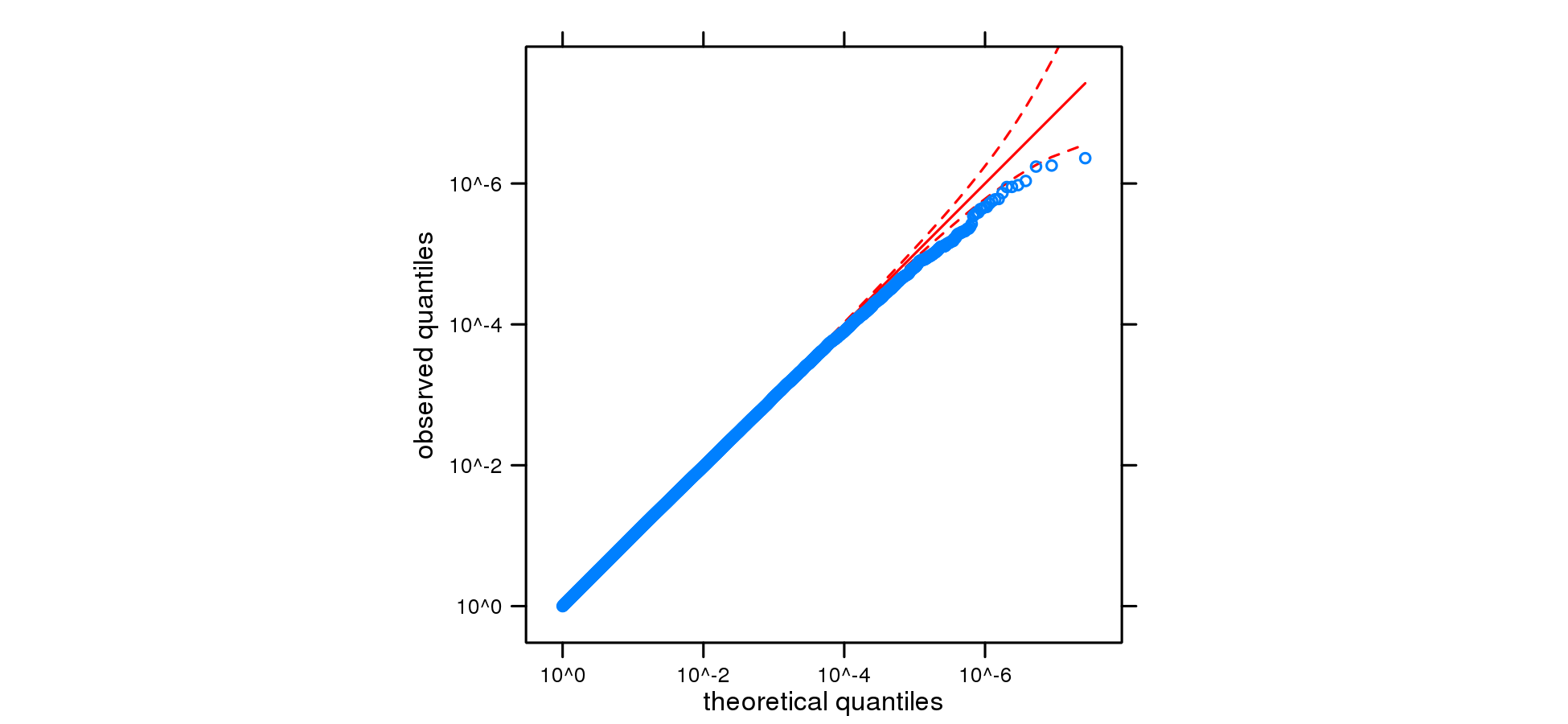


G


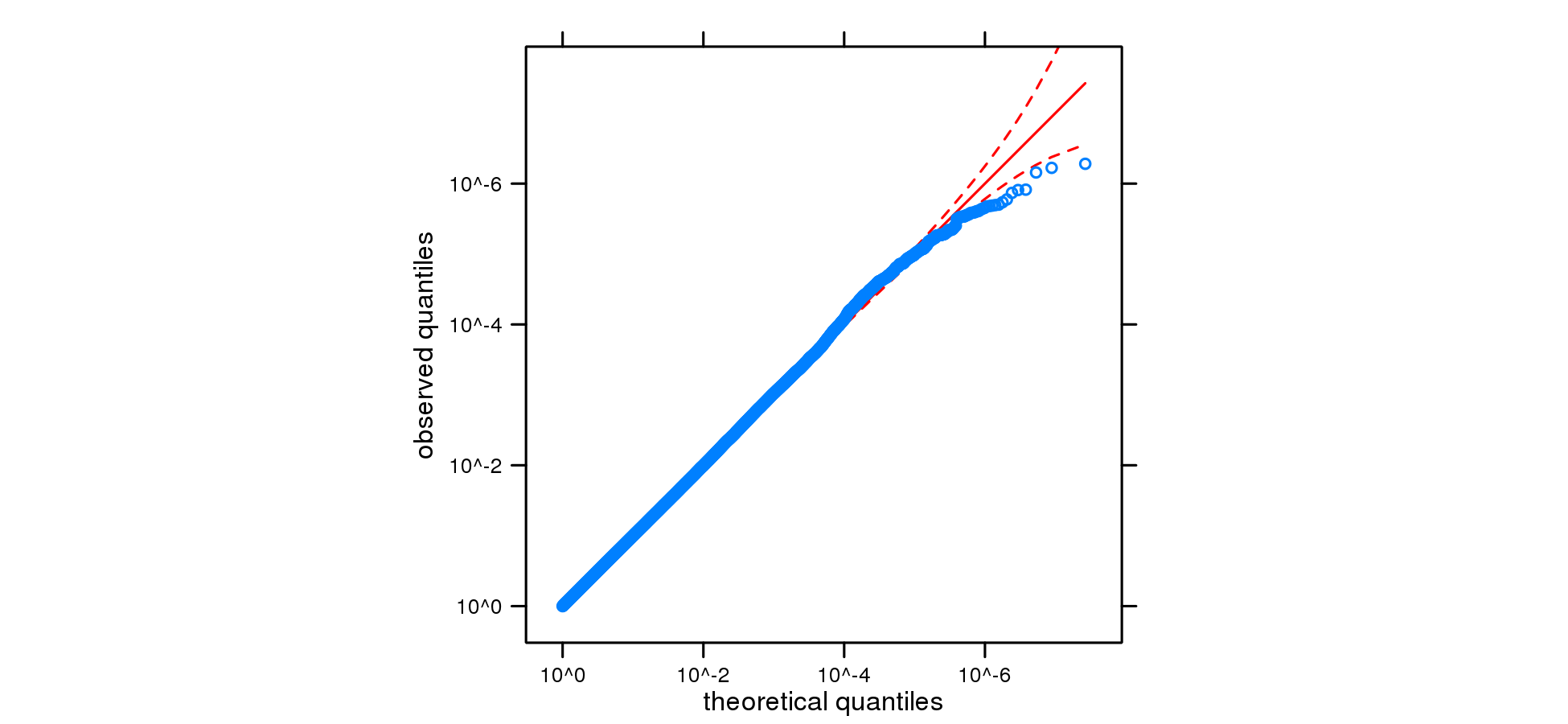


H


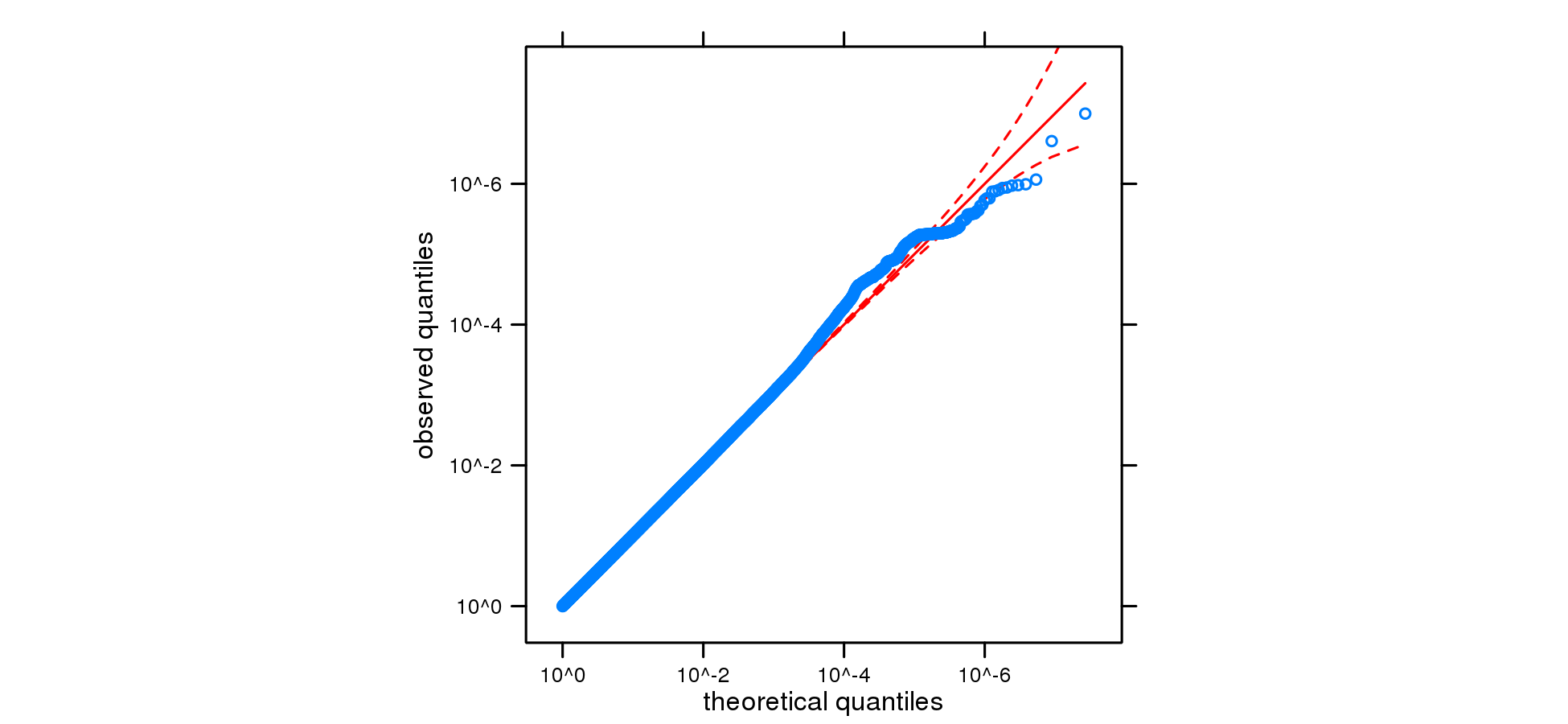


I


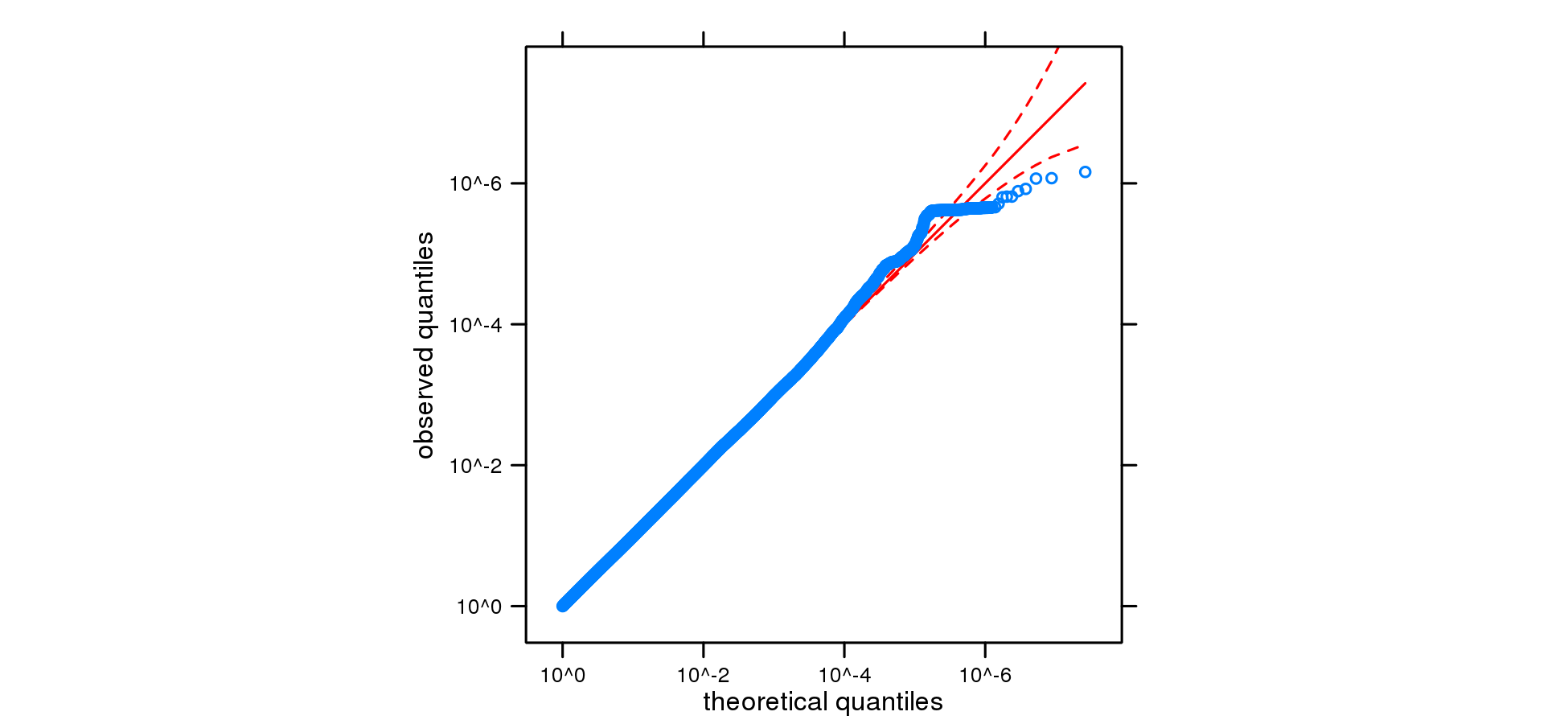


J


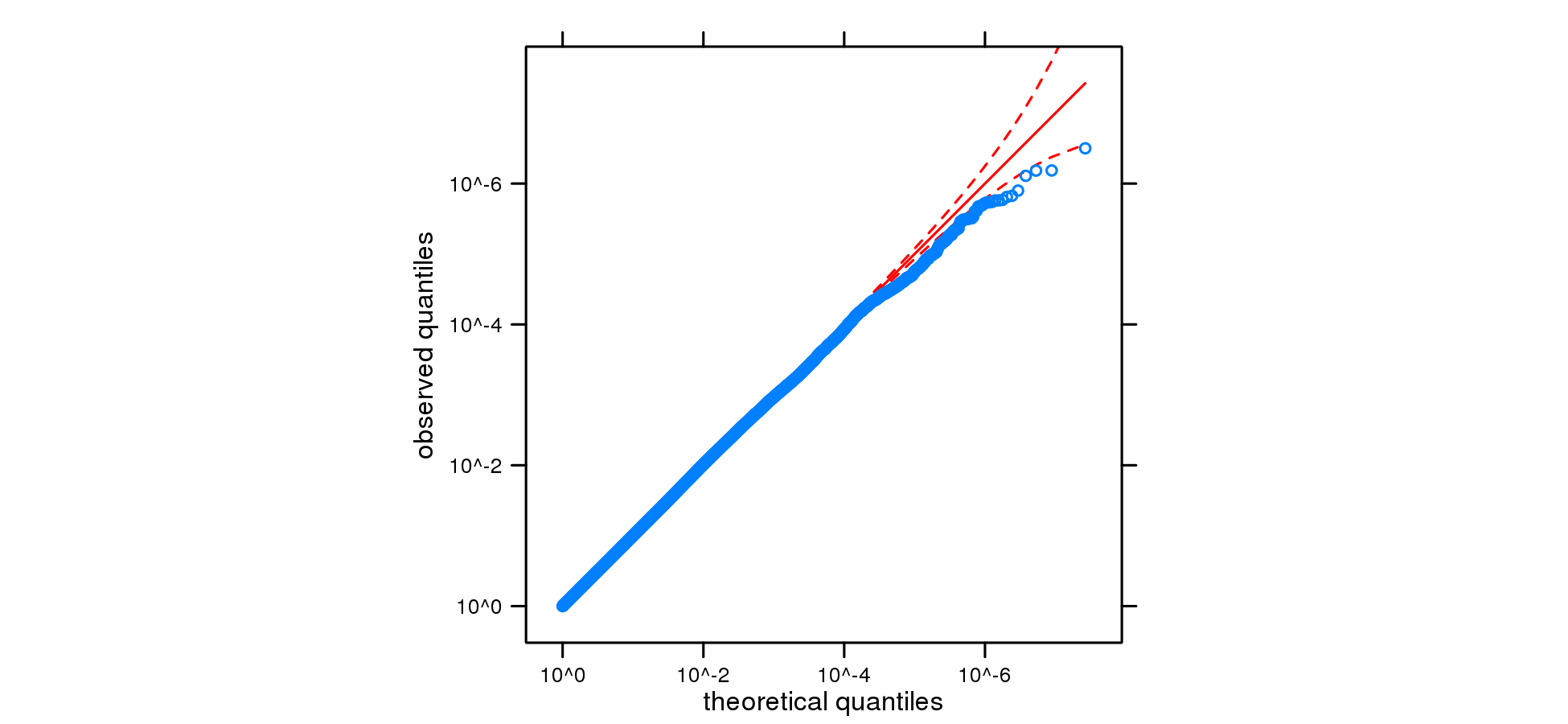


K


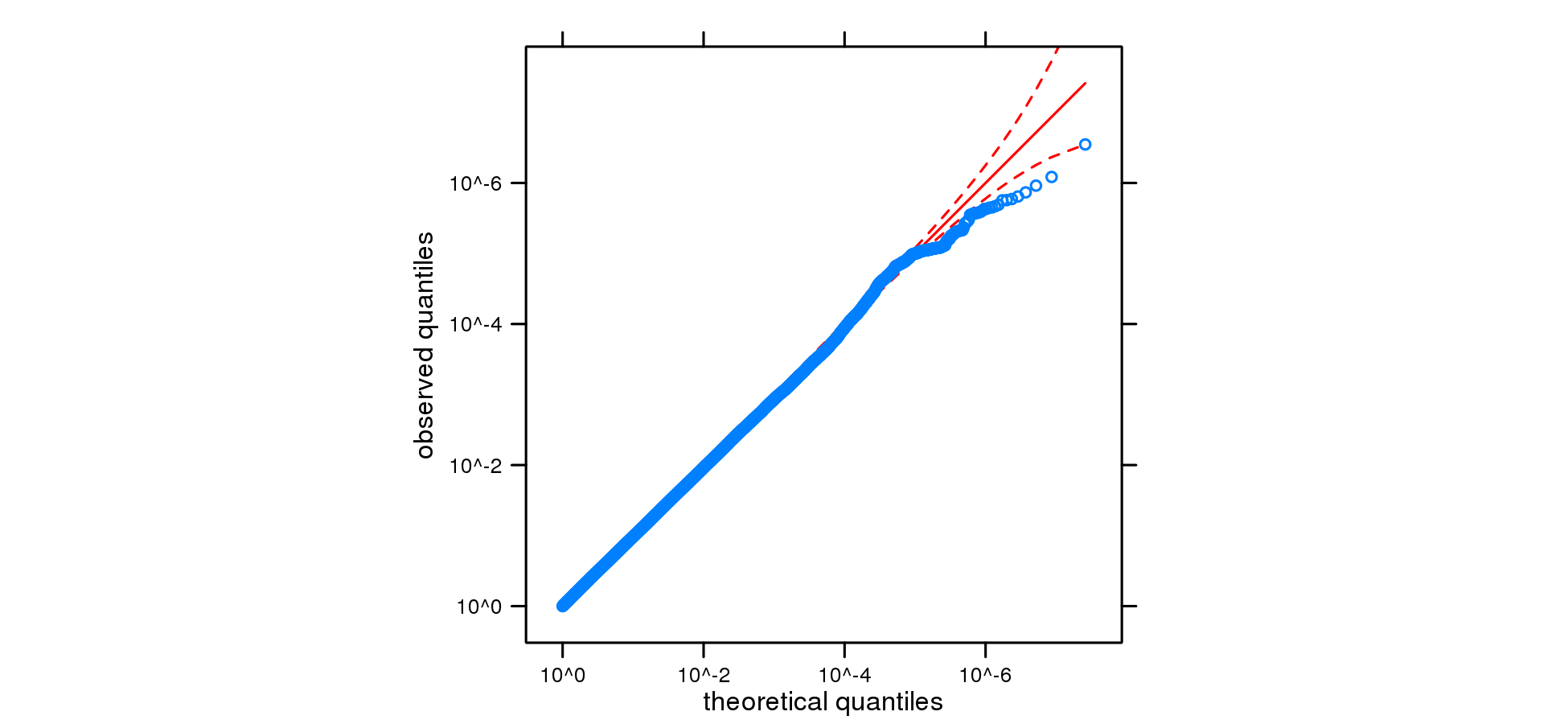


L


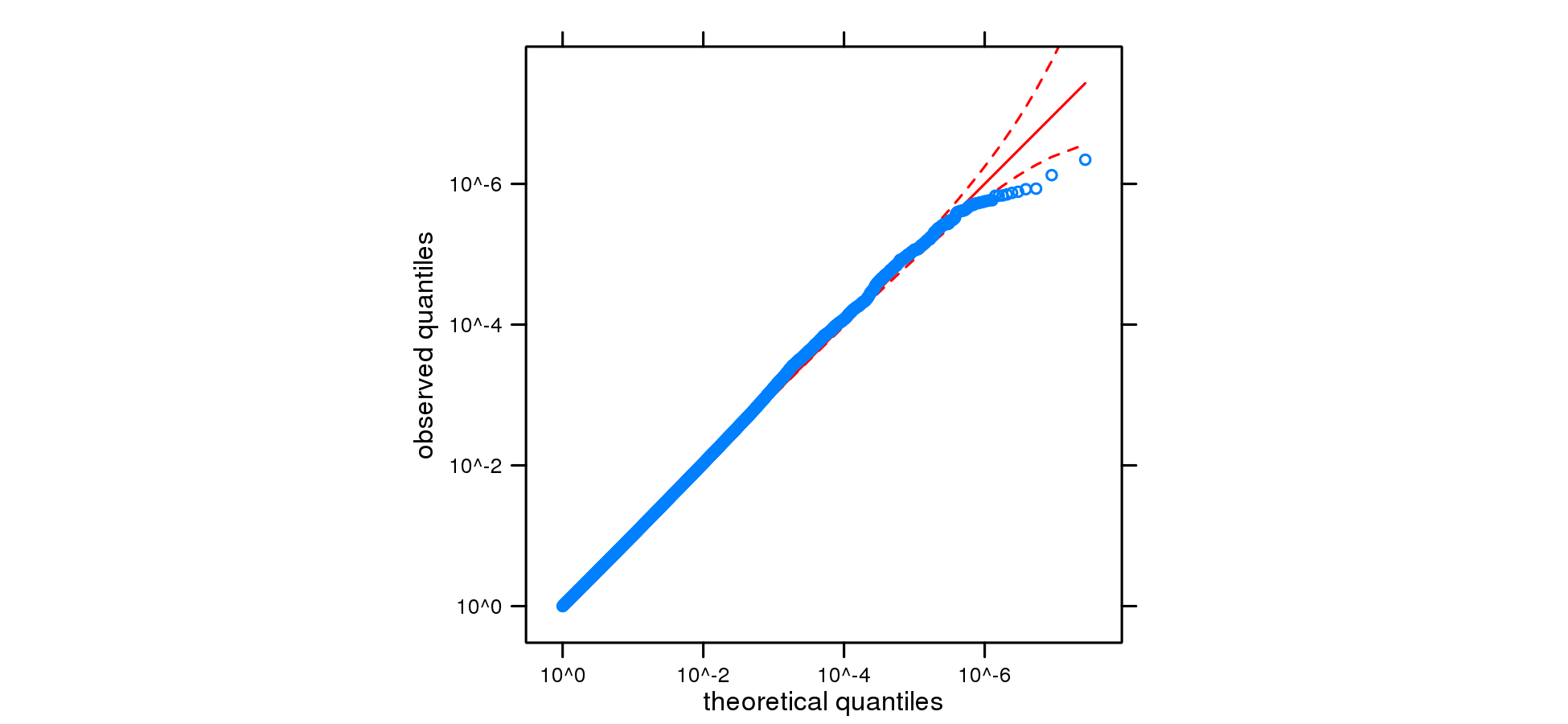


##### **Figure S3** Tissue gene expression pattern (A) FAM13A, (B) HERC5, (C) MMRN1, (D) CCSER1, (E) SNCA, and (F) GPRIN3 from GTEx portal. Data Source: GTEx Analysis Pilot V3 (dbGaP Accession phs000424.v3.p1)

# (A)


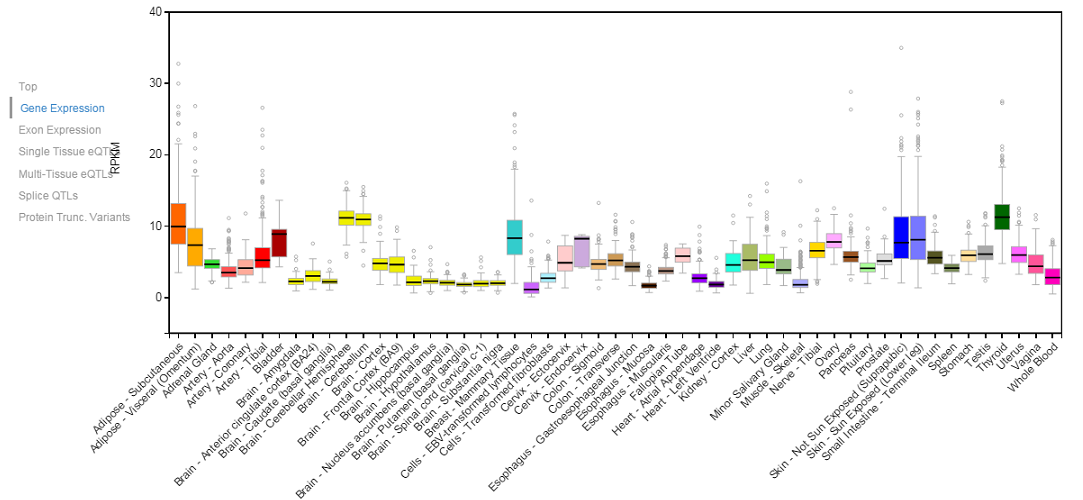


# (B)

**
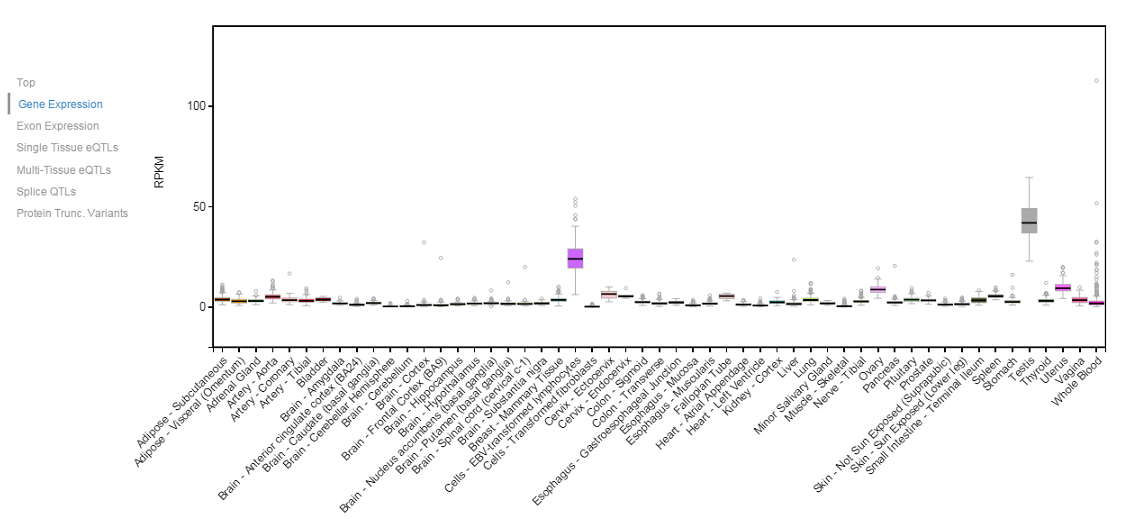
**

(C)**
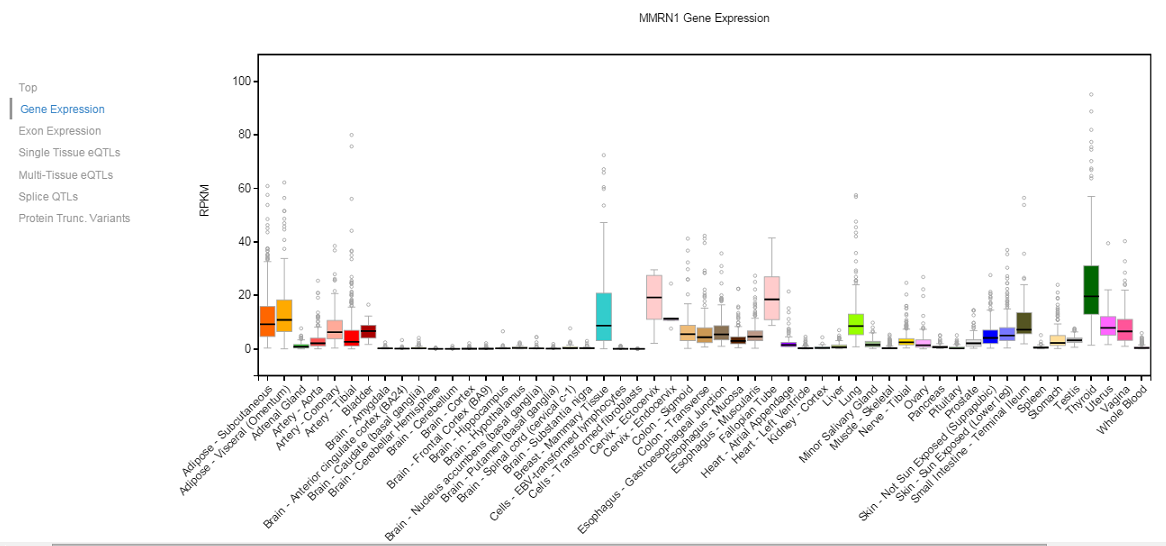
**

(D)

**
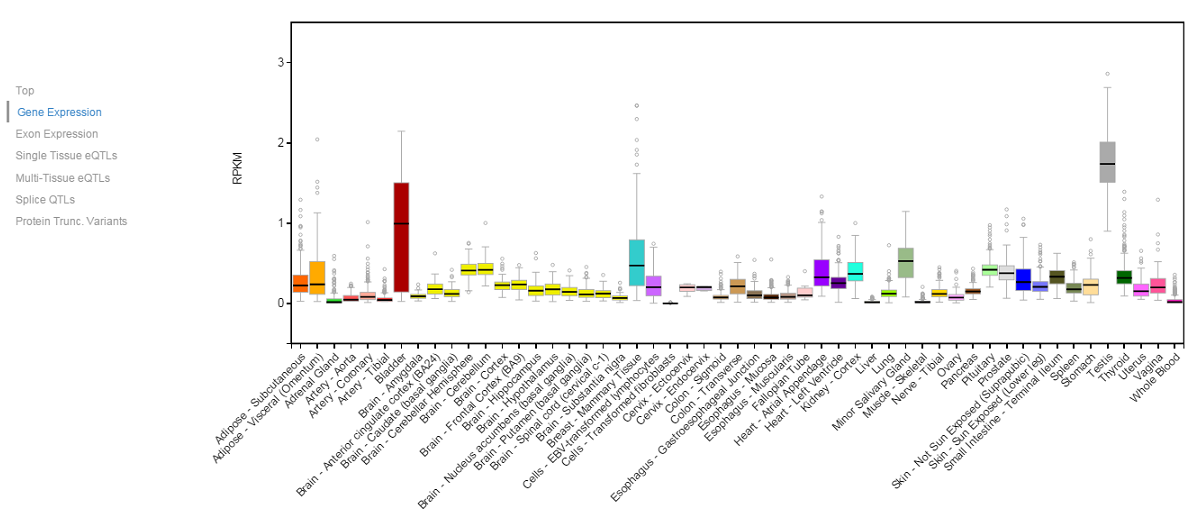
**

**(E)**

**
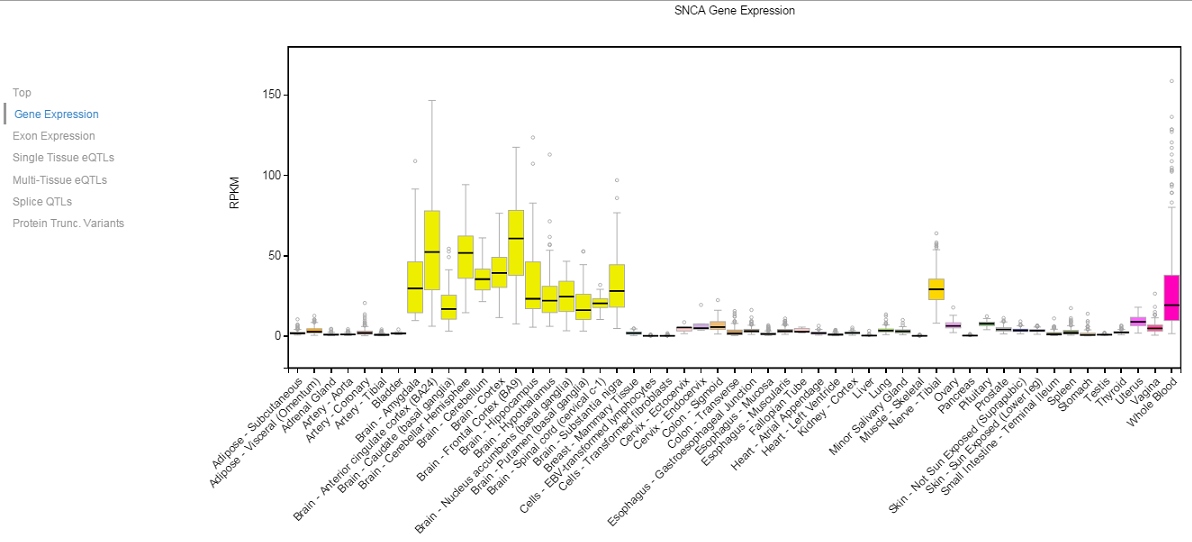
**

**(F)**

**
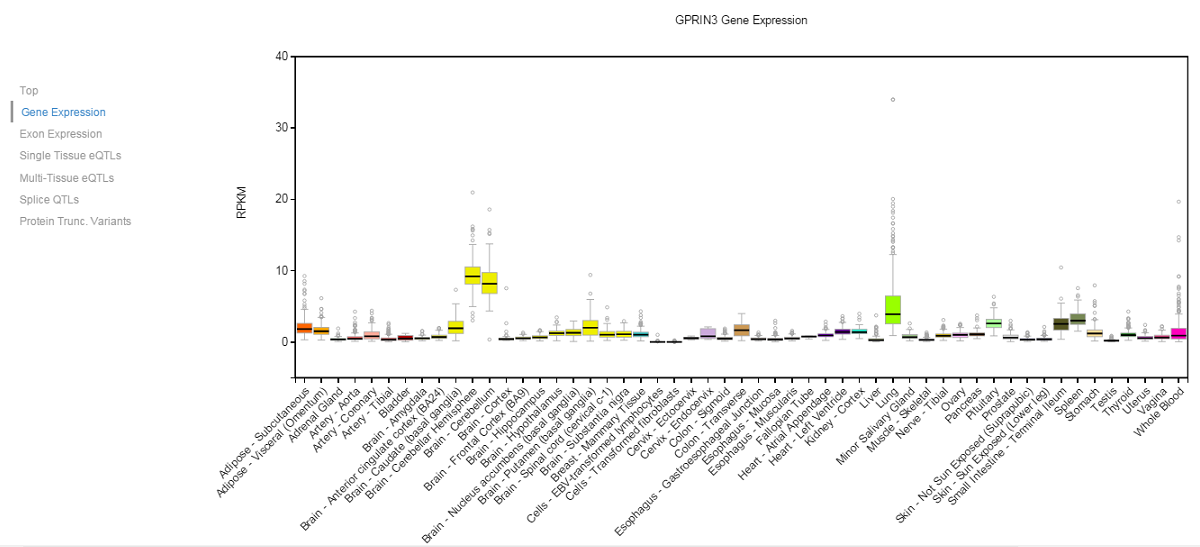
**

**Table S1** Sample size and basic demographic and genomic control inflation factor

| Group | Total | Gender | | Age | | | | Platform | | | Genomic control inflation factor | | |
| --- | --- | --- | --- | --- | --- | --- | --- | --- | --- | --- | --- | --- | --- |
|  |  | M | F | (0,30] | (30,45] | (45,60] | (60,Inf] | v1/v2 | v3 | v4 | λ | λ_1000_ | λ_10000_ |
| *NDRI responders (case) vs. non-responders (control)* | | | | | | | | | | | | | |
| case | 2675 | 799 | 1876 | 297 | 710 | 914 | 754 | 46 | 840 | 1789 |  |  |  |
| control | 1861 | 656 | 1205 | 300 | 569 | 541 | 451 | 27 | 663 | 1171 | 1.007 | 1.003 | 1.032 |
| *SSRI responders (case) vs. non-responders (control)* | | | | | | | | | | | | | |
| case | 6348 | 1770 | 4578 | 671 | 1543 | 2033 | 2101 | 103 | 1997 | 4248 |  |  |  |
| control | 3340 | 1229 | 2111 | 666 | 1106 | 863 | 705 | 44 | 1128 | 2168 | 1.008 | 1.002 | 1.018 |
| *citalopram or escitalopram responders (case) vs. non-responders (control)* | | | | | | | | | | | | | |
| case | 2963 | 811 | 2152 | 356 | 806 | 944 | 857 | 47 | 969 | 1947 |  |  |  |
| control | 2005 | 690 | 1315 | 438 | 672 | 516 | 379 | 22 | 661 | 1322 | 1.012 | 1.005 | 1.048 |
| *non-TRD (case) vs. TRD (control)* | | | | | | | | | | | | | |
| case | 7795 | 2204 | 5591 | 799 | 1834 | 2547 | 2615 | 122 | 2321 | 5352 |  |  |  |
| control | 1311 | 517 | 794 | 271 | 420 | 349 | 271 | 14 | 445 | 852 | 1.019 | 1.009 | 1.086 |
| *NDRI non-responder vs. healthy controls* | | | | | | | | | | | | | |
| case | 1861 | 656 | 1205 | 300 | 569 | 541 | 451 | 27 | 663 | 1171 |  |  |  |
| control | 192070 | 110498 | 81572 | 24592 | 50777 | 51106 | 65595 | 7111 | 97597 | 87362 | 1.024 | 1.007 | 1.066 |
| *NDRI responders vs. healthy controls* | | | | | | | | | | | | | |
| case | 2687 | 801 | 1886 | 299 | 713 | 917 | 758 | 46 | 842 | 1799 |  |  |  |
| control | 191986 | 110421 | 81565 | 24568 | 50761 | 51086 | 65571 | 7110 | 97581 | 87295 | 1.026 | 1.005 | 1.048 |
| *SSRI non-responders vs. healthy controls* | | | | | | | | | | | | | |
| case | 3340 | 1229 | 2111 | 666 | 1106 | 863 | 705 | 44 | 1128 | 2168 |  |  |  |
| control | 191882 | 110386 | 81496 | 24549 | 50756 | 51062 | 65515 | 7109 | 97492 | 87281 | 1.041 | 1.006 | 1.063 |
| *SSRI responders vs. healthy controls* | | | | | | | | | | | | | |
| case | 6387 | 1774 | 4613 | 678 | 1549 | 2040 | 2120 | 103 | 2008 | 4276 |  |  |  |
| control | 191357 | 110099 | 81258 | 24472 | 50649 | 50945 | 65291 | 7100 | 97332 | 86925 | 1.031 | 1.003 | 1.025 |
| *citalopram or escitalopram non-responders vs. healthy controls* | | | | | | | | | | | | | |
| case | 2005 | 690 | 1315 | 438 | 672 | 516 | 379 | 22 | 661 | 1322 |  |  |  |
| control | 192083 | 110488 | 81595 | 24577 | 50787 | 51114 | 65605 | 7112 | 97590 | 87381 | 1.034 | 1.009 | 1.086 |
| *citalopram or escitalopram responders vs. healthy controls* | | | | | | | | | | | | | |
| case | 2973 | 814 | 2159 | 357 | 808 | 945 | 863 | 47 | 972 | 1954 |  |  |  |
| control | 191936 | 110396 | 81540 | 24554 | 50751 | 51083 | 65548 | 7111 | 97538 | 87287 | 1.027 | 1.005 | 1.047 |
| *TRD vs. healthy controls* | | | | | | | | | | | | | |
| case | 1311 | 517 | 794 | 271 | 420 | 349 | 271 | 14 | 445 | 852 |  |  |  |
| control | 192178 | 110539 | 81639 | 24605 | 50800 | 51138 | 65635 | 7108 | 97638 | 87432 | 1.033 | 1.013 | 1.126 |
| *non-TRD vs. healthy controls* | | | | | | | | | | | | | |
| case | 7817 | 2204 | 5613 | 801 | 1836 | 2553 | 2627 | 122 | 2327 | 5368 |  |  |  |
| control | 191173 | 109986 | 81187 | 24433 | 50610 | 50907 | 65223 | 7096 | 97272 | 86805 | 1.043 | 1.003 | 1.029 |

Table S2 Genes accountable for gene set enrichment in bupropion responders vs. non-responders analysis

| Gene Loc | Gene ID | Gene _Desc | Target | P |
| --- | --- | --- | --- | --- |
| chr8:12803162..12887304 | 57604 | C8orf79 Chromosome_8_open_reading_frame_79 | chr8p22 Http://www.broadinstitute.org/gsea/msigdb/cards/chr8p22 | 1.00E-04 |
| chr8:13947352..15095812 | 137868 | SGCZ Sarcoglycan,_zeta | chr8p22 Http://www.broadinstitute.org/gsea/msigdb/cards/chr8p22 | 1.00E-04 |
| chr8:17501282..17658446 | 57509 | MTUS1 Microtubule_associated_tumor_suppressor_1 | chr8p22 Http://www.broadinstitute.org/gsea/msigdb/cards/chr8p22 | 1.00E-04 |
| chr8:17780345..17887477 | 5108 | PCM1 Pericentriolar_material_1 | chr8p22 Http://www.broadinstitute.org/gsea/msigdb/cards/chr8p22 | 1.00E-04 |
| chr8:19796561..19824790 | 4023 | LPL Lipoprotein_lipase | chr8p22 Http://www.broadinstitute.org/gsea/msigdb/cards/chr8p22 | 1.00E-04 |
| chr8:20103655..20112823 | 11178 | LZTS1 Leucine_zipper,_putative_tumor_suppressor_1 | chr8p22 Http://www.broadinstitute.org/gsea/msigdb/cards/chr8p22 | 1.00E-04 |
| chr8:22877627..22926720 | 8795 | TNFRSF10B Tumor_necrosis_factor_receptor_superfamily,_member_10b | chr8p22 Http://www.broadinstitute.org/gsea/msigdb/cards/chr8p22 | 1.00E-04 |
| chr8:125563007..125740750 | 9788 | MTSS1 Metastasis_suppressor_1 | chr8p22 Http://www.broadinstitute.org/gsea/msigdb/cards/chr8p22 | 1.00E-04 |
| chr1:108113761..108507565 | 10451 | VAV3 Vav_3_guanine_nucleotide_exchange_factor | GO:0001525 Angiogenesis | 0.00019998 |
| chr4:55944405..55991782 | 3791 | KDR Kinase_insert_domain_receptor_(a_type_III_receptor_tyrosine_kinase) | GO:0001525 Angiogenesis | 0.00019998 |
| chr4:74606254..74609453 | 3576 | IL8 Interleukin_8 | GO:0001525 Angiogenesis | 0.00019998 |
| chr4:75174183..75179327 | 255324 | EPGN Epithelial_mitogen_homolog_(mouse) | GO:0001525 Angiogenesis | 0.00019998 |
| chr4:75230839..75254497 | 2069 | EREG Epiregulin | GO:0001525 Angiogenesis | 0.00019998 |
| chr4:86396263..86923843 | 83478 | ARHGAP24 Rho_GTPase_activating_protein_24 | GO:0001525 Angiogenesis | 0.00019998 |
| chr7:15650816..15726328 | 4223 | MEOX2 Mesenchyme_homeobox_2 | GO:0001525 Angiogenesis | 0.00019998 |
| chr9:37915874..38069230 | 6461 | SHB Src_homology_2_domain_containing_adaptor_protein_B | GO:0001525 Angiogenesis | 0.00019998 |
| chr10:89623174..89728552 | 5728 | PTEN Phosphatase_and_tensin_homolog | GO:0001525 Angiogenesis | 0.00019998 |
| chr12:54789024..54813070 | 3678 | ITGA5 Integrin,_alpha_5_(fibronectin_receptor,_alpha_polypeptide) | GO:0001525 Angiogenesis | 0.00019998 |
| chr4:55095243..55164432 | 5156 | PDGFRA Platelet-derived_growth_factor_receptor,_alpha_polypeptide | GO:0005161 Platelet-derived growth factor receptor binding | 0.00019998 |
| chr10:89623174..89728552 | 5728 | PTEN Phosphatase_and_tensin_homolog | GO:0005161 Platelet-derived growth factor receptor binding | 0.00019998 |
| chr12:54789024..54813070 | 3678 | ITGA5 Integrin,_alpha_5_(fibronectin_receptor,_alpha_polypeptide) | GO:0005161 Platelet-derived growth factor receptor binding | 0.00019998 |
| chr6:21152505..21154724 | 100133941 | CD24 CD24_molecule | LIPID_HOMEOSTASIS Http://www.broadinstitute.org/gsea/msigdb/cards/LIPID_HOMEOSTASIS | 0.00019998 |
| chr9:107543263..107690547 | 19 | ABCA1 ATP-binding_cassette,_sub-family_A_(ABC1),_member_1 | LIPID_HOMEOSTASIS Http://www.broadinstitute.org/gsea/msigdb/cards/LIPID_HOMEOSTASIS | 0.00019998 |
| chr10:5005433..5020178 | 1645 | AKR1C1 Aldo-keto_reductase_family_1,_member_C1_(dihydrodiol_dehydrogenase_1;_20-alpha_(3-alpha)-hydroxysteroid_dehydrogenase) | LIPID_HOMEOSTASIS Http://www.broadinstitute.org/gsea/msigdb/cards/LIPID_HOMEOSTASIS | 0.00019998 |
| chr18:21111442..21166601 | 4864 | NPC1 Niemann-Pick_disease,_type_C1 | LIPID_HOMEOSTASIS Http://www.broadinstitute.org/gsea/msigdb/cards/LIPID_HOMEOSTASIS | 0.00019998 |
| chr19:45409018..45412670 | 348 | APOE Apolipoprotein_E | LIPID_HOMEOSTASIS Http://www.broadinstitute.org/gsea/msigdb/cards/LIPID_HOMEOSTASIS | 0.00019998 |
| chr1:6615317..6639837 | 80835 | TAS1R1 Taste_receptor,_type_1,_member_1 | SENSORY_PERCEPTION_OF_CHEMICAL_STIMULUS Http://www.broadinstitute.org/gsea/msigdb/cards/SENSORY_PERCEPTION_OF_CHEMICAL_STIMULUS | 0.00029997 |
| chr2:170683997..170940659 | 130507 | UBR3 Ubiquitin_protein_ligase_E3_component_n-recognin_3_(putative) | SENSORY_PERCEPTION_OF_CHEMICAL_STIMULUS Http://www.broadinstitute.org/gsea/msigdb/cards/SENSORY_PERCEPTION_OF_CHEMICAL_STIMULUS | 0.00029997 |
| chr4:20255214..20620808 | 9353 | SLIT2 Slit_homolog_2_(Drosophila) | SENSORY_PERCEPTION_OF_CHEMICAL_STIMULUS Http://www.broadinstitute.org/gsea/msigdb/cards/SENSORY_PERCEPTION_OF_CHEMICAL_STIMULUS | 0.00029997 |
| chr17:2965942..2966921 | 8386 | OR1D5 Olfactory_receptor,_family_1,_subfamily_D,_member_5 | SENSORY_PERCEPTION_OF_CHEMICAL_STIMULUS Http://www.broadinstitute.org/gsea/msigdb/cards/SENSORY_PERCEPTION_OF_CHEMICAL_STIMULUS | 0.00029997 |
| chr17:2995331..2996310 | 4991 | OR1D2 Olfactory_receptor,_family_1,_subfamily_D,_member_2 | SENSORY_PERCEPTION_OF_CHEMICAL_STIMULUS Http://www.broadinstitute.org/gsea/msigdb/cards/SENSORY_PERCEPTION_OF_CHEMICAL_STIMULUS | 0.00029997 |
| chr19:47813083..47825347 | 728 | C5AR1 Complement_component_5a_receptor_1 | SENSORY_PERCEPTION_OF_CHEMICAL_STIMULUS Http://www.broadinstitute.org/gsea/msigdb/cards/SENSORY_PERCEPTION_OF_CHEMICAL_STIMULUS | 0.00029997 |
| chr2:40339265..40739595 | 6546 | SLC8A1 Solute_carrier_family_8_(sodium/calcium_exchanger),_member_1 | REACTOME_PLATELET_CALCIUM_HOMEOSTASIS Http://www.broadinstitute.org/gsea/msigdb/cards/REACTOME_PLATELET_CALCIUM_HOMEOSTASIS | 0.00039996 |
| chr3:10365686..10547288 | 491 | ATP2B2 ATPase,_Ca++_transporting,_plasma_membrane_2 | REACTOME_PLATELET_CALCIUM_HOMEOSTASIS Http://www.broadinstitute.org/gsea/msigdb/cards/REACTOME_PLATELET_CALCIUM_HOMEOSTASIS | 0.00039996 |
| chr6:33589135..33664368 | 3710 | ITPR3 Inositol_1,4,5-triphosphate_receptor,_type_3 | REACTOME_PLATELET_CALCIUM_HOMEOSTASIS Http://www.broadinstitute.org/gsea/msigdb/cards/REACTOME_PLATELET_CALCIUM_HOMEOSTASIS | 0.00039996 |
| chr11:3876912..4114460 | 6786 | STIM1 Stromal_interaction_molecule_1 | REACTOME_PLATELET_CALCIUM_HOMEOSTASIS Http://www.broadinstitute.org/gsea/msigdb/cards/REACTOME_PLATELET_CALCIUM_HOMEOSTASIS | 0.00039996 |
| chr12:26488264..26986151 | 3709 | ITPR2 Inositol_1,4,5-triphosphate_receptor,_type_2 | REACTOME_PLATELET_CALCIUM_HOMEOSTASIS Http://www.broadinstitute.org/gsea/msigdb/cards/REACTOME_PLATELET_CALCIUM_HOMEOSTASIS | 0.00039996 |
| chr17:3799864..3819980 | 5023 | P2RX1 Purinergic_receptor_P2X,_ligand-gated_ion_channel,_1 | REACTOME_PLATELET_CALCIUM_HOMEOSTASIS Http://www.broadinstitute.org/gsea/msigdb/cards/REACTOME_PLATELET_CALCIUM_HOMEOSTASIS | 0.00039996 |
| chr17:3827148..3867756 | 489 | ATP2A3 ATPase,_Ca++_transporting,_ubiquitous | REACTOME_PLATELET_CALCIUM_HOMEOSTASIS Http://www.broadinstitute.org/gsea/msigdb/cards/REACTOME_PLATELET_CALCIUM_HOMEOSTASIS | 0.00039996 |
| chr19:47931258..47975327 | 6543 | SLC8A2 Solute_carrier_family_8_(sodium/calcium_exchanger),_member_2 | REACTOME_PLATELET_CALCIUM_HOMEOSTASIS Http://www.broadinstitute.org/gsea/msigdb/cards/REACTOME_PLATELET_CALCIUM_HOMEOSTASIS | 0.00039996 |
| chr4:93225529..94693669 | 2895 | GRID2 Glutamate_receptor,_ionotropic,_delta_2 | KEGG_LONG_TERM_DEPRESSION Http://www.broadinstitute.org/gsea/msigdb/cards/KEGG_LONG_TERM_DEPRESSION | 0.00049995 |
| chr6:146348761..146758751 | 2911 | GRM1 Glutamate_receptor,_metabotropic_1 | KEGG_LONG_TERM_DEPRESSION Http://www.broadinstitute.org/gsea/msigdb/cards/KEGG_LONG_TERM_DEPRESSION | 0.00049995 |
| chr11:88237723..88796836 | 2915 | GRM5 Glutamate_receptor,_metabotropic_5 | KEGG_LONG_TERM_DEPRESSION Http://www.broadinstitute.org/gsea/msigdb/cards/KEGG_LONG_TERM_DEPRESSION | 0.00049995 |
| chr17:954294..43913214 | 1394 | CRHR1 Corticotropin_releasing_hormone_receptor_1 | KEGG_LONG_TERM_DEPRESSION Http://www.broadinstitute.org/gsea/msigdb/cards/KEGG_LONG_TERM_DEPRESSION | 0.00049995 |
| chr17:64298905..64806882 | 5578 | PRKCA Protein_kinase_C,_alpha | KEGG_LONG_TERM_DEPRESSION Http://www.broadinstitute.org/gsea/msigdb/cards/KEGG_LONG_TERM_DEPRESSION | 0.00049995 |
| chr1:108113761..108507565 | 10451 | VAV3 Vav_3_guanine_nucleotide_exchange_factor | PID_CDC42_REG_PATHWAY Http://www.broadinstitute.org/gsea/msigdb/cards/PID_CDC42_REG_PATHWAY | 0.00049995 |
| chr20:39766140..39804377 | 5335 | PLCG1 Phospholipase_C,_gamma_1 | PID_CDC42_REG_PATHWAY Http://www.broadinstitute.org/gsea/msigdb/cards/PID_CDC42_REG_PATHWAY | 0.00049995 |
| chr21:35014763..35261629 | 6453 | ITSN1 Intersectin_1_(SH3_domain_protein) | PID_CDC42_REG_PATHWAY Http://www.broadinstitute.org/gsea/msigdb/cards/PID_CDC42_REG_PATHWAY | 0.00049995 |
| chr4:93225529..94693669 | 2895 | GRID2 Glutamate_receptor,_ionotropic,_delta_2 | 04730 Long-term_depression | 0.00059994 |
| chr6:146348761..146758751 | 2911 | GRM1 Glutamate_receptor,_metabotropic_1 | 04730 Long-term_depression | 0.00059994 |
| chr11:88237723..88796836 | 2915 | GRM5 Glutamate_receptor,_metabotropic_5 | 04730 Long-term_depression | 0.00059994 |
| chr17:954294..43913214 | 1394 | CRHR1 Corticotropin_releasing_hormone_receptor_1 | 04730 Long-term_depression | 0.00059994 |
| chr17:64298905..64806882 | 5578 | PRKCA Protein_kinase_C,_alpha | 04730 Long-term_depression | 0.00059994 |
| chr1:108113761..108507565 | 10451 | VAV3 Vav_3_guanine_nucleotide_exchange_factor | SH3_SH2_ADAPTOR_ACTIVITY Http://www.broadinstitute.org/gsea/msigdb/cards/SH3_SH2_ADAPTOR_ACTIVITY | 0.00069993 |
| chr7:29234100..29553964 | 1124 | CHN2 Chimerin_(chimaerin)_2 | SH3_SH2_ADAPTOR_ACTIVITY Http://www.broadinstitute.org/gsea/msigdb/cards/SH3_SH2_ADAPTOR_ACTIVITY | 0.00069993 |
| chr9:37915874..38069230 | 6461 | SHB Src_homology_2_domain_containing_adaptor_protein_B | SH3_SH2_ADAPTOR_ACTIVITY Http://www.broadinstitute.org/gsea/msigdb/cards/SH3_SH2_ADAPTOR_ACTIVITY | 0.00069993 |
| chr1:223566694..223568832 | 164127 | C1orf65 Chromosome_1_open_reading_frame_65 | chr1q42 Http://www.broadinstitute.org/gsea/msigdb/cards/chr1q42 | 0.00079992 |
| chr1:226819370..226926896 | 3707 | ITPKB Inositol_1,4,5-trisphosphate_3-kinase_B | chr1q42 Http://www.broadinstitute.org/gsea/msigdb/cards/chr1q42 | 0.00079992 |
| chr1:228870848..228882431 | 58480 | RHOU Ras_homolog_gene_family,_member_U | chr1q42 Http://www.broadinstitute.org/gsea/msigdb/cards/chr1q42 | 0.00079992 |
| chr1:233749729..233808278 | 3775 | KCNK1 Potassium_channel,_subfamily_K,_member_1 | chr1q42 Http://www.broadinstitute.org/gsea/msigdb/cards/chr1q42 | 0.00079992 |
| chr1:237205681..237997308 | 6262 | RYR2 Ryanodine_receptor_2_(cardiac) | chr1q42 Http://www.broadinstitute.org/gsea/msigdb/cards/chr1q42 | 0.00079992 |
| chr17:4172491..4269989 | 7326 | UBE2G1 Ubiquitin-conjugating_enzyme_E2G_1_(UBC7_homolog,_yeast) | chr1q42 Http://www.broadinstitute.org/gsea/msigdb/cards/chr1q42 | 0.00079992 |
| chr5:180028485..180076644 | 2324 | FLT4 Fms-related_tyrosine_kinase_4 | BIOCARTA_VEGF_PATHWAY Http://www.broadinstitute.org/gsea/msigdb/cards/BIOCARTA_VEGF_PATHWAY | 0.00089991 |
| chr17:64298905..64806882 | 5578 | PRKCA Protein_kinase_C,_alpha | BIOCARTA_VEGF_PATHWAY Http://www.broadinstitute.org/gsea/msigdb/cards/BIOCARTA_VEGF_PATHWAY | 0.00089991 |
| chr20:39766140..39804377 | 5335 | PLCG1 Phospholipase_C,_gamma_1 | BIOCARTA_VEGF_PATHWAY Http://www.broadinstitute.org/gsea/msigdb/cards/BIOCARTA_VEGF_PATHWAY | 0.00089991 |
| chr1:7844742..7905257 | 8863 | PER3 Period_homolog_3_(Drosophila) | 04710 Circadian_rhythm_-_mammal | 0.00119988 |
| chr15:60780462..61521522 | 6095 | RORA RAR-related_orphan_receptor_A | 04710 Circadian_rhythm_-_mammal | 0.00119988 |
| chr17:38249016..38256993 | 9572 | NR1D1 Nuclear_receptor_subfamily_1,_group_D,_member_1 | 04710 Circadian_rhythm_-_mammal | 0.00119988 |
| chr1:108113761..108507565 | 10451 | VAV3 Vav_3_guanine_nucleotide_exchange_factor | MOLECULAR_ADAPTOR_ACTIVITY Http://www.broadinstitute.org/gsea/msigdb/cards/MOLECULAR_ADAPTOR_ACTIVITY | 0.00119988 |
| chr7:29234100..29553964 | 1124 | CHN2 Chimerin_(chimaerin)_2 | MOLECULAR_ADAPTOR_ACTIVITY Http://www.broadinstitute.org/gsea/msigdb/cards/MOLECULAR_ADAPTOR_ACTIVITY | 0.00119988 |
| chr9:37915874..38069230 | 6461 | SHB Src_homology_2_domain_containing_adaptor_protein_B | MOLECULAR_ADAPTOR_ACTIVITY Http://www.broadinstitute.org/gsea/msigdb/cards/MOLECULAR_ADAPTOR_ACTIVITY | 0.00119988 |
| chr1:6845363..7829786 | 23261 | CAMTA1 Calmodulin_binding_transcription_activator_1 | GGCCAGT,MIR-193A,MIR-193B Http://www.broadinstitute.org/gsea/msigdb/cards/GGCCAGT,MIR-193A,MIR-193B | 0.00129987 |
| chr4:55524074..55606901 | 3815 | KIT V-kit_Hardy-Zuckerman_4_feline_sarcoma_viral_oncogene_homolog | GGCCAGT,MIR-193A,MIR-193B Http://www.broadinstitute.org/gsea/msigdb/cards/GGCCAGT,MIR-193A,MIR-193B | 0.00129987 |
| chr10:89623174..89728552 | 5728 | PTEN Phosphatase_and_tensin_homolog | GGCCAGT,MIR-193A,MIR-193B Http://www.broadinstitute.org/gsea/msigdb/cards/GGCCAGT,MIR-193A,MIR-193B | 0.00129987 |
| chr12:11802767..12048345 | 2120 | ETV6 Ets_variant_6 | GGCCAGT,MIR-193A,MIR-193B Http://www.broadinstitute.org/gsea/msigdb/cards/GGCCAGT,MIR-193A,MIR-193B | 0.00129987 |
| chr20:39807068..39928759 | 23051 | ZHX3 Zinc_fingers_and_homeoboxes_3 | GGCCAGT,MIR-193A,MIR-193B Http://www.broadinstitute.org/gsea/msigdb/cards/GGCCAGT,MIR-193A,MIR-193B | 0.00129987 |
| chr1:108113761..108507565 | 10451 | VAV3 Vav_3_guanine_nucleotide_exchange_factor | PROTEIN_BINDING_BRIDGING Http://www.broadinstitute.org/gsea/msigdb/cards/PROTEIN_BINDING_BRIDGING | 0.00129987 |
| chr6:7541849..7586966 | 1832 | DSP Desmoplakin | PROTEIN_BINDING_BRIDGING Http://www.broadinstitute.org/gsea/msigdb/cards/PROTEIN_BINDING_BRIDGING | 0.00129987 |
| chr7:29234100..29553964 | 1124 | CHN2 Chimerin_(chimaerin)_2 | PROTEIN_BINDING_BRIDGING Http://www.broadinstitute.org/gsea/msigdb/cards/PROTEIN_BINDING_BRIDGING | 0.00129987 |
| chr9:37915874..38069230 | 6461 | SHB Src_homology_2_domain_containing_adaptor_protein_B | PROTEIN_BINDING_BRIDGING Http://www.broadinstitute.org/gsea/msigdb/cards/PROTEIN_BINDING_BRIDGING | 0.00129987 |
| chr17:37026091..37078043 | 3927 | LASP1 LIM_and_SH3_protein_1 | PROTEIN_BINDING_BRIDGING Http://www.broadinstitute.org/gsea/msigdb/cards/PROTEIN_BINDING_BRIDGING | 0.00129987 |
| chr17:37894166..37903558 | 2886 | GRB7 Growth_factor_receptor-bound_protein_7 | PROTEIN_BINDING_BRIDGING Http://www.broadinstitute.org/gsea/msigdb/cards/PROTEIN_BINDING_BRIDGING | 0.00129987 |
| chr1:108113761..108507565 | 10451 | VAV3 Vav_3_guanine_nucleotide_exchange_factor | 04666 Fc_gamma_R-mediated_phagocytosis | 0.00189981 |
| chr9:124030359..124095140 | 2934 | GSN Gelsolin | 04666 Fc_gamma_R-mediated_phagocytosis | 0.00189981 |
| chr17:64298905..64806882 | 5578 | PRKCA Protein_kinase_C,_alpha | 04666 Fc_gamma_R-mediated_phagocytosis | 0.00189981 |
| chr20:39766140..39804377 | 5335 | PLCG1 Phospholipase_C,_gamma_1 | 04666 Fc_gamma_R-mediated_phagocytosis | 0.00189981 |
| chr1:108113761..108507565 | 10451 | VAV3 Vav_3_guanine_nucleotide_exchange_factor | KEGG_FC_GAMMA_R_MEDIATED_PHAGOCYTOSIS Http://www.broadinstitute.org/gsea/msigdb/cards/KEGG_FC_GAMMA_R_MEDIATED_PHAGOCYTOSIS | 0.00189981 |
| chr9:124030359..124095140 | 2934 | GSN Gelsolin | KEGG_FC_GAMMA_R_MEDIATED_PHAGOCYTOSIS Http://www.broadinstitute.org/gsea/msigdb/cards/KEGG_FC_GAMMA_R_MEDIATED_PHAGOCYTOSIS | 0.00189981 |
| chr17:64298905..64806882 | 5578 | PRKCA Protein_kinase_C,_alpha | KEGG_FC_GAMMA_R_MEDIATED_PHAGOCYTOSIS Http://www.broadinstitute.org/gsea/msigdb/cards/KEGG_FC_GAMMA_R_MEDIATED_PHAGOCYTOSIS | 0.00189981 |
| chr20:39766140..39804377 | 5335 | PLCG1 Phospholipase_C,_gamma_1 | KEGG_FC_GAMMA_R_MEDIATED_PHAGOCYTOSIS Http://www.broadinstitute.org/gsea/msigdb/cards/KEGG_FC_GAMMA_R_MEDIATED_PHAGOCYTOSIS | 0.00189981 |
| chr1:108113761..108507565 | 10451 | VAV3 Vav_3_guanine_nucleotide_exchange_factor | REACTOME_SIGNALING_BY_RHO_GTPASES Http://www.broadinstitute.org/gsea/msigdb/cards/REACTOME_SIGNALING_BY_RHO_GTPASES | 0.00219978 |
| chr7:29234100..29553964 | 1124 | CHN2 Chimerin_(chimaerin)_2 | REACTOME_SIGNALING_BY_RHO_GTPASES Http://www.broadinstitute.org/gsea/msigdb/cards/REACTOME_SIGNALING_BY_RHO_GTPASES | 0.00219978 |
| chr20:37230556..37279315 | 343578 | ARHGAP40 Rho_GTPase_activating_protein_40 | REACTOME_SIGNALING_BY_RHO_GTPASES Http://www.broadinstitute.org/gsea/msigdb/cards/REACTOME_SIGNALING_BY_RHO_GTPASES | 0.00219978 |
| chr21:35014763..35261629 | 6453 | ITSN1 Intersectin_1_(SH3_domain_protein) | REACTOME_SIGNALING_BY_RHO_GTPASES Http://www.broadinstitute.org/gsea/msigdb/cards/REACTOME_SIGNALING_BY_RHO_GTPASES | 0.00219978 |
| chr10:89623174..89728552 | 5728 | PTEN Phosphatase_and_tensin_homolog | KEGG_PHOSPHATIDYLINOSITOL_SIGNALING_SYSTEM Http://www.broadinstitute.org/gsea/msigdb/cards/KEGG_PHOSPHATIDYLINOSITOL_SIGNALING_SYSTEM | 0.00269973 |
| chr17:64298905..64806882 | 5578 | PRKCA Protein_kinase_C,_alpha | KEGG_PHOSPHATIDYLINOSITOL_SIGNALING_SYSTEM Http://www.broadinstitute.org/gsea/msigdb/cards/KEGG_PHOSPHATIDYLINOSITOL_SIGNALING_SYSTEM | 0.00269973 |
| chr20:39766140..39804377 | 5335 | PLCG1 Phospholipase_C,_gamma_1 | KEGG_PHOSPHATIDYLINOSITOL_SIGNALING_SYSTEM Http://www.broadinstitute.org/gsea/msigdb/cards/KEGG_PHOSPHATIDYLINOSITOL_SIGNALING_SYSTEM | 0.00269973 |
| chr6:7107809..7252233 | 6239 | RREB1 Ras_responsive_element_binding_protein_1 | HALLMARK_ADIPOGENESIS Http://www.broadinstitute.org/gsea/msigdb/cards/HALLMARK_ADIPOGENESIS | 0.00279972 |
| chr9:124101332..124132565 | 2040 | STOM Stomatin | HALLMARK_ADIPOGENESIS Http://www.broadinstitute.org/gsea/msigdb/cards/HALLMARK_ADIPOGENESIS | 0.00279972 |
| chr12:112204325..112247804 | 217 | ALDH2 Aldehyde_dehydrogenase_2_family_(mitochondrial) | HALLMARK_ADIPOGENESIS Http://www.broadinstitute.org/gsea/msigdb/cards/HALLMARK_ADIPOGENESIS | 0.00279972 |
| chr12:125262153..125348539 | 949 | SCARB1 Scavenger_receptor_class_B,_member_1 | HALLMARK_ADIPOGENESIS Http://www.broadinstitute.org/gsea/msigdb/cards/HALLMARK_ADIPOGENESIS | 0.00279972 |
| chr20:37209817..37217124 | 149685 | ADIG Adipogenin | HALLMARK_ADIPOGENESIS Http://www.broadinstitute.org/gsea/msigdb/cards/HALLMARK_ADIPOGENESIS | 0.00279972 |
| chr21:35014763..35261629 | 6453 | ITSN1 Intersectin_1_(SH3_domain_protein) | HALLMARK_ADIPOGENESIS Http://www.broadinstitute.org/gsea/msigdb/cards/HALLMARK_ADIPOGENESIS | 0.00279972 |
| chr7:45614104..45762734 | 107 | ADCY1 Adenylate_cyclase_1_(brain) | BIOCARTA_EDG1_PATHWAY Http://www.broadinstitute.org/gsea/msigdb/cards/BIOCARTA_EDG1_PATHWAY | 0.00289971 |
| chr8:17913904..17942527 | 427 | ASAH1 N-acylsphingosine_amidohydrolase_(acid_ceramidase)_1 | BIOCARTA_EDG1_PATHWAY Http://www.broadinstitute.org/gsea/msigdb/cards/BIOCARTA_EDG1_PATHWAY | 0.00289971 |
| chr17:64298905..64806882 | 5578 | PRKCA Protein_kinase_C,_alpha | BIOCARTA_EDG1_PATHWAY Http://www.broadinstitute.org/gsea/msigdb/cards/BIOCARTA_EDG1_PATHWAY | 0.00289971 |
| chr10:89623174..89728552 | 5728 | PTEN Phosphatase_and_tensin_homolog | 04070 Phosphatidylinositol_signaling_system | 0.00379962 |
| chr17:64298905..64806882 | 5578 | PRKCA Protein_kinase_C,_alpha | 04070 Phosphatidylinositol_signaling_system | 0.00379962 |
| chr20:39766140..39804377 | 5335 | PLCG1 Phospholipase_C,_gamma_1 | 04070 Phosphatidylinositol_signaling_system | 0.00379962 |
| chr1:108113761..108507565 | 10451 | VAV3 Vav_3_guanine_nucleotide_exchange_factor | HALLMARK_PI3K_AKT_MTOR_SIGNALING Http://www.broadinstitute.org/gsea/msigdb/cards/HALLMARK_PI3K_AKT_MTOR_SIGNALING | 0.00379962 |
| chr2:121010393..121052306 | 5899 | RALB V-ral_simian_leukemia_viral_oncogene_homolog_B_(ras_related;_GTP_binding_protein) | HALLMARK_PI3K_AKT_MTOR_SIGNALING Http://www.broadinstitute.org/gsea/msigdb/cards/HALLMARK_PI3K_AKT_MTOR_SIGNALING | 0.00379962 |
| chr6:2919215..31637863 | 1460, 8737 | CSNK2B,RIPK1 Casein_kinase_2,_beta_polypeptide,receptor_(TNFRSF)-interacting_serine-threonine_kinase_1 | HALLMARK_PI3K_AKT_MTOR_SIGNALING Http://www.broadinstitute.org/gsea/msigdb/cards/HALLMARK_PI3K_AKT_MTOR_SIGNALING | 0.00379962 |
| chr10:89623174..89728552 | 5728 | PTEN Phosphatase_and_tensin_homolog | HALLMARK_PI3K_AKT_MTOR_SIGNALING Http://www.broadinstitute.org/gsea/msigdb/cards/HALLMARK_PI3K_AKT_MTOR_SIGNALING | 0.00379962 |
| chr17:35441906..35766922 | 31 | ACACA Acetyl-CoA_carboxylase_alpha | HALLMARK_PI3K_AKT_MTOR_SIGNALING Http://www.broadinstitute.org/gsea/msigdb/cards/HALLMARK_PI3K_AKT_MTOR_SIGNALING | 0.00379962 |
| chr20:39766140..39804377 | 5335 | PLCG1 Phospholipase_C,_gamma_1 | HALLMARK_PI3K_AKT_MTOR_SIGNALING Http://www.broadinstitute.org/gsea/msigdb/cards/HALLMARK_PI3K_AKT_MTOR_SIGNALING | 0.00379962 |
| chr6:146348761..146758751 | 2911 | GRM1 Glutamate_receptor,_metabotropic_1 | 04020 Calcium_signaling_pathway | 0.0039996 |
| chr11:88237723..88796836 | 2915 | GRM5 Glutamate_receptor,_metabotropic_5 | 04020 Calcium_signaling_pathway | 0.0039996 |
| chr17:64298905..64806882 | 5578 | PRKCA Protein_kinase_C,_alpha | 04020 Calcium_signaling_pathway | 0.0039996 |
| chr20:39766140..39804377 | 5335 | PLCG1 Phospholipase_C,_gamma_1 | 04020 Calcium_signaling_pathway | 0.0039996 |
| chr17:762260..44105719 | 4137, 6777, 6776, 6770 | MAPT,STAT5B,STAT5A,STAT3 Microtubule-associated_protein_tau,signal_transducer_and_activator_of_transcription_5B,signal_transducer_and_activator_of_transcription_5A,signal_transducer_and_activator_of_transcription_3_(acute-phase_response_factor) | BIOCARTA_BIOPEPTIDES_PATHWAY Http://www.broadinstitute.org/gsea/msigdb/cards/BIOCARTA_BIOPEPTIDES_PATHWAY | 0.0153985 |
| chr17:64298905..64806882 | 5578 | PRKCA Protein_kinase_C,_alpha | BIOCARTA_BIOPEPTIDES_PATHWAY Http://www.broadinstitute.org/gsea/msigdb/cards/BIOCARTA_BIOPEPTIDES_PATHWAY | 0.0153985 |
| chr20:39766140..39804377 | 5335 | PLCG1 Phospholipase_C,_gamma_1 | BIOCARTA_BIOPEPTIDES_PATHWAY Http://www.broadinstitute.org/gsea/msigdb/cards/BIOCARTA_BIOPEPTIDES_PATHWAY | 0.0153985 |

Table S3 Enriched gene sets with corrected P <= 0.1

| Gene Set Collection | P_T_ | R^2^ | P_corr_ | T_Size | Int_No | P | Gene set |
| --- | --- | --- | --- | --- | --- | --- | --- |
| ***NDRI responders (case) vs. non-responders (control)*** | | | | | | | |
| kegg.set | 0.00005 | 0.2 | 0.0557888 | 93 | 4 | 0.00189981 | 04666 Fc_gamma_R-mediated_phagocytosis |
| kegg.set | 0.00005 | 0.2 | 0.0383923 | 67 | 5 | 0.00119988 | 04730 Long-term_depression |
| kegg.set | 0.00005 | 0.3 | 0.0789842 | 77 | 3 | 0.00379962 | 04070 Phosphatidylinositol_signaling_system |
| kegg.set | 0.00005 | 0.3 | 0.0543891 | 67 | 5 | 0.00169983 | 04730 Long-term_depression |
| kegg.set | 0.00005 | 0.5 | 0.0995801 | 167 | 4 | 0.0039996 | 04020 Calcium_signaling_pathway |
| kegg.set | 0.00005 | 0.5 | 0.0211958 | 67 | 5 | 0.00059994 | 04730 Long-term_depression |
| kegg.set | 0.0001 | 0.2 | 0.0595881 | 22 | 3 | 0.00109989 | 04710 Circadian_rhythm_-_mammal |
| kegg.set | 0.0001 | 0.3 | 0.0741852 | 22 | 3 | 0.00109989 | 04710 Circadian_rhythm_-_mammal |
| kegg.set | 0.0001 | 0.5 | 0.0525895 | 22 | 3 | 0.00119988 | 04710 Circadian_rhythm_-_mammal |
| go.set | 0.00005 | 0.2 | 0.089982 | 115 | 6 | 0.00019998 | GO:0001525 angiogenesis |
| go.set | 0.00005 | 0.3 | 0.0867826 | 11 | 3 | 0.00019998 | GO:0005161 platelet-derived |
| c2.cp.biocarta.v5.0.entrez.gmt.msig.set | 0.00005 | 0.2 | 0.0715857 | 38 | 3 | 0.0148985 | BIOCARTA_BIOPEPTIDES_PATHWAY http://www.broadinstitute.org/gsea/msigdb/cards/BIOCARTA_BIOPEPTIDES_PATHWAY |
| c2.cp.biocarta.v5.0.entrez.gmt.msig.set | 0.00005 | 0.3 | 0.0661868 | 38 | 3 | 0.0148985 | BIOCARTA_BIOPEPTIDES_PATHWAY http://www.broadinstitute.org/gsea/msigdb/cards/BIOCARTA_BIOPEPTIDES_PATHWAY |
| c2.cp.biocarta.v5.0.entrez.gmt.msig.set | 0.00005 | 0.5 | 0.0627874 | 38 | 3 | 0.0153985 | BIOCARTA_BIOPEPTIDES_PATHWAY http://www.broadinstitute.org/gsea/msigdb/cards/BIOCARTA_BIOPEPTIDES_PATHWAY |
| c2.cp.biocarta.v5.0.entrez.gmt.msig.set | 0.0001 | 0.2 | 0.0807838 | 27 | 3 | 0.00189981 | BIOCARTA_VEGF_PATHWAY http://www.broadinstitute.org/gsea/msigdb/cards/BIOCARTA_VEGF_PATHWAY |
| c2.cp.biocarta.v5.0.entrez.gmt.msig.set | 0.0001 | 0.3 | 0.0381924 | 27 | 3 | 0.0009999 | BIOCARTA_VEGF_PATHWAY http://www.broadinstitute.org/gsea/msigdb/cards/BIOCARTA_VEGF_PATHWAY |
| c2.cp.biocarta.v5.0.entrez.gmt.msig.set | 0.0001 | 0.5 | 0.0741852 | 27 | 3 | 0.00289971 | BIOCARTA_EDG1_PATHWAY http://www.broadinstitute.org/gsea/msigdb/cards/BIOCARTA_EDG1_PATHWAY |
| c2.cp.biocarta.v5.0.entrez.gmt.msig.set | 0.0001 | 0.5 | 0.0405919 | 27 | 3 | 0.00089991 | BIOCARTA_VEGF_PATHWAY http://www.broadinstitute.org/gsea/msigdb/cards/BIOCARTA_VEGF_PATHWAY |
| c2.cp.kegg.v5.0.entrez.gmt.msig.set | 0.00005 | 0.2 | 0.0593881 | 95 | 4 | 0.00189981 | KEGG_FC_GAMMA_R_MEDIATED_PHAGOCYTOSIS http://www.broadinstitute.org/gsea/msigdb/cards/KEGG_FC_GAMMA_R_MEDIATED_PHAGOCYTOSIS |
| c2.cp.kegg.v5.0.entrez.gmt.msig.set | 0.00005 | 0.2 | 0.0357928 | 67 | 5 | 0.0009999 | KEGG_LONG_TERM_DEPRESSION http://www.broadinstitute.org/gsea/msigdb/cards/KEGG_LONG_TERM_DEPRESSION |
| c2.cp.kegg.v5.0.entrez.gmt.msig.set | 0.00005 | 0.3 | 0.0589882 | 67 | 5 | 0.00219978 | KEGG_LONG_TERM_DEPRESSION http://www.broadinstitute.org/gsea/msigdb/cards/KEGG_LONG_TERM_DEPRESSION |
| c2.cp.kegg.v5.0.entrez.gmt.msig.set | 0.00005 | 0.3 | 0.0715857 | 75 | 3 | 0.00269973 | KEGG_PHOSPHATIDYLINOSITOL_SIGNALING_SYSTEM http://www.broadinstitute.org/gsea/msigdb/cards/KEGG_PHOSPHATIDYLINOSITOL_SIGNALING_SYSTEM |
| c2.cp.kegg.v5.0.entrez.gmt.msig.set | 0.00005 | 0.5 | 0.0171966 | 67 | 5 | 0.00049995 | KEGG_LONG_TERM_DEPRESSION http://www.broadinstitute.org/gsea/msigdb/cards/KEGG_LONG_TERM_DEPRESSION |
| c2.cp.reactome.v5.0.entrez.gmt.msig.set | 0.00005 | 0.5 | 0.0679864 | 100 | 4 | 0.00219978 | REACTOME_SIGNALING_BY_RHO_GTPASES http://www.broadinstitute.org/gsea/msigdb/cards/REACTOME_SIGNALING_BY_RHO_GTPASES |
| c2.cp.reactome.v5.0.entrez.gmt.msig.set | 0.0005 | 0.2 | 0.0887822 | 15 | 7 | 0.00059994 | REACTOME_PLATELET_CALCIUM_HOMEOSTASIS http://www.broadinstitute.org/gsea/msigdb/cards/REACTOME_PLATELET_CALCIUM_HOMEOSTASIS |
| c2.cp.reactome.v5.0.entrez.gmt.msig.set | 0.0005 | 0.3 | 0.0777844 | 15 | 7 | 0.00039996 | REACTOME_PLATELET_CALCIUM_HOMEOSTASIS http://www.broadinstitute.org/gsea/msigdb/cards/REACTOME_PLATELET_CALCIUM_HOMEOSTASIS |
| c2.cp.v5.0.entrez.gmt.msig.set | 0.00005 | 0.2 | 0.0369926 | 25 | 3 | 0.00019998 | PID_CDC42_REG_PATHWAY http://www.broadinstitute.org/gsea/msigdb/cards/PID_CDC42_REG_PATHWAY |
| c2.cp.v5.0.entrez.gmt.msig.set | 0.00005 | 0.3 | 0.0379924 | 25 | 3 | 0.00019998 | PID_CDC42_REG_PATHWAY http://www.broadinstitute.org/gsea/msigdb/cards/PID_CDC42_REG_PATHWAY |
| c2.cp.v5.0.entrez.gmt.msig.set | 0.00005 | 0.5 | 0.0643871 | 25 | 3 | 0.00049995 | PID_CDC42_REG_PATHWAY http://www.broadinstitute.org/gsea/msigdb/cards/PID_CDC42_REG_PATHWAY |
| c1.all.v5.0.entrez.gmt.msig.set | 0.0005 | 0.2 | 0.0163967* | 34 | 8 | 1.00E-04 | chr8p22 http://www.broadinstitute.org/gsea/msigdb/cards/chr8p22 |
| c1.all.v5.0.entrez.gmt.msig.set | 0.0005 | 0.3 | 0.0773845 | 86 | 6 | 0.00079992 | chr1q42 http://www.broadinstitute.org/gsea/msigdb/cards/chr1q42 |
| c1.all.v5.0.entrez.gmt.msig.set | 0.0005 | 0.3 | 0.0135973* | 34 | 8 | 1.00E-04 | chr8p22 http://www.broadinstitute.org/gsea/msigdb/cards/chr8p22 |
| c1.all.v5.0.entrez.gmt.msig.set | 0.0005 | 0.5 | 0.0167966* | 34 | 8 | 1.00E-04 | chr8p22 http://www.broadinstitute.org/gsea/msigdb/cards/chr8p22 |
| c3.all.v5.0.entrez.gmt.msig.set | 0.00005 | 0.2 | 0.094781 | 85 | 5 | 0.00089991 | GGCCAGT,MIR-193A,MIR-193B http://www.broadinstitute.org/gsea/msigdb/cards/GGCCAGT,MIR-193A,MIR-193B |
| c3.mir.v5.0.entrez.gmt.msig.set | 0.00005 | 0.2 | 0.04999 | 85 | 5 | 0.0009999 | GGCCAGT,MIR-193A,MIR-193B http://www.broadinstitute.org/gsea/msigdb/cards/GGCCAGT,MIR-193A,MIR-193B |
| c3.mir.v5.0.entrez.gmt.msig.set | 0.00005 | 0.3 | 0.0695861 | 85 | 5 | 0.00129987 | GGCCAGT,MIR-193A,MIR-193B http://www.broadinstitute.org/gsea/msigdb/cards/GGCCAGT,MIR-193A,MIR-193B |
| c5.all.v5.0.entrez.gmt.msig.set | 0.00005 | 0.3 | 0.0977804 | 39 | 3 | 0.00129987 | SH3_SH2_ADAPTOR_ACTIVITY http://www.broadinstitute.org/gsea/msigdb/cards/SH3_SH2_ADAPTOR_ACTIVITY |
| c5.all.v5.0.entrez.gmt.msig.set | 0.00005 | 0.5 | 0.0971806 | 45 | 3 | 0.00139986 | MOLECULAR_ADAPTOR_ACTIVITY http://www.broadinstitute.org/gsea/msigdb/cards/MOLECULAR_ADAPTOR_ACTIVITY |
| c5.all.v5.0.entrez.gmt.msig.set | 0.00005 | 0.5 | 0.0971806 | 55 | 3 | 0.00139986 | PROTEIN_BINDING_BRIDGING http://www.broadinstitute.org/gsea/msigdb/cards/PROTEIN_BINDING_BRIDGING |
| c5.all.v5.0.entrez.gmt.msig.set | 0.00005 | 0.5 | 0.0783843 | 39 | 3 | 0.0009999 | SH3_SH2_ADAPTOR_ACTIVITY http://www.broadinstitute.org/gsea/msigdb/cards/SH3_SH2_ADAPTOR_ACTIVITY |
| c5.all.v5.0.entrez.gmt.msig.set | 0.0005 | 0.5 | 0.0775845 | 16 | 5 | 0.00019998 | LIPID_HOMEOSTASIS http://www.broadinstitute.org/gsea/msigdb/cards/LIPID_HOMEOSTASIS |
| c5.bp.v5.0.entrez.gmt.msig.set | 0.0005 | 0.2 | 0.0787842 | 16 | 5 | 0.00039996 | LIPID_HOMEOSTASIS http://www.broadinstitute.org/gsea/msigdb/cards/LIPID_HOMEOSTASIS |
| c5.bp.v5.0.entrez.gmt.msig.set | 0.0005 | 0.2 | 0.0911818 | 20 | 5 | 0.00049995 | SENSORY_PERCEPTION_OF_CHEMICAL_STIMULUS http://www.broadinstitute.org/gsea/msigdb/cards/SENSORY_PERCEPTION_OF_CHEMICAL_STIMULUS |
| c5.bp.v5.0.entrez.gmt.msig.set | 0.0005 | 0.3 | 0.0413917* | 16 | 5 | 1.00E-04 | LIPID_HOMEOSTASIS http://www.broadinstitute.org/gsea/msigdb/cards/LIPID_HOMEOSTASIS |
| c5.bp.v5.0.entrez.gmt.msig.set | 0.0005 | 0.5 | 0.0403919 | 16 | 5 | 0.00019998 | LIPID_HOMEOSTASIS http://www.broadinstitute.org/gsea/msigdb/cards/LIPID_HOMEOSTASIS |
| c5.bp.v5.0.entrez.gmt.msig.set | 0.0005 | 0.5 | 0.0561888 | 20 | 5 | 0.00029997 | SENSORY_PERCEPTION_OF_CHEMICAL_STIMULUS http://www.broadinstitute.org/gsea/msigdb/cards/SENSORY_PERCEPTION_OF_CHEMICAL_STIMULUS |
| c5.mf.v5.0.entrez.gmt.msig.set | 0.00005 | 0.2 | 0.0419916 | 45 | 3 | 0.00159984 | MOLECULAR_ADAPTOR_ACTIVITY http://www.broadinstitute.org/gsea/msigdb/cards/MOLECULAR_ADAPTOR_ACTIVITY |
| c5.mf.v5.0.entrez.gmt.msig.set | 0.00005 | 0.2 | 0.0515897 | 55 | 3 | 0.00189981 | PROTEIN_BINDING_BRIDGING http://www.broadinstitute.org/gsea/msigdb/cards/PROTEIN_BINDING_BRIDGING |
| c5.mf.v5.0.entrez.gmt.msig.set | 0.00005 | 0.2 | 0.035193 | 39 | 3 | 0.00119988 | SH3_SH2_ADAPTOR_ACTIVITY http://www.broadinstitute.org/gsea/msigdb/cards/SH3_SH2_ADAPTOR_ACTIVITY |
| c5.mf.v5.0.entrez.gmt.msig.set | 0.00005 | 0.3 | 0.0703859 | 45 | 3 | 0.00239976 | MOLECULAR_ADAPTOR_ACTIVITY http://www.broadinstitute.org/gsea/msigdb/cards/MOLECULAR_ADAPTOR_ACTIVITY |
| c5.mf.v5.0.entrez.gmt.msig.set | 0.00005 | 0.3 | 0.0615877 | 55 | 3 | 0.00209979 | PROTEIN_BINDING_BRIDGING http://www.broadinstitute.org/gsea/msigdb/cards/PROTEIN_BINDING_BRIDGING |
| c5.mf.v5.0.entrez.gmt.msig.set | 0.00005 | 0.3 | 0.0523895 | 39 | 3 | 0.00169983 | SH3_SH2_ADAPTOR_ACTIVITY http://www.broadinstitute.org/gsea/msigdb/cards/SH3_SH2_ADAPTOR_ACTIVITY |
| c5.mf.v5.0.entrez.gmt.msig.set | 0.00005 | 0.5 | 0.0345931 | 45 | 3 | 0.00119988 | MOLECULAR_ADAPTOR_ACTIVITY http://www.broadinstitute.org/gsea/msigdb/cards/MOLECULAR_ADAPTOR_ACTIVITY |
| c5.mf.v5.0.entrez.gmt.msig.set | 0.00005 | 0.5 | 0.0303939 | 55 | 3 | 0.00089991 | PROTEIN_BINDING_BRIDGING http://www.broadinstitute.org/gsea/msigdb/cards/PROTEIN_BINDING_BRIDGING |
| c5.mf.v5.0.entrez.gmt.msig.set | 0.00005 | 0.5 | 0.0237952 | 39 | 3 | 0.00069993 | SH3_SH2_ADAPTOR_ACTIVITY http://www.broadinstitute.org/gsea/msigdb/cards/SH3_SH2_ADAPTOR_ACTIVITY |
| c5.mf.v5.0.entrez.gmt.msig.set | 0.0001 | 0.5 | 0.080184 | 55 | 5 | 0.00129987 | PROTEIN_BINDING_BRIDGING http://www.broadinstitute.org/gsea/msigdb/cards/PROTEIN_BINDING_BRIDGING |
| h.all.v5.0.entrez.gmt.msig.set | 0.00005 | 0.2 | 0.0513897 | 195 | 4 | 0.00329967 | HALLMARK_ADIPOGENESIS http://www.broadinstitute.org/gsea/msigdb/cards/HALLMARK_ADIPOGENESIS |
| h.all.v5.0.entrez.gmt.msig.set | 0.0001 | 0.3 | 0.0757848 | 102 | 6 | 0.00379962 | HALLMARK_PI3K_AKT_MTOR_SIGNALING http://www.broadinstitute.org/gsea/msigdb/cards/HALLMARK_PI3K_AKT_MTOR_SIGNALING |
| h.all.v5.0.entrez.gmt.msig.set | 0.0001 | 0.5 | 0.0475905 | 195 | 6 | 0.00279972 | HALLMARK_ADIPOGENESIS http://www.broadinstitute.org/gsea/msigdb/cards/HALLMARK_ADIPOGENESIS |
| ***SSRI responders (case) vs. non-responders (control)*** | | | | | | | |
| c1.all.v5.0.entrez.gmt.msig.set | 0.0005 | 0.2 | 0.0969806 | 74 | 6 | 0.00109989 | chr18q21 http://www.broadinstitute.org/gsea/msigdb/cards/chr18q21 |
| c3.mir.v5.0.entrez.gmt.msig.set | 0.0005 | 0.2 | 0.0665867 | 115 | 15 | 0.00079992 | GCACCTT,MIR-18A,MIR-18B http://www.broadinstitute.org/gsea/msigdb/cards/GCACCTT,MIR-18A,MIR-18B |
| c3.mir.v5.0.entrez.gmt.msig.set | 0.0005 | 0.3 | 0.074785 | 115 | 15 | 0.0009999 | GCACCTT,MIR-18A,MIR-18B http://www.broadinstitute.org/gsea/msigdb/cards/GCACCTT,MIR-18A,MIR-18B |
| c5.all.v5.0.entrez.gmt.msig.set | 0.0005 | 0.5 | 0.0865827 | 17 | 4 | 0.00019998 | NEGATIVE_REGULATION_OF_DNA_METABOLIC_PROCESS http://www.broadinstitute.org/gsea/msigdb/cards/NEGATIVE_REGULATION_OF_DNA_METABOLIC_PROCESS |
| c5.bp.v5.0.entrez.gmt.msig.set | 0.0005 | 0.5 | 0.0923815 | 17 | 4 | 0.00049995 | NEGATIVE_REGULATION_OF_DNA_METABOLIC_PROCESS http://www.broadinstitute.org/gsea/msigdb/cards/NEGATIVE_REGULATION_OF_DNA_METABOLIC_PROCESS |
| h.all.v5.0.entrez.gmt.msig.set | 0.00005 | 0.2 | 0.0511898 | 190 | 8 | 0.00309969 | HALLMARK_E2F_TARGETS http://www.broadinstitute.org/gsea/msigdb/cards/HALLMARK_E2F_TARGETS |
| h.all.v5.0.entrez.gmt.msig.set | 0.00005 | 0.3 | 0.0203959 | 190 | 8 | 0.00119988 | HALLMARK_E2F_TARGETS http://www.broadinstitute.org/gsea/msigdb/cards/HALLMARK_E2F_TARGETS |
| h.all.v5.0.entrez.gmt.msig.set | 0.00005 | 0.5 | 0.0845831 | 190 | 7 | 0.00549945 | HALLMARK_E2F_TARGETS http://www.broadinstitute.org/gsea/msigdb/cards/HALLMARK_E2F_TARGETS |
| ***citalopram or escitalopram responders (case) vs. non-responders (control)*** | | | | | | | |
| kegg.set | 0.0001 | 0.2 | 0.0705859 | 25 | 5 | 0.00119988 | 00534 Glycosaminoglycan_biosynthesis_-_heparan_sulfate |
| kegg.set | 0.0001 | 0.3 | 0.064787 | 25 | 5 | 0.00109989 | 00534 Glycosaminoglycan_biosynthesis_-_heparan_sulfate |
| kegg.set | 0.0005 | 0.3 | 0.0513897 | 10 | 3 | 0.00059994 | 00430 Taurine_and_hypotaurine_metabolism |
| kegg.set | 0.0005 | 0.3 | 0.0275945 | 7 | 4 | 0.00029997 | 00460 Cyanoamino_acid_metabolism |
| kegg.set | 0.0005 | 0.5 | 0.0639872 | 10 | 3 | 0.0009999 | 00430 Taurine_and_hypotaurine_metabolism |
| kegg.set | 0.0005 | 0.5 | 0.0387922 | 7 | 4 | 0.00049995 | 00460 Cyanoamino_acid_metabolism |
| c2.cp.kegg.v5.0.entrez.gmt.msig.set | 0.0001 | 0.2 | 0.0517896 | 25 | 5 | 0.00089991 | KEGG_GLYCOSAMINOGLYCAN_BIOSYNTHESIS_HEPARAN_SULFATE http://www.broadinstitute.org/gsea/msigdb/cards/KEGG_GLYCOSAMINOGLYCAN_BIOSYNTHESIS_HEPARAN_SULFATE |
| c2.cp.kegg.v5.0.entrez.gmt.msig.set | 0.0001 | 0.3 | 0.0303939 | 25 | 5 | 0.00049995 | KEGG_GLYCOSAMINOGLYCAN_BIOSYNTHESIS_HEPARAN_SULFATE http://www.broadinstitute.org/gsea/msigdb/cards/KEGG_GLYCOSAMINOGLYCAN_BIOSYNTHESIS_HEPARAN_SULFATE |
| c2.cp.kegg.v5.0.entrez.gmt.msig.set | 0.0005 | 0.3 | 0.0929814 | 22 | 5 | 0.00179982 | KEGG_BIOSYNTHESIS_OF_UNSATURATED_FATTY_ACIDS http://www.broadinstitute.org/gsea/msigdb/cards/KEGG_BIOSYNTHESIS_OF_UNSATURATED_FATTY_ACIDS |
| c2.cp.kegg.v5.0.entrez.gmt.msig.set | 0.0005 | 0.3 | 0.0487902 | 10 | 3 | 0.00079992 | KEGG_TAURINE_AND_HYPOTAURINE_METABOLISM http://www.broadinstitute.org/gsea/msigdb/cards/KEGG_TAURINE_AND_HYPOTAURINE_METABOLISM |
| c2.cp.kegg.v5.0.entrez.gmt.msig.set | 0.0005 | 0.5 | 0.0453909 | 10 | 3 | 0.00069993 | KEGG_TAURINE_AND_HYPOTAURINE_METABOLISM http://www.broadinstitute.org/gsea/msigdb/cards/KEGG_TAURINE_AND_HYPOTAURINE_METABOLISM |
| c2.cp.reactome.v5.0.entrez.gmt.msig.set | 0.0005 | 0.2 | 0.0281944* | 13 | 4 | 0.00009999 | REACTOME_NUCLEOTIDE_LIKE_PURINERGIC_RECEPTORS http://www.broadinstitute.org/gsea/msigdb/cards/REACTOME_NUCLEOTIDE_LIKE_PURINERGIC_RECEPTORS |
| c2.cp.reactome.v5.0.entrez.gmt.msig.set | 0.0005 | 0.3 | 0.0279944* | 13 | 4 | 0.00009999 | REACTOME_NUCLEOTIDE_LIKE_PURINERGIC_RECEPTORS http://www.broadinstitute.org/gsea/msigdb/cards/REACTOME_NUCLEOTIDE_LIKE_PURINERGIC_RECEPTORS |
| c2.cp.reactome.v5.0.entrez.gmt.msig.set | 0.0005 | 0.5 | 0.0319936* | 13 | 4 | 0.00009999 | REACTOME_NUCLEOTIDE_LIKE_PURINERGIC_RECEPTORS http://www.broadinstitute.org/gsea/msigdb/cards/REACTOME_NUCLEOTIDE_LIKE_PURINERGIC_RECEPTORS |
| c2.cp.v5.0.entrez.gmt.msig.set | 0.0005 | 0.2 | 0.0913817 | 13 | 4 | 0.00019998 | REACTOME_NUCLEOTIDE_LIKE_PURINERGIC_RECEPTORS http://www.broadinstitute.org/gsea/msigdb/cards/REACTOME_NUCLEOTIDE_LIKE_PURINERGIC_RECEPTORS |
| c2.cp.v5.0.entrez.gmt.msig.set | 0.0005 | 0.3 | 0.0587882* | 10 | 3 | 0.00009999 | KEGG_TAURINE_AND_HYPOTAURINE_METABOLISM http://www.broadinstitute.org/gsea/msigdb/cards/KEGG_TAURINE_AND_HYPOTAURINE_METABOLISM |
| c2.cp.v5.0.entrez.gmt.msig.set | 0.0005 | 0.3 | 0.0811838 | 13 | 4 | 0.00019998 | REACTOME_NUCLEOTIDE_LIKE_PURINERGIC_RECEPTORS http://www.broadinstitute.org/gsea/msigdb/cards/REACTOME_NUCLEOTIDE_LIKE_PURINERGIC_RECEPTORS |
| c2.cp.v5.0.entrez.gmt.msig.set | 0.0005 | 0.5 | 0.0583883* | 13 | 4 | 0.00009999 | REACTOME_NUCLEOTIDE_LIKE_PURINERGIC_RECEPTORS http://www.broadinstitute.org/gsea/msigdb/cards/REACTOME_NUCLEOTIDE_LIKE_PURINERGIC_RECEPTORS |
| c1.all.v5.0.entrez.gmt.msig.set | 0.0005 | 0.2 | 0.014997* | 79 | 4 | 0.00009999 | chr16p12 http://www.broadinstitute.org/gsea/msigdb/cards/chr16p12 |
| c1.all.v5.0.entrez.gmt.msig.set | 0.0005 | 0.3 | 0.0287942 | 79 | 4 | 0.00019998 | chr16p12 http://www.broadinstitute.org/gsea/msigdb/cards/chr16p12 |
| c1.all.v5.0.entrez.gmt.msig.set | 0.0005 | 0.3 | 0.0535893 | 139 | 6 | 0.00049995 | chr22q11 http://www.broadinstitute.org/gsea/msigdb/cards/chr22q11 |
| c1.all.v5.0.entrez.gmt.msig.set | 0.0005 | 0.5 | 0.0167966* | 79 | 4 | 0.00009999 | chr16p12 http://www.broadinstitute.org/gsea/msigdb/cards/chr16p12 |
| c1.all.v5.0.entrez.gmt.msig.set | 0.0005 | 0.5 | 0.0321936 | 139 | 6 | 0.00029997 | chr22q11 http://www.broadinstitute.org/gsea/msigdb/cards/chr22q11 |
| ***non-TRD (case) vs. TRD (control)*** | | | | | | | |
| kegg.set | 0.0005 | 0.2 | 0.0437912 | 41 | 8 | 0.00059994 | 00071 Fatty_acid_metabolism |
| kegg.set | 0.0005 | 0.3 | 0.0587882 | 41 | 8 | 0.00089991 | 00071 Fatty_acid_metabolism |
| kegg.set | 0.0005 | 0.5 | 0.0263947 | 41 | 8 | 0.00039996 | 00071 Fatty_acid_metabolism |
| c2.cp.kegg.v5.0.entrez.gmt.msig.set | 0.0005 | 0.2 | 0.0389922 | 40 | 8 | 0.00059994 | KEGG_FATTY_ACID_METABOLISM http://www.broadinstitute.org/gsea/msigdb/cards/KEGG_FATTY_ACID_METABOLISM |
| c2.cp.kegg.v5.0.entrez.gmt.msig.set | 0.0005 | 0.3 | 0.0373925 | 40 | 8 | 0.00049995 | KEGG_FATTY_ACID_METABOLISM http://www.broadinstitute.org/gsea/msigdb/cards/KEGG_FATTY_ACID_METABOLISM |
| c4.all.v5.0.entrez.gmt.msig.set | 0.0005 | 0.2 | 0.0843831 | 26 | 4 | 0.00039996 | MODULE_68 http://www.broadinstitute.org/gsea/msigdb/cards/MODULE_68 |
| c5.all.v5.0.entrez.gmt.msig.set | 0.0005 | 0.2 | 0.0857828 | 11 | 3 | 0.00019998 | ENDONUCLEASE_ACTIVITY_GO_0016893 http://www.broadinstitute.org/gsea/msigdb/cards/ENDONUCLEASE_ACTIVITY_GO_0016893 |
| c5.all.v5.0.entrez.gmt.msig.set | 0.0005 | 0.2 | 0.0857828 | 13 | 3 | 0.00019998 | ENDORIBONUCLEASE_ACTIVITY http://www.broadinstitute.org/gsea/msigdb/cards/ENDORIBONUCLEASE_ACTIVITY |
| c5.all.v5.0.entrez.gmt.msig.set | 0.0005 | 0.5 | 0.0577884* | 25 | 5 | 0.00009999 | ENDONUCLEASE_ACTIVITY http://www.broadinstitute.org/gsea/msigdb/cards/ENDONUCLEASE_ACTIVITY |
| c5.bp.v5.0.entrez.gmt.msig.set | 0.0005 | 0.2 | 0.0789842 | 18 | 4 | 0.00039996 | REGULATION_OF_MAPKKK_CASCADE http://www.broadinstitute.org/gsea/msigdb/cards/REGULATION_OF_MAPKKK_CASCADE |
| c5.bp.v5.0.entrez.gmt.msig.set | 0.0005 | 0.3 | 0.0953809 | 123 | 14 | 0.00049995 | POSITIVE_REGULATION_OF_SIGNAL_TRANSDUCTION http://www.broadinstitute.org/gsea/msigdb/cards/POSITIVE_REGULATION_OF_SIGNAL_TRANSDUCTION |
| ***NDRI non-responder vs. healthy controls*** | | | | | | | |
| c2.cgp.v5.0.entrez.gmt.msig.set | 0.00001 | 0.3 | 0.0861828 | 104 | 3 | 0.00029997 | LABBE_TARGETS_OF_TGFB1_AND_WNT3A_UP http://www.broadinstitute.org/gsea/msigdb/cards/LABBE_TARGETS_OF_TGFB1_AND_WNT3A_UP |
| c2.cp.kegg.v5.0.entrez.gmt.msig.set | 0.0005 | 0.2 | 0.0857828 | 24 | 5 | 0.00159984 | KEGG_NICOTINATE_AND_NICOTINAMIDE_METABOLISM http://www.broadinstitute.org/gsea/msigdb/cards/KEGG_NICOTINATE_AND_NICOTINAMIDE_METABOLISM |
| c2.cp.reactome.v5.0.entrez.gmt.msig.set | 0.0005 | 0.2 | 0.0583883 | 14 | 3 | 0.00029997 | REACTOME_VIRAL_MESSENGER_RNA_SYNTHESIS http://www.broadinstitute.org/gsea/msigdb/cards/REACTOME_VIRAL_MESSENGER_RNA_SYNTHESIS |
| c2.cp.reactome.v5.0.entrez.gmt.msig.set | 0.0005 | 0.3 | 0.0465907 | 14 | 3 | 0.00029997 | REACTOME_VIRAL_MESSENGER_RNA_SYNTHESIS http://www.broadinstitute.org/gsea/msigdb/cards/REACTOME_VIRAL_MESSENGER_RNA_SYNTHESIS |
| c2.cp.reactome.v5.0.entrez.gmt.msig.set | 0.0005 | 0.5 | 0.0665867 | 14 | 3 | 0.00039996 | REACTOME_VIRAL_MESSENGER_RNA_SYNTHESIS http://www.broadinstitute.org/gsea/msigdb/cards/REACTOME_VIRAL_MESSENGER_RNA_SYNTHESIS |
| c2.cp.v5.0.entrez.gmt.msig.set | 0.0005 | 0.5 | 0.079784 | 47 | 8 | 0.00019998 | PID_AJDISS_2PATHWAY http://www.broadinstitute.org/gsea/msigdb/cards/PID_AJDISS_2PATHWAY |
| c1.all.v5.0.entrez.gmt.msig.set | 0.00005 | 0.2 | 0.0227954 | 190 | 5 | 0.00029997 | chr21q22 http://www.broadinstitute.org/gsea/msigdb/cards/chr21q22 |
| c1.all.v5.0.entrez.gmt.msig.set | 0.00005 | 0.3 | 0.0207958 | 190 | 5 | 0.00019998 | chr21q22 http://www.broadinstitute.org/gsea/msigdb/cards/chr21q22 |
| c1.all.v5.0.entrez.gmt.msig.set | 0.00005 | 0.5 | 0.0117976* | 190 | 6 | 0.00009999 | chr21q22 http://www.broadinstitute.org/gsea/msigdb/cards/chr21q22 |
| c1.all.v5.0.entrez.gmt.msig.set | 0.0001 | 0.5 | 0.0163967 | 190 | 6 | 0.00019998 | chr21q22 http://www.broadinstitute.org/gsea/msigdb/cards/chr21q22 |
| c1.all.v5.0.entrez.gmt.msig.set | 0.0005 | 0.2 | 0.0275945 | 76 | 6 | 0.00019998 | chr1p32 http://www.broadinstitute.org/gsea/msigdb/cards/chr1p32 |
| c1.all.v5.0.entrez.gmt.msig.set | 0.0005 | 0.2 | 0.0981804 | 86 | 6 | 0.00109989 | chr1q42 http://www.broadinstitute.org/gsea/msigdb/cards/chr1q42 |
| c1.all.v5.0.entrez.gmt.msig.set | 0.0005 | 0.3 | 0.0237952 | 76 | 6 | 0.00019998 | chr1p32 http://www.broadinstitute.org/gsea/msigdb/cards/chr1p32 |
| c1.all.v5.0.entrez.gmt.msig.set | 0.0005 | 0.3 | 0.0881824 | 86 | 6 | 0.0009999 | chr1q42 http://www.broadinstitute.org/gsea/msigdb/cards/chr1q42 |
| c1.all.v5.0.entrez.gmt.msig.set | 0.0005 | 0.5 | 0.0213957* | 76 | 6 | 0.00009999 | chr1p32 http://www.broadinstitute.org/gsea/msigdb/cards/chr1p32 |
| c1.all.v5.0.entrez.gmt.msig.set | 0.0005 | 0.5 | 0.0745851 | 86 | 6 | 0.00079992 | chr1q42 http://www.broadinstitute.org/gsea/msigdb/cards/chr1q42 |
| c5.all.v5.0.entrez.gmt.msig.set | 0.0005 | 0.2 | 0.0629874* | 15 | 6 | 0.00009999 | DENDRITE http://www.broadinstitute.org/gsea/msigdb/cards/DENDRITE |
| c5.all.v5.0.entrez.gmt.msig.set | 0.0005 | 0.2 | 0.0629874* | 13 | 7 | 0.00009999 | PROTEIN_TETRAMERIZATION http://www.broadinstitute.org/gsea/msigdb/cards/PROTEIN_TETRAMERIZATION |
| c5.all.v5.0.entrez.gmt.msig.set | 0.0005 | 0.3 | 0.0905819 | 15 | 6 | 0.00019998 | DENDRITE http://www.broadinstitute.org/gsea/msigdb/cards/DENDRITE |
| c5.all.v5.0.entrez.gmt.msig.set | 0.0005 | 0.3 | 0.0905819 | 21 | 8 | 0.00019998 | PROTEIN_HOMOOLIGOMERIZATION http://www.broadinstitute.org/gsea/msigdb/cards/PROTEIN_HOMOOLIGOMERIZATION |
| c5.all.v5.0.entrez.gmt.msig.set | 0.0005 | 0.3 | 0.0639872* | 13 | 7 | 0.00009999 | PROTEIN_TETRAMERIZATION http://www.broadinstitute.org/gsea/msigdb/cards/PROTEIN_TETRAMERIZATION |
| c5.all.v5.0.entrez.gmt.msig.set | 0.0005 | 0.5 | 0.0937812 | 26 | 5 | 0.00029997 | CASPASE_ACTIVATION http://www.broadinstitute.org/gsea/msigdb/cards/CASPASE_ACTIVATION |
| c5.all.v5.0.entrez.gmt.msig.set | 0.0005 | 0.5 | 0.0561888* | 15 | 6 | 0.00009999 | DENDRITE http://www.broadinstitute.org/gsea/msigdb/cards/DENDRITE |
| c5.all.v5.0.entrez.gmt.msig.set | 0.0005 | 0.5 | 0.0937812 | 37 | 6 | 0.00029997 | NEGATIVE_REGULATION_OF_SIGNAL_TRANSDUCTION http://www.broadinstitute.org/gsea/msigdb/cards/NEGATIVE_REGULATION_OF_SIGNAL_TRANSDUCTION |
| c5.all.v5.0.entrez.gmt.msig.set | 0.0005 | 0.5 | 0.0561888* | 13 | 7 | 0.00009999 | PROTEIN_TETRAMERIZATION http://www.broadinstitute.org/gsea/msigdb/cards/PROTEIN_TETRAMERIZATION |
| c5.bp.v5.0.entrez.gmt.msig.set | 0.0005 | 0.2 | 0.0339932* | 13 | 7 | 0.00009999 | PROTEIN_TETRAMERIZATION http://www.broadinstitute.org/gsea/msigdb/cards/PROTEIN_TETRAMERIZATION |
| c5.bp.v5.0.entrez.gmt.msig.set | 0.0005 | 0.3 | 0.064787 | 37 | 6 | 0.00029997 | NEGATIVE_REGULATION_OF_SIGNAL_TRANSDUCTION http://www.broadinstitute.org/gsea/msigdb/cards/NEGATIVE_REGULATION_OF_SIGNAL_TRANSDUCTION |
| c5.bp.v5.0.entrez.gmt.msig.set | 0.0005 | 0.3 | 0.0333933* | 13 | 7 | 0.00009999 | PROTEIN_TETRAMERIZATION http://www.broadinstitute.org/gsea/msigdb/cards/PROTEIN_TETRAMERIZATION |
| c5.bp.v5.0.entrez.gmt.msig.set | 0.0005 | 0.5 | 0.09978 | 26 | 5 | 0.00049995 | CASPASE_ACTIVATION http://www.broadinstitute.org/gsea/msigdb/cards/CASPASE_ACTIVATION |
| c5.bp.v5.0.entrez.gmt.msig.set | 0.0005 | 0.5 | 0.0719856 | 37 | 6 | 0.00029997 | NEGATIVE_REGULATION_OF_SIGNAL_TRANSDUCTION http://www.broadinstitute.org/gsea/msigdb/cards/NEGATIVE_REGULATION_OF_SIGNAL_TRANSDUCTION |
| c5.bp.v5.0.entrez.gmt.msig.set | 0.0005 | 0.5 | 0.0361928* | 13 | 7 | 0.00009999 | PROTEIN_TETRAMERIZATION http://www.broadinstitute.org/gsea/msigdb/cards/PROTEIN_TETRAMERIZATION |
| c5.mf.v5.0.entrez.gmt.msig.set | 0.0001 | 0.5 | 0.0903819 | 12 | 4 | 0.00109989 | ALDO_KETO_REDUCTASE_ACTIVITY http://www.broadinstitute.org/gsea/msigdb/cards/ALDO_KETO_REDUCTASE_ACTIVITY |
| ***NDRI responders vs. healthy controls*** | | | | | | | |
| c2.cp.kegg.v5.0.entrez.gmt.msig.set | 0.0005 | 0.2 | 0.069986 | 22 | 5 | 0.00119988 | KEGG_BIOSYNTHESIS_OF_UNSATURATED_FATTY_ACIDS http://www.broadinstitute.org/gsea/msigdb/cards/KEGG_BIOSYNTHESIS_OF_UNSATURATED_FATTY_ACIDS |
| c2.cp.reactome.v5.0.entrez.gmt.msig.set | 0.0005 | 0.5 | 0.0503899 | 13 | 4 | 0.00029997 | REACTOME_SIGNAL_ATTENUATION http://www.broadinstitute.org/gsea/msigdb/cards/REACTOME_SIGNAL_ATTENUATION |
| c3.mir.v5.0.entrez.gmt.msig.set | 0.0005 | 0.3 | 0.0335933 | 97 | 12 | 0.00029997 | TGCAAAC,MIR-452 http://www.broadinstitute.org/gsea/msigdb/cards/TGCAAAC,MIR-452 |
| c3.tft.v5.0.entrez.gmt.msig.set | 0.0005 | 0.2 | 0.0663867 | 164 | 16 | 0.00069993 | V$EVI1_05 http://www.broadinstitute.org/gsea/msigdb/cards/V$EVI1_05 |
| c3.tft.v5.0.entrez.gmt.msig.set | 0.0005 | 0.3 | 0.0541892 | 164 | 16 | 0.00059994 | V$EVI1_05 http://www.broadinstitute.org/gsea/msigdb/cards/V$EVI1_05 |
| c3.tft.v5.0.entrez.gmt.msig.set | 0.0005 | 0.5 | 0.0471906 | 164 | 16 | 0.00049995 | V$EVI1_05 http://www.broadinstitute.org/gsea/msigdb/cards/V$EVI1_05 |
| c5.all.v5.0.entrez.gmt.msig.set | 0.0005 | 0.5 | 0.0741852 | 11 | 4 | 0.00019998 | NUCLEAR_MATRIX http://www.broadinstitute.org/gsea/msigdb/cards/NUCLEAR_MATRIX |
| c5.cc.v5.0.entrez.gmt.msig.set | 0.0005 | 0.3 | 0.0819836 | 11 | 3 | 0.00159984 | NUCLEAR_MATRIX http://www.broadinstitute.org/gsea/msigdb/cards/NUCLEAR_MATRIX |
| c5.mf.v5.0.entrez.gmt.msig.set | 0.0005 | 0.2 | 0.0917816 | 55 | 10 | 0.00089991 | PROTEIN_BINDING_BRIDGING http://www.broadinstitute.org/gsea/msigdb/cards/PROTEIN_BINDING_BRIDGING |
| c5.mf.v5.0.entrez.gmt.msig.set | 0.0005 | 0.5 | 0.0611878 | 55 | 10 | 0.00049995 | PROTEIN_BINDING_BRIDGING http://www.broadinstitute.org/gsea/msigdb/cards/PROTEIN_BINDING_BRIDGING |
| ***SSRI non-responders vs. healthy controls*** | | | | | | | |
| c2.cp.kegg.v5.0.entrez.gmt.msig.set | 0.0005 | 0.3 | 0.0995801 | 22 | 5 | 0.0019998 | KEGG_BIOSYNTHESIS_OF_UNSATURATED_FATTY_ACIDS http://www.broadinstitute.org/gsea/msigdb/cards/KEGG_BIOSYNTHESIS_OF_UNSATURATED_FATTY_ACIDS |
| c2.cp.reactome.v5.0.entrez.gmt.msig.set | 0.0005 | 0.2 | 0.0521896 | 26 | 6 | 0.00029997 | REACTOME_RNA_POL_III_TRANSCRIPTION_INITIATION_FROM_TYPE_3_PROMOTER http://www.broadinstitute.org/gsea/msigdb/cards/REACTOME_RNA_POL_III_TRANSCRIPTION_INITIATION_FROM_TYPE_3_PROMOTER |
| c2.cp.reactome.v5.0.entrez.gmt.msig.set | 0.0005 | 0.3 | 0.0791842 | 28 | 5 | 0.00049995 | REACTOME_TIGHT_JUNCTION_INTERACTIONS http://www.broadinstitute.org/gsea/msigdb/cards/REACTOME_TIGHT_JUNCTION_INTERACTIONS |
| c2.cp.reactome.v5.0.entrez.gmt.msig.set | 0.0005 | 0.3 | 0.0939812 | 14 | 3 | 0.00059994 | REACTOME_VIRAL_MESSENGER_RNA_SYNTHESIS http://www.broadinstitute.org/gsea/msigdb/cards/REACTOME_VIRAL_MESSENGER_RNA_SYNTHESIS |
| c1.all.v5.0.entrez.gmt.msig.set | 0.0005 | 0.2 | 0.0243951 | 18 | 4 | 0.00019998 | chr10p14 http://www.broadinstitute.org/gsea/msigdb/cards/chr10p14 |
| c1.all.v5.0.entrez.gmt.msig.set | 0.0005 | 0.2 | 0.0857828 | 99 | 5 | 0.00089991 | chr3q21 http://www.broadinstitute.org/gsea/msigdb/cards/chr3q21 |
| c1.all.v5.0.entrez.gmt.msig.set | 0.0005 | 0.2 | 0.0169966* | 54 | 5 | 0.00009999 | chr6q21 http://www.broadinstitute.org/gsea/msigdb/cards/chr6q21 |
| c1.all.v5.0.entrez.gmt.msig.set | 0.0005 | 0.3 | 0.0653869 | 18 | 4 | 0.00069993 | chr10p14 http://www.broadinstitute.org/gsea/msigdb/cards/chr10p14 |
| c1.all.v5.0.entrez.gmt.msig.set | 0.0005 | 0.3 | 0.0653869 | 99 | 5 | 0.00069993 | chr3q21 http://www.broadinstitute.org/gsea/msigdb/cards/chr3q21 |
| c1.all.v5.0.entrez.gmt.msig.set | 0.0005 | 0.3 | 0.0175965* | 54 | 5 | 0.00009999 | chr6q21 http://www.broadinstitute.org/gsea/msigdb/cards/chr6q21 |
| c1.all.v5.0.entrez.gmt.msig.set | 0.0005 | 0.5 | 0.0273945 | 18 | 4 | 0.00019998 | chr10p14 http://www.broadinstitute.org/gsea/msigdb/cards/chr10p14 |
| c1.all.v5.0.entrez.gmt.msig.set | 0.0005 | 0.5 | 0.0823835 | 99 | 5 | 0.00089991 | chr3q21 http://www.broadinstitute.org/gsea/msigdb/cards/chr3q21 |
| c1.all.v5.0.entrez.gmt.msig.set | 0.0005 | 0.5 | 0.0273945 | 54 | 5 | 0.00019998 | chr6q21 http://www.broadinstitute.org/gsea/msigdb/cards/chr6q21 |
| c3.all.v5.0.entrez.gmt.msig.set | 0.0005 | 0.2 | 0.0641872 | 54 | 9 | 0.00029997 | V$EVI1_03 http://www.broadinstitute.org/gsea/msigdb/cards/V$EVI1_03 |
| c3.all.v5.0.entrez.gmt.msig.set | 0.0005 | 0.3 | 0.0753849 | 54 | 9 | 0.00039996 | V$EVI1_03 http://www.broadinstitute.org/gsea/msigdb/cards/V$EVI1_03 |
| c3.all.v5.0.entrez.gmt.msig.set | 0.0005 | 0.5 | 0.0923815 | 54 | 9 | 0.00049995 | V$EVI1_03 http://www.broadinstitute.org/gsea/msigdb/cards/V$EVI1_03 |
| c3.tft.v5.0.entrez.gmt.msig.set | 0.0005 | 0.5 | 0.0657868 | 54 | 8 | 0.00059994 | V$EVI1_03 http://www.broadinstitute.org/gsea/msigdb/cards/V$EVI1_03 |
| c5.all.v5.0.entrez.gmt.msig.set | 0.0005 | 0.2 | 0.0643871* | 22 | 8 | 0.00009999 | ACTIN_FILAMENT_BINDING http://www.broadinstitute.org/gsea/msigdb/cards/ACTIN_FILAMENT_BINDING |
| c5.all.v5.0.entrez.gmt.msig.set | 0.0005 | 0.2 | 0.0643871* | 24 | 6 | 0.00009999 | OXIDOREDUCTASE_ACTIVITY_ACTING_ON_NADH_OR_NADPH http://www.broadinstitute.org/gsea/msigdb/cards/OXIDOREDUCTASE_ACTIVITY_ACTING_ON_NADH_OR_NADPH |
| c5.all.v5.0.entrez.gmt.msig.set | 0.0005 | 0.2 | 0.0937812 | 19 | 5 | 0.00019998 | TRANSCRIPTION_FROM_RNA_POLYMERASE_III_PROMOTER http://www.broadinstitute.org/gsea/msigdb/cards/TRANSCRIPTION_FROM_RNA_POLYMERASE_III_PROMOTER |
| c5.all.v5.0.entrez.gmt.msig.set | 0.0005 | 0.3 | 0.0845831 | 22 | 8 | 0.00019998 | ACTIN_FILAMENT_BINDING http://www.broadinstitute.org/gsea/msigdb/cards/ACTIN_FILAMENT_BINDING |
| c5.all.v5.0.entrez.gmt.msig.set | 0.0005 | 0.3 | 0.0573885* | 19 | 5 | 0.00009999 | TRANSCRIPTION_FROM_RNA_POLYMERASE_III_PROMOTER http://www.broadinstitute.org/gsea/msigdb/cards/TRANSCRIPTION_FROM_RNA_POLYMERASE_III_PROMOTER |
| c5.bp.v5.0.entrez.gmt.msig.set | 0.0005 | 0.2 | 0.0557888 | 19 | 5 | 0.00019998 | TRANSCRIPTION_FROM_RNA_POLYMERASE_III_PROMOTER http://www.broadinstitute.org/gsea/msigdb/cards/TRANSCRIPTION_FROM_RNA_POLYMERASE_III_PROMOTER |
| c5.bp.v5.0.entrez.gmt.msig.set | 0.0005 | 0.3 | 0.094981 | 13 | 5 | 0.00049995 | PROTEIN_TETRAMERIZATION http://www.broadinstitute.org/gsea/msigdb/cards/PROTEIN_TETRAMERIZATION |
| c5.bp.v5.0.entrez.gmt.msig.set | 0.0005 | 0.3 | 0.0559888 | 19 | 5 | 0.00019998 | TRANSCRIPTION_FROM_RNA_POLYMERASE_III_PROMOTER http://www.broadinstitute.org/gsea/msigdb/cards/TRANSCRIPTION_FROM_RNA_POLYMERASE_III_PROMOTER |
| c5.bp.v5.0.entrez.gmt.msig.set | 0.0005 | 0.5 | 0.0753849 | 13 | 5 | 0.00039996 | PROTEIN_TETRAMERIZATION http://www.broadinstitute.org/gsea/msigdb/cards/PROTEIN_TETRAMERIZATION |
| c5.mf.v5.0.entrez.gmt.msig.set | 0.0005 | 0.3 | 0.0961808 | 22 | 7 | 0.00079992 | ACTIN_FILAMENT_BINDING http://www.broadinstitute.org/gsea/msigdb/cards/ACTIN_FILAMENT_BINDING |
| c7.all.v5.0.entrez.gmt.msig.set | 0.00005 | 0.3 | 0.0985803* | 188 | 12 | 0.00009999 | GSE27786_NKTCELL_VS_NEUTROPHIL_UP http://www.broadinstitute.org/gsea/msigdb/cards/GSE27786_NKTCELL_VS_NEUTROPHIL_UP |
| c7.all.v5.0.entrez.gmt.msig.set | 0.00005 | 0.5 | 0.0911818* | 188 | 12 | 0.00009999 | GSE27786_NKTCELL_VS_NEUTROPHIL_UP http://www.broadinstitute.org/gsea/msigdb/cards/GSE27786_NKTCELL_VS_NEUTROPHIL_UP |
| ***SSRI responders vs. healthy controls*** | | | | | | | |
| c2.cgp.v5.0.entrez.gmt.msig.set | 0.00005 | 0.2 | 0.0967806* | 51 | 7 | 0.00009999 | KENNY_CTNNB1_TARGETS_DN http://www.broadinstitute.org/gsea/msigdb/cards/KENNY_CTNNB1_TARGETS_DN |
| c2.cgp.v5.0.entrez.gmt.msig.set | 0.00005 | 0.3 | 0.0993801* | 51 | 7 | 0.00009999 | KENNY_CTNNB1_TARGETS_DN http://www.broadinstitute.org/gsea/msigdb/cards/KENNY_CTNNB1_TARGETS_DN |
| c2.cp.kegg.v5.0.entrez.gmt.msig.set | 0.00005 | 0.3 | 0.0625875 | 24 | 4 | 0.00109989 | KEGG_NICOTINATE_AND_NICOTINAMIDE_METABOLISM http://www.broadinstitute.org/gsea/msigdb/cards/KEGG_NICOTINATE_AND_NICOTINAMIDE_METABOLISM |
| c2.cp.kegg.v5.0.entrez.gmt.msig.set | 0.00005 | 0.5 | 0.0945811 | 24 | 4 | 0.00219978 | KEGG_NICOTINATE_AND_NICOTINAMIDE_METABOLISM http://www.broadinstitute.org/gsea/msigdb/cards/KEGG_NICOTINATE_AND_NICOTINAMIDE_METABOLISM |
| c2.cp.reactome.v5.0.entrez.gmt.msig.set | 0.0005 | 0.2 | 0.0389922 | 26 | 6 | 0.00019998 | REACTOME_ENDOSOMAL_SORTING_COMPLEX_REQUIRED_FOR_TRANSPORT_ESCRT http://www.broadinstitute.org/gsea/msigdb/cards/REACTOME_ENDOSOMAL_SORTING_COMPLEX_REQUIRED_FOR_TRANSPORT_ESCRT |
| c2.cp.reactome.v5.0.entrez.gmt.msig.set | 0.0005 | 0.2 | 0.0537892 | 30 | 7 | 0.00029997 | REACTOME_INTERACTIONS_OF_VPR_WITH_HOST_CELLULAR_PROTEINS http://www.broadinstitute.org/gsea/msigdb/cards/REACTOME_INTERACTIONS_OF_VPR_WITH_HOST_CELLULAR_PROTEINS |
| c2.cp.reactome.v5.0.entrez.gmt.msig.set | 0.0005 | 0.2 | 0.0875825 | 27 | 6 | 0.00059994 | REACTOME_NEP_NS2_INTERACTS_WITH_THE_CELLULAR_EXPORT_MACHINERY http://www.broadinstitute.org/gsea/msigdb/cards/REACTOME_NEP_NS2_INTERACTS_WITH_THE_CELLULAR_EXPORT_MACHINERY |
| c2.cp.reactome.v5.0.entrez.gmt.msig.set | 0.0005 | 0.2 | 0.0875825 | 27 | 6 | 0.00059994 | REACTOME_REGULATION_OF_GLUCOKINASE_BY_GLUCOKINASE_REGULATORY_PROTEIN http://www.broadinstitute.org/gsea/msigdb/cards/REACTOME_REGULATION_OF_GLUCOKINASE_BY_GLUCOKINASE_REGULATORY_PROTEIN |
| c2.cp.reactome.v5.0.entrez.gmt.msig.set | 0.0005 | 0.3 | 0.0257948* | 26 | 6 | 0.00009999 | REACTOME_ENDOSOMAL_SORTING_COMPLEX_REQUIRED_FOR_TRANSPORT_ESCRT http://www.broadinstitute.org/gsea/msigdb/cards/REACTOME_ENDOSOMAL_SORTING_COMPLEX_REQUIRED_FOR_TRANSPORT_ESCRT |
| c2.cp.reactome.v5.0.entrez.gmt.msig.set | 0.0005 | 0.3 | 0.0519896 | 30 | 7 | 0.00029997 | REACTOME_INTERACTIONS_OF_VPR_WITH_HOST_CELLULAR_PROTEINS http://www.broadinstitute.org/gsea/msigdb/cards/REACTOME_INTERACTIONS_OF_VPR_WITH_HOST_CELLULAR_PROTEINS |
| c2.cp.reactome.v5.0.entrez.gmt.msig.set | 0.0005 | 0.5 | 0.0643871 | 26 | 5 | 0.00029997 | REACTOME_ENDOSOMAL_SORTING_COMPLEX_REQUIRED_FOR_TRANSPORT_ESCRT http://www.broadinstitute.org/gsea/msigdb/cards/REACTOME_ENDOSOMAL_SORTING_COMPLEX_REQUIRED_FOR_TRANSPORT_ESCRT |
| c2.cp.reactome.v5.0.entrez.gmt.msig.set | 0.0005 | 0.5 | 0.0743851 | 30 | 7 | 0.00039996 | REACTOME_INTERACTIONS_OF_VPR_WITH_HOST_CELLULAR_PROTEINS http://www.broadinstitute.org/gsea/msigdb/cards/REACTOME_INTERACTIONS_OF_VPR_WITH_HOST_CELLULAR_PROTEINS |
| c2.cp.reactome.v5.0.entrez.gmt.msig.set | 0.0005 | 0.5 | 0.0643871 | 27 | 6 | 0.00029997 | REACTOME_NEP_NS2_INTERACTS_WITH_THE_CELLULAR_EXPORT_MACHINERY http://www.broadinstitute.org/gsea/msigdb/cards/REACTOME_NEP_NS2_INTERACTS_WITH_THE_CELLULAR_EXPORT_MACHINERY |
| c2.cp.reactome.v5.0.entrez.gmt.msig.set | 0.0005 | 0.5 | 0.0973805 | 27 | 6 | 0.00059994 | REACTOME_REGULATION_OF_GLUCOKINASE_BY_GLUCOKINASE_REGULATORY_PROTEIN http://www.broadinstitute.org/gsea/msigdb/cards/REACTOME_REGULATION_OF_GLUCOKINASE_BY_GLUCOKINASE_REGULATORY_PROTEIN |
| c2.cp.v5.0.entrez.gmt.msig.set | 0.0005 | 0.2 | 0.0785843 | 27 | 7 | 0.00019998 | PID_TRAIL_PATHWAY http://www.broadinstitute.org/gsea/msigdb/cards/PID_TRAIL_PATHWAY |
| c2.cp.v5.0.entrez.gmt.msig.set | 0.0005 | 0.2 | 0.0539892* | 26 | 6 | 0.00009999 | REACTOME_ENDOSOMAL_SORTING_COMPLEX_REQUIRED_FOR_TRANSPORT_ESCRT http://www.broadinstitute.org/gsea/msigdb/cards/REACTOME_ENDOSOMAL_SORTING_COMPLEX_REQUIRED_FOR_TRANSPORT_ESCRT |
| c2.cp.v5.0.entrez.gmt.msig.set | 0.0005 | 0.3 | 0.0593881* | 27 | 7 | 0.00009999 | PID_TRAIL_PATHWAY http://www.broadinstitute.org/gsea/msigdb/cards/PID_TRAIL_PATHWAY |
| c2.cp.v5.0.entrez.gmt.msig.set | 0.0005 | 0.3 | 0.0593881* | 26 | 6 | 0.00009999 | REACTOME_ENDOSOMAL_SORTING_COMPLEX_REQUIRED_FOR_TRANSPORT_ESCRT http://www.broadinstitute.org/gsea/msigdb/cards/REACTOME_ENDOSOMAL_SORTING_COMPLEX_REQUIRED_FOR_TRANSPORT_ESCRT |
| c2.cp.v5.0.entrez.gmt.msig.set | 0.0005 | 0.5 | 0.0857828 | 27 | 6 | 0.00019998 | PID_TRAIL_PATHWAY http://www.broadinstitute.org/gsea/msigdb/cards/PID_TRAIL_PATHWAY |
| c1.all.v5.0.entrez.gmt.msig.set | 0.0005 | 0.2 | 0.0445911 | 88 | 6 | 0.00039996 | chr2q33 http://www.broadinstitute.org/gsea/msigdb/cards/chr2q33 |
| c1.all.v5.0.entrez.gmt.msig.set | 0.0005 | 0.3 | 0.0627874 | 88 | 6 | 0.00059994 | chr2q33 http://www.broadinstitute.org/gsea/msigdb/cards/chr2q33 |
| c1.all.v5.0.entrez.gmt.msig.set | 0.0005 | 0.5 | 0.0341932 | 88 | 6 | 0.00029997 | chr2q33 http://www.broadinstitute.org/gsea/msigdb/cards/chr2q33 |
| c5.all.v5.0.entrez.gmt.msig.set | 0.0005 | 0.2 | 0.0629874* | 11 | 5 | 0.00009999 | NUCLEAR_MATRIX http://www.broadinstitute.org/gsea/msigdb/cards/NUCLEAR_MATRIX |
| c5.all.v5.0.entrez.gmt.msig.set | 0.0005 | 0.3 | 0.0589882* | 11 | 5 | 0.00009999 | NUCLEAR_MATRIX http://www.broadinstitute.org/gsea/msigdb/cards/NUCLEAR_MATRIX |
| c5.all.v5.0.entrez.gmt.msig.set | 0.0005 | 0.5 | 0.0633873* | 11 | 5 | 0.00009999 | NUCLEAR_MATRIX http://www.broadinstitute.org/gsea/msigdb/cards/NUCLEAR_MATRIX |
| c5.cc.v5.0.entrez.gmt.msig.set | 0.0005 | 0.3 | 0.0775845 | 11 | 4 | 0.00139986 | NUCLEAR_MATRIX http://www.broadinstitute.org/gsea/msigdb/cards/NUCLEAR_MATRIX |
| ***citalopram or escitalopram non-responders vs. healthy controls*** | | | | | | | |
| go.set | 0.00001 | 0.2 | 0.0505899 | 191 | 3 | 0.00129987 | GO:0007264 small |
| c2.cp.reactome.v5.0.entrez.gmt.msig.set | 0.0005 | 0.2 | 0.0919816 | 14 | 3 | 0.00069993 | REACTOME_VIRAL_MESSENGER_RNA_SYNTHESIS http://www.broadinstitute.org/gsea/msigdb/cards/REACTOME_VIRAL_MESSENGER_RNA_SYNTHESIS |
| c2.cp.reactome.v5.0.entrez.gmt.msig.set | 0.0005 | 0.5 | 0.0889822 | 14 | 3 | 0.00069993 | REACTOME_VIRAL_MESSENGER_RNA_SYNTHESIS http://www.broadinstitute.org/gsea/msigdb/cards/REACTOME_VIRAL_MESSENGER_RNA_SYNTHESIS |
| c1.all.v5.0.entrez.gmt.msig.set | 0.00005 | 0.3 | 0.0987802 | 141 | 3 | 0.0029997 | chr1q32 http://www.broadinstitute.org/gsea/msigdb/cards/chr1q32 |
| c1.all.v5.0.entrez.gmt.msig.set | 0.0005 | 0.2 | 0.0487902 | 23 | 4 | 0.00039996 | chr8q23 http://www.broadinstitute.org/gsea/msigdb/cards/chr8q23 |
| c3.all.v5.0.entrez.gmt.msig.set | 0.0005 | 0.2 | 0.0457908 | 81 | 14 | 0.00019998 | ATGTCAC,MIR-489 http://www.broadinstitute.org/gsea/msigdb/cards/ATGTCAC,MIR-489 |
| c3.all.v5.0.entrez.gmt.msig.set | 0.0005 | 0.2 | 0.0457908 | 65 | 11 | 0.00019998 | WYAAANNRNNNGCG_UNKNOWN http://www.broadinstitute.org/gsea/msigdb/cards/WYAAANNRNNNGCG_UNKNOWN |
| c3.all.v5.0.entrez.gmt.msig.set | 0.0005 | 0.5 | 0.0817836 | 81 | 13 | 0.00049995 | ATGTCAC,MIR-489 http://www.broadinstitute.org/gsea/msigdb/cards/ATGTCAC,MIR-489 |
| c3.all.v5.0.entrez.gmt.msig.set | 0.0005 | 0.5 | 0.0915817 | 65 | 10 | 0.00059994 | WYAAANNRNNNGCG_UNKNOWN http://www.broadinstitute.org/gsea/msigdb/cards/WYAAANNRNNNGCG_UNKNOWN |
| c3.mir.v5.0.entrez.gmt.msig.set | 0.0005 | 0.2 | 0.0503899 | 81 | 14 | 0.00049995 | ATGTCAC,MIR-489 http://www.broadinstitute.org/gsea/msigdb/cards/ATGTCAC,MIR-489 |
| c3.mir.v5.0.entrez.gmt.msig.set | 0.0005 | 0.3 | 0.0163967* | 81 | 14 | 0.00009999 | ATGTCAC,MIR-489 http://www.broadinstitute.org/gsea/msigdb/cards/ATGTCAC,MIR-489 |
| c3.mir.v5.0.entrez.gmt.msig.set | 0.0005 | 0.5 | 0.0421916 | 81 | 13 | 0.00049995 | ATGTCAC,MIR-489 http://www.broadinstitute.org/gsea/msigdb/cards/ATGTCAC,MIR-489 |
| c3.tft.v5.0.entrez.gmt.msig.set | 0.0005 | 0.5 | 0.0463907 | 65 | 9 | 0.00049995 | WYAAANNRNNNGCG_UNKNOWN http://www.broadinstitute.org/gsea/msigdb/cards/WYAAANNRNNNGCG_UNKNOWN |
| c5.all.v5.0.entrez.gmt.msig.set | 0.00001 | 0.2 | 0.0705859 | 120 | 3 | 0.00459954 | NEGATIVE_REGULATION_OF_RNA_METABOLIC_PROCESS http://www.broadinstitute.org/gsea/msigdb/cards/NEGATIVE_REGULATION_OF_RNA_METABOLIC_PROCESS |
| c5.all.v5.0.entrez.gmt.msig.set | 0.00001 | 0.2 | 0.0577884 | 174 | 3 | 0.00349965 | NEGATIVE_REGULATION_OF_TRANSCRIPTION http://www.broadinstitute.org/gsea/msigdb/cards/NEGATIVE_REGULATION_OF_TRANSCRIPTION |
| c5.all.v5.0.entrez.gmt.msig.set | 0.00001 | 0.2 | 0.0529894 | 120 | 3 | 0.00269973 | NEGATIVE_REGULATION_OF_TRANSCRIPTION_DNA_DEPENDENT http://www.broadinstitute.org/gsea/msigdb/cards/NEGATIVE_REGULATION_OF_TRANSCRIPTION_DNA_DEPENDENT |
| c5.all.v5.0.entrez.gmt.msig.set | 0.00001 | 0.3 | 0.0229954 | 120 | 3 | 0.00189981 | NEGATIVE_REGULATION_OF_RNA_METABOLIC_PROCESS http://www.broadinstitute.org/gsea/msigdb/cards/NEGATIVE_REGULATION_OF_RNA_METABOLIC_PROCESS |
| c5.all.v5.0.entrez.gmt.msig.set | 0.00001 | 0.3 | 0.0209958 | 174 | 3 | 0.00159984 | NEGATIVE_REGULATION_OF_TRANSCRIPTION http://www.broadinstitute.org/gsea/msigdb/cards/NEGATIVE_REGULATION_OF_TRANSCRIPTION |
| c5.all.v5.0.entrez.gmt.msig.set | 0.00001 | 0.3 | 0.0163967 | 120 | 3 | 0.0009999 | NEGATIVE_REGULATION_OF_TRANSCRIPTION_DNA_DEPENDENT http://www.broadinstitute.org/gsea/msigdb/cards/NEGATIVE_REGULATION_OF_TRANSCRIPTION_DNA_DEPENDENT |
| c5.all.v5.0.entrez.gmt.msig.set | 0.00001 | 0.5 | 0.0195961 | 120 | 3 | 0.00189981 | NEGATIVE_REGULATION_OF_RNA_METABOLIC_PROCESS http://www.broadinstitute.org/gsea/msigdb/cards/NEGATIVE_REGULATION_OF_RNA_METABOLIC_PROCESS |
| c5.all.v5.0.entrez.gmt.msig.set | 0.00001 | 0.5 | 0.0171966 | 174 | 3 | 0.00129987 | NEGATIVE_REGULATION_OF_TRANSCRIPTION http://www.broadinstitute.org/gsea/msigdb/cards/NEGATIVE_REGULATION_OF_TRANSCRIPTION |
| c5.all.v5.0.entrez.gmt.msig.set | 0.00001 | 0.5 | 0.0153969 | 120 | 3 | 0.0009999 | NEGATIVE_REGULATION_OF_TRANSCRIPTION_DNA_DEPENDENT http://www.broadinstitute.org/gsea/msigdb/cards/NEGATIVE_REGULATION_OF_TRANSCRIPTION_DNA_DEPENDENT |
| c5.bp.v5.0.entrez.gmt.msig.set | 0.00001 | 0.2 | 0.0373925 | 120 | 3 | 0.00479952 | NEGATIVE_REGULATION_OF_RNA_METABOLIC_PROCESS http://www.broadinstitute.org/gsea/msigdb/cards/NEGATIVE_REGULATION_OF_RNA_METABOLIC_PROCESS |
| c5.bp.v5.0.entrez.gmt.msig.set | 0.00001 | 0.2 | 0.0343931 | 174 | 3 | 0.00389961 | NEGATIVE_REGULATION_OF_TRANSCRIPTION http://www.broadinstitute.org/gsea/msigdb/cards/NEGATIVE_REGULATION_OF_TRANSCRIPTION |
| c5.bp.v5.0.entrez.gmt.msig.set | 0.00001 | 0.2 | 0.029994 | 120 | 3 | 0.00269973 | NEGATIVE_REGULATION_OF_TRANSCRIPTION_DNA_DEPENDENT http://www.broadinstitute.org/gsea/msigdb/cards/NEGATIVE_REGULATION_OF_TRANSCRIPTION_DNA_DEPENDENT |
| c5.bp.v5.0.entrez.gmt.msig.set | 0.00001 | 0.3 | 0.0137972 | 120 | 3 | 0.00179982 | NEGATIVE_REGULATION_OF_RNA_METABOLIC_PROCESS http://www.broadinstitute.org/gsea/msigdb/cards/NEGATIVE_REGULATION_OF_RNA_METABOLIC_PROCESS |
| c5.bp.v5.0.entrez.gmt.msig.set | 0.00001 | 0.3 | 0.0113977 | 174 | 3 | 0.00089991 | NEGATIVE_REGULATION_OF_TRANSCRIPTION http://www.broadinstitute.org/gsea/msigdb/cards/NEGATIVE_REGULATION_OF_TRANSCRIPTION |
| c5.bp.v5.0.entrez.gmt.msig.set | 0.00001 | 0.3 | 0.0079984 | 120 | 3 | 0.00059994 | NEGATIVE_REGULATION_OF_TRANSCRIPTION_DNA_DEPENDENT http://www.broadinstitute.org/gsea/msigdb/cards/NEGATIVE_REGULATION_OF_TRANSCRIPTION_DNA_DEPENDENT |
| c5.bp.v5.0.entrez.gmt.msig.set | 0.00001 | 0.5 | 0.00919816 | 120 | 3 | 0.00149985 | NEGATIVE_REGULATION_OF_RNA_METABOLIC_PROCESS http://www.broadinstitute.org/gsea/msigdb/cards/NEGATIVE_REGULATION_OF_RNA_METABOLIC_PROCESS |
| c5.bp.v5.0.entrez.gmt.msig.set | 0.00001 | 0.5 | 0.00939812 | 174 | 3 | 0.00179982 | NEGATIVE_REGULATION_OF_TRANSCRIPTION http://www.broadinstitute.org/gsea/msigdb/cards/NEGATIVE_REGULATION_OF_TRANSCRIPTION |
| c5.bp.v5.0.entrez.gmt.msig.set | 0.00001 | 0.5 | 0.00839832 | 120 | 3 | 0.0009999 | NEGATIVE_REGULATION_OF_TRANSCRIPTION_DNA_DEPENDENT http://www.broadinstitute.org/gsea/msigdb/cards/NEGATIVE_REGULATION_OF_TRANSCRIPTION_DNA_DEPENDENT |
| c7.all.v5.0.entrez.gmt.msig.set | 0.00001 | 0.3 | 0.0779844 | 193 | 3 | 0.00029997 | GSE27786_BCELL_VS_NEUTROPHIL_DN http://www.broadinstitute.org/gsea/msigdb/cards/GSE27786_BCELL_VS_NEUTROPHIL_DN |
| c7.all.v5.0.entrez.gmt.msig.set | 0.00001 | 0.3 | 0.0927814 | 182 | 3 | 0.00039996 | GSE360_CTRL_VS_L_MAJOR_MAC_DN http://www.broadinstitute.org/gsea/msigdb/cards/GSE360_CTRL_VS_L_MAJOR_MAC_DN |
| c7.all.v5.0.entrez.gmt.msig.set | 0.00001 | 0.5 | 0.0895821 | 180 | 3 | 0.00039996 | GSE13411_PLASMA_CELL_VS_MEMORY_BCELL_DN http://www.broadinstitute.org/gsea/msigdb/cards/GSE13411_PLASMA_CELL_VS_MEMORY_BCELL_DN |
| c7.all.v5.0.entrez.gmt.msig.set | 0.00001 | 0.5 | 0.0479904* | 193 | 3 | 0.00009999 | GSE27786_BCELL_VS_NEUTROPHIL_DN http://www.broadinstitute.org/gsea/msigdb/cards/GSE27786_BCELL_VS_NEUTROPHIL_DN |
| c7.all.v5.0.entrez.gmt.msig.set | 0.00001 | 0.5 | 0.0895821 | 182 | 3 | 0.00039996 | GSE360_CTRL_VS_L_MAJOR_MAC_DN http://www.broadinstitute.org/gsea/msigdb/cards/GSE360_CTRL_VS_L_MAJOR_MAC_DN |
| c7.all.v5.0.entrez.gmt.msig.set | 0.00005 | 0.5 | 0.0789842* | 192 | 12 | 0.00009999 | GSE3982_BASOPHIL_VS_NKCELL_UP http://www.broadinstitute.org/gsea/msigdb/cards/GSE3982_BASOPHIL_VS_NKCELL_UP |
| ***citalopram or escitalopram responders vs. healthy controls*** | | | | | | | |
| kegg.set | 0.0005 | 0.3 | 0.0303939 | 7 | 4 | 0.00049995 | 00460 Cyanoamino_acid_metabolism |
| kegg.set | 0.0005 | 0.5 | 0.0509898 | 7 | 4 | 0.00059994 | 00460 Cyanoamino_acid_metabolism |
| c2.all.v5.0.entrez.gmt.msig.set | 0.00001 | 0.2 | 0.089782 | 192 | 4 | 0.00029997 | REACTOME_GASTRIN_CREB_SIGNALLING_PATHWAY_VIA_PKC_AND_MAPK http://www.broadinstitute.org/gsea/msigdb/cards/REACTOME_GASTRIN_CREB_SIGNALLING_PATHWAY_VIA_PKC_AND_MAPK |
| c2.all.v5.0.entrez.gmt.msig.set | 0.00001 | 0.2 | 0.089782 | 172 | 4 | 0.00029997 | REACTOME_G_ALPHA_Q_SIGNALLING_EVENTS http://www.broadinstitute.org/gsea/msigdb/cards/REACTOME_G_ALPHA_Q_SIGNALLING_EVENTS |
| c2.all.v5.0.entrez.gmt.msig.set | 0.00001 | 0.2 | 0.0467906* | 84 | 4 | 0.00009999 | SCHLESINGER_METHYLATED_DE_NOVO_IN_CANCER http://www.broadinstitute.org/gsea/msigdb/cards/SCHLESINGER_METHYLATED_DE_NOVO_IN_CANCER |
| c2.all.v5.0.entrez.gmt.msig.set | 0.00001 | 0.3 | 0.0891822 | 192 | 4 | 0.00029997 | REACTOME_GASTRIN_CREB_SIGNALLING_PATHWAY_VIA_PKC_AND_MAPK http://www.broadinstitute.org/gsea/msigdb/cards/REACTOME_GASTRIN_CREB_SIGNALLING_PATHWAY_VIA_PKC_AND_MAPK |
| c2.all.v5.0.entrez.gmt.msig.set | 0.00001 | 0.3 | 0.0693861 | 172 | 4 | 0.00019998 | REACTOME_G_ALPHA_Q_SIGNALLING_EVENTS http://www.broadinstitute.org/gsea/msigdb/cards/REACTOME_G_ALPHA_Q_SIGNALLING_EVENTS |
| c2.all.v5.0.entrez.gmt.msig.set | 0.00001 | 0.3 | 0.0455909* | 84 | 4 | 0.00009999 | SCHLESINGER_METHYLATED_DE_NOVO_IN_CANCER http://www.broadinstitute.org/gsea/msigdb/cards/SCHLESINGER_METHYLATED_DE_NOVO_IN_CANCER |
| c2.all.v5.0.entrez.gmt.msig.set | 0.00001 | 0.5 | 0.0485903* | 84 | 4 | 0.00009999 | SCHLESINGER_METHYLATED_DE_NOVO_IN_CANCER http://www.broadinstitute.org/gsea/msigdb/cards/SCHLESINGER_METHYLATED_DE_NOVO_IN_CANCER |
| c2.cgp.v5.0.entrez.gmt.msig.set | 0.00001 | 0.2 | 0.0657868 | 84 | 4 | 0.00019998 | SCHLESINGER_METHYLATED_DE_NOVO_IN_CANCER http://www.broadinstitute.org/gsea/msigdb/cards/SCHLESINGER_METHYLATED_DE_NOVO_IN_CANCER |
| c2.cgp.v5.0.entrez.gmt.msig.set | 0.00001 | 0.3 | 0.0389922* | 84 | 4 | 0.00009999 | SCHLESINGER_METHYLATED_DE_NOVO_IN_CANCER http://www.broadinstitute.org/gsea/msigdb/cards/SCHLESINGER_METHYLATED_DE_NOVO_IN_CANCER |
| c2.cgp.v5.0.entrez.gmt.msig.set | 0.00001 | 0.5 | 0.044991* | 84 | 4 | 0.00009999 | SCHLESINGER_METHYLATED_DE_NOVO_IN_CANCER http://www.broadinstitute.org/gsea/msigdb/cards/SCHLESINGER_METHYLATED_DE_NOVO_IN_CANCER |
| c2.cgp.v5.0.entrez.gmt.msig.set | 0.00005 | 0.3 | 0.0983803* | 84 | 8 | 0.00009999 | SCHLESINGER_METHYLATED_DE_NOVO_IN_CANCER http://www.broadinstitute.org/gsea/msigdb/cards/SCHLESINGER_METHYLATED_DE_NOVO_IN_CANCER |
| c2.cgp.v5.0.entrez.gmt.msig.set | 0.00005 | 0.5 | 0.0961808* | 84 | 8 | 0.00009999 | SCHLESINGER_METHYLATED_DE_NOVO_IN_CANCER http://www.broadinstitute.org/gsea/msigdb/cards/SCHLESINGER_METHYLATED_DE_NOVO_IN_CANCER |
| c2.cp.reactome.v5.0.entrez.gmt.msig.set | 0.00001 | 0.2 | 0.0109978 | 192 | 3 | 0.00049995 | REACTOME_GASTRIN_CREB_SIGNALLING_PATHWAY_VIA_PKC_AND_MAPK http://www.broadinstitute.org/gsea/msigdb/cards/REACTOME_GASTRIN_CREB_SIGNALLING_PATHWAY_VIA_PKC_AND_MAPK |
| c2.cp.reactome.v5.0.entrez.gmt.msig.set | 0.00001 | 0.2 | 0.00939812 | 172 | 3 | 0.00039996 | REACTOME_G_ALPHA_Q_SIGNALLING_EVENTS http://www.broadinstitute.org/gsea/msigdb/cards/REACTOME_G_ALPHA_Q_SIGNALLING_EVENTS |
| c2.cp.reactome.v5.0.entrez.gmt.msig.set | 0.00001 | 0.3 | 0.00679864 | 192 | 3 | 0.00039996 | REACTOME_GASTRIN_CREB_SIGNALLING_PATHWAY_VIA_PKC_AND_MAPK http://www.broadinstitute.org/gsea/msigdb/cards/REACTOME_GASTRIN_CREB_SIGNALLING_PATHWAY_VIA_PKC_AND_MAPK |
| c2.cp.reactome.v5.0.entrez.gmt.msig.set | 0.00001 | 0.3 | 0.004999 | 172 | 3 | 0.00029997 | REACTOME_G_ALPHA_Q_SIGNALLING_EVENTS http://www.broadinstitute.org/gsea/msigdb/cards/REACTOME_G_ALPHA_Q_SIGNALLING_EVENTS |
| c2.cp.reactome.v5.0.entrez.gmt.msig.set | 0.00005 | 0.2 | 0.0741852 | 13 | 3 | 0.00059994 | REACTOME_NUCLEOTIDE_LIKE_PURINERGIC_RECEPTORS http://www.broadinstitute.org/gsea/msigdb/cards/REACTOME_NUCLEOTIDE_LIKE_PURINERGIC_RECEPTORS |
| c2.cp.reactome.v5.0.entrez.gmt.msig.set | 0.0001 | 0.3 | 0.0915817 | 9 | 3 | 0.00069993 | REACTOME_IKK_COMPLEX_RECRUITMENT_MEDIATED_BY_RIP1 http://www.broadinstitute.org/gsea/msigdb/cards/REACTOME_IKK_COMPLEX_RECRUITMENT_MEDIATED_BY_RIP1 |
| c2.cp.reactome.v5.0.entrez.gmt.msig.set | 0.0005 | 0.3 | 0.0671866 | 10 | 4 | 0.00039996 | REACTOME_COPI_MEDIATED_TRANSPORT http://www.broadinstitute.org/gsea/msigdb/cards/REACTOME_COPI_MEDIATED_TRANSPORT |
| c2.cp.reactome.v5.0.entrez.gmt.msig.set | 0.0005 | 0.3 | 0.0317936* | 13 | 4 | 0.00009999 | REACTOME_NUCLEOTIDE_LIKE_PURINERGIC_RECEPTORS http://www.broadinstitute.org/gsea/msigdb/cards/REACTOME_NUCLEOTIDE_LIKE_PURINERGIC_RECEPTORS |
| c2.cp.reactome.v5.0.entrez.gmt.msig.set | 0.0005 | 0.5 | 0.0683863 | 10 | 4 | 0.00039996 | REACTOME_COPI_MEDIATED_TRANSPORT http://www.broadinstitute.org/gsea/msigdb/cards/REACTOME_COPI_MEDIATED_TRANSPORT |
| c2.cp.reactome.v5.0.entrez.gmt.msig.set | 0.0005 | 0.5 | 0.0265947* | 13 | 4 | 0.00009999 | REACTOME_NUCLEOTIDE_LIKE_PURINERGIC_RECEPTORS http://www.broadinstitute.org/gsea/msigdb/cards/REACTOME_NUCLEOTIDE_LIKE_PURINERGIC_RECEPTORS |
| c2.cp.v5.0.entrez.gmt.msig.set | 0.00001 | 0.2 | 0.030194 | 192 | 3 | 0.00049995 | REACTOME_GASTRIN_CREB_SIGNALLING_PATHWAY_VIA_PKC_AND_MAPK http://www.broadinstitute.org/gsea/msigdb/cards/REACTOME_GASTRIN_CREB_SIGNALLING_PATHWAY_VIA_PKC_AND_MAPK |
| c2.cp.v5.0.entrez.gmt.msig.set | 0.00001 | 0.2 | 0.0277944 | 172 | 3 | 0.00039996 | REACTOME_G_ALPHA_Q_SIGNALLING_EVENTS http://www.broadinstitute.org/gsea/msigdb/cards/REACTOME_G_ALPHA_Q_SIGNALLING_EVENTS |
| c2.cp.v5.0.entrez.gmt.msig.set | 0.00001 | 0.3 | 0.0171966 | 192 | 3 | 0.00039996 | REACTOME_GASTRIN_CREB_SIGNALLING_PATHWAY_VIA_PKC_AND_MAPK http://www.broadinstitute.org/gsea/msigdb/cards/REACTOME_GASTRIN_CREB_SIGNALLING_PATHWAY_VIA_PKC_AND_MAPK |
| c2.cp.v5.0.entrez.gmt.msig.set | 0.00001 | 0.3 | 0.014797 | 172 | 3 | 0.00029997 | REACTOME_G_ALPHA_Q_SIGNALLING_EVENTS http://www.broadinstitute.org/gsea/msigdb/cards/REACTOME_G_ALPHA_Q_SIGNALLING_EVENTS |
| c2.cp.v5.0.entrez.gmt.msig.set | 0.00005 | 0.3 | 0.0777844 | 33 | 5 | 0.00029997 | PID_EPO_PATHWAY http://www.broadinstitute.org/gsea/msigdb/cards/PID_EPO_PATHWAY |
| c2.cp.v5.0.entrez.gmt.msig.set | 0.00005 | 0.5 | 0.0615877 | 33 | 5 | 0.00019998 | PID_EPO_PATHWAY http://www.broadinstitute.org/gsea/msigdb/cards/PID_EPO_PATHWAY |
| c2.cp.v5.0.entrez.gmt.msig.set | 0.0005 | 0.2 | 0.055189* | 13 | 4 | 0.00009999 | REACTOME_NUCLEOTIDE_LIKE_PURINERGIC_RECEPTORS http://www.broadinstitute.org/gsea/msigdb/cards/REACTOME_NUCLEOTIDE_LIKE_PURINERGIC_RECEPTORS |
| c2.cp.v5.0.entrez.gmt.msig.set | 0.0005 | 0.3 | 0.0585883* | 13 | 4 | 0.00009999 | REACTOME_NUCLEOTIDE_LIKE_PURINERGIC_RECEPTORS http://www.broadinstitute.org/gsea/msigdb/cards/REACTOME_NUCLEOTIDE_LIKE_PURINERGIC_RECEPTORS |
| c2.cp.v5.0.entrez.gmt.msig.set | 0.0005 | 0.5 | 0.0587882* | 13 | 4 | 0.00009999 | REACTOME_NUCLEOTIDE_LIKE_PURINERGIC_RECEPTORS http://www.broadinstitute.org/gsea/msigdb/cards/REACTOME_NUCLEOTIDE_LIKE_PURINERGIC_RECEPTORS |
| c1.all.v5.0.entrez.gmt.msig.set | 0.0005 | 0.2 | 0.035193 | 54 | 3 | 0.00029997 | chr2p11 http://www.broadinstitute.org/gsea/msigdb/cards/chr2p11 |
| c1.all.v5.0.entrez.gmt.msig.set | 0.0005 | 0.2 | 0.0207958* | 105 | 7 | 0.00009999 | chr7q22 http://www.broadinstitute.org/gsea/msigdb/cards/chr7q22 |
| c1.all.v5.0.entrez.gmt.msig.set | 0.0005 | 0.3 | 0.0283943 | 54 | 3 | 0.00019998 | chr2p11 http://www.broadinstitute.org/gsea/msigdb/cards/chr2p11 |
| c1.all.v5.0.entrez.gmt.msig.set | 0.0005 | 0.3 | 0.0539892 | 105 | 7 | 0.00049995 | chr7q22 http://www.broadinstitute.org/gsea/msigdb/cards/chr7q22 |
| c1.all.v5.0.entrez.gmt.msig.set | 0.0005 | 0.5 | 0.0767846 | 113 | 5 | 0.00069993 | chr11q23 http://www.broadinstitute.org/gsea/msigdb/cards/chr11q23 |
| c1.all.v5.0.entrez.gmt.msig.set | 0.0005 | 0.5 | 0.0367926 | 105 | 7 | 0.00029997 | chr7q22 http://www.broadinstitute.org/gsea/msigdb/cards/chr7q22 |
| c3.tft.v5.0.entrez.gmt.msig.set | 0.0005 | 0.3 | 0.029794 | 42 | 8 | 0.00019998 | V$MYOGNF1_01 http://www.broadinstitute.org/gsea/msigdb/cards/V$MYOGNF1_01 |
| c3.tft.v5.0.entrez.gmt.msig.set | 0.0005 | 0.5 | 0.0181964* | 42 | 8 | 0.00009999 | V$MYOGNF1_01 http://www.broadinstitute.org/gsea/msigdb/cards/V$MYOGNF1_01 |
| c4.cm.v5.0.entrez.gmt.msig.set | 0.0005 | 0.3 | 0.0437912 | 27 | 8 | 0.00039996 | MODULE_200 http://www.broadinstitute.org/gsea/msigdb/cards/MODULE_200 |
| c4.cm.v5.0.entrez.gmt.msig.set | 0.0005 | 0.3 | 0.0763847 | 36 | 7 | 0.00079992 | MODULE_51 http://www.broadinstitute.org/gsea/msigdb/cards/MODULE_51 |
| c4.cm.v5.0.entrez.gmt.msig.set | 0.0005 | 0.3 | 0.0437912 | 32 | 7 | 0.00039996 | MODULE_80 http://www.broadinstitute.org/gsea/msigdb/cards/MODULE_80 |
| c4.cm.v5.0.entrez.gmt.msig.set | 0.0005 | 0.5 | 0.0757848 | 32 | 7 | 0.00069993 | MODULE_80 http://www.broadinstitute.org/gsea/msigdb/cards/MODULE_80 |
| c5.all.v5.0.entrez.gmt.msig.set | 0.0005 | 0.5 | 0.0779844 | 85 | 10 | 0.00019998 | POSITIVE_REGULATION_OF_I_KAPPAB_KINASE_NF_KAPPAB_CASCADE http://www.broadinstitute.org/gsea/msigdb/cards/POSITIVE_REGULATION_OF_I_KAPPAB_KINASE_NF_KAPPAB_CASCADE |
| c5.bp.v5.0.entrez.gmt.msig.set | 0.0005 | 0.2 | 0.0933813 | 109 | 11 | 0.00049995 | I_KAPPAB_KINASE_NF_KAPPAB_CASCADE http://www.broadinstitute.org/gsea/msigdb/cards/I_KAPPAB_KINASE_NF_KAPPAB_CASCADE |
| c5.bp.v5.0.entrez.gmt.msig.set | 0.0005 | 0.3 | 0.0771846 | 52 | 9 | 0.00039996 | REGULATION_OF_BODY_FLUID_LEVELS http://www.broadinstitute.org/gsea/msigdb/cards/REGULATION_OF_BODY_FLUID_LEVELS |
| c5.bp.v5.0.entrez.gmt.msig.set | 0.0005 | 0.5 | 0.0461908 | 85 | 10 | 0.00019998 | POSITIVE_REGULATION_OF_I_KAPPAB_KINASE_NF_KAPPAB_CASCADE http://www.broadinstitute.org/gsea/msigdb/cards/POSITIVE_REGULATION_OF_I_KAPPAB_KINASE_NF_KAPPAB_CASCADE |
| c5.bp.v5.0.entrez.gmt.msig.set | 0.0005 | 0.5 | 0.0461908 | 52 | 9 | 0.00019998 | REGULATION_OF_BODY_FLUID_LEVELS http://www.broadinstitute.org/gsea/msigdb/cards/REGULATION_OF_BODY_FLUID_LEVELS |
| c5.mf.v5.0.entrez.gmt.msig.set | 0.0001 | 0.2 | 0.0537892 | 114 | 6 | 0.00069993 | ENZYME_INHIBITOR_ACTIVITY http://www.broadinstitute.org/gsea/msigdb/cards/ENZYME_INHIBITOR_ACTIVITY |
| c5.mf.v5.0.entrez.gmt.msig.set | 0.0001 | 0.3 | 0.039792 | 114 | 6 | 0.00049995 | ENZYME_INHIBITOR_ACTIVITY http://www.broadinstitute.org/gsea/msigdb/cards/ENZYME_INHIBITOR_ACTIVITY |
| ***TRD vs. healthy controls*** | | | | | | | |
| kegg.set | 0.0005 | 0.2 | 0.0353929 | 31 | 6 | 0.00039996 | 00250 Alanine,_aspartate_and_glutamate_metabolism |
| kegg.set | 0.0005 | 0.3 | 0.0361928 | 31 | 6 | 0.00039996 | 00250 Alanine,_aspartate_and_glutamate_metabolism |
| kegg.set | 0.0005 | 0.5 | 0.0431914 | 31 | 6 | 0.00069993 | 00250 Alanine,_aspartate_and_glutamate_metabolism |
| c2.cp.kegg.v5.0.entrez.gmt.msig.set | 0.0005 | 0.2 | 0.034993 | 31 | 6 | 0.00049995 | KEGG_ALANINE_ASPARTATE_AND_GLUTAMATE_METABOLISM http://www.broadinstitute.org/gsea/msigdb/cards/KEGG_ALANINE_ASPARTATE_AND_GLUTAMATE_METABOLISM |
| c2.cp.kegg.v5.0.entrez.gmt.msig.set | 0.0005 | 0.3 | 0.0225955 | 31 | 6 | 0.00019998 | KEGG_ALANINE_ASPARTATE_AND_GLUTAMATE_METABOLISM http://www.broadinstitute.org/gsea/msigdb/cards/KEGG_ALANINE_ASPARTATE_AND_GLUTAMATE_METABOLISM |
| c2.cp.kegg.v5.0.entrez.gmt.msig.set | 0.0005 | 0.5 | 0.0407918 | 31 | 6 | 0.00069993 | KEGG_ALANINE_ASPARTATE_AND_GLUTAMATE_METABOLISM http://www.broadinstitute.org/gsea/msigdb/cards/KEGG_ALANINE_ASPARTATE_AND_GLUTAMATE_METABOLISM |
| c2.cp.reactome.v5.0.entrez.gmt.msig.set | 0.0005 | 0.2 | 0.0283943* | 13 | 5 | 0.00009999 | REACTOME_ACTIVATION_OF_RAC http://www.broadinstitute.org/gsea/msigdb/cards/REACTOME_ACTIVATION_OF_RAC |
| c2.cp.reactome.v5.0.entrez.gmt.msig.set | 0.0005 | 0.2 | 0.0613877 | 29 | 8 | 0.00039996 | REACTOME_SIGNALING_BY_ROBO_RECEPTOR http://www.broadinstitute.org/gsea/msigdb/cards/REACTOME_SIGNALING_BY_ROBO_RECEPTOR |
| c2.cp.reactome.v5.0.entrez.gmt.msig.set | 0.0005 | 0.3 | 0.0423915 | 13 | 5 | 0.00019998 | REACTOME_ACTIVATION_OF_RAC http://www.broadinstitute.org/gsea/msigdb/cards/REACTOME_ACTIVATION_OF_RAC |
| c2.cp.reactome.v5.0.entrez.gmt.msig.set | 0.0005 | 0.3 | 0.030194* | 29 | 8 | 0.00009999 | REACTOME_SIGNALING_BY_ROBO_RECEPTOR http://www.broadinstitute.org/gsea/msigdb/cards/REACTOME_SIGNALING_BY_ROBO_RECEPTOR |
| c2.cp.reactome.v5.0.entrez.gmt.msig.set | 0.0005 | 0.5 | 0.0409918 | 13 | 5 | 0.00019998 | REACTOME_ACTIVATION_OF_RAC http://www.broadinstitute.org/gsea/msigdb/cards/REACTOME_ACTIVATION_OF_RAC |
| c2.cp.reactome.v5.0.entrez.gmt.msig.set | 0.0005 | 0.5 | 0.065187 | 29 | 8 | 0.00039996 | REACTOME_SIGNALING_BY_ROBO_RECEPTOR http://www.broadinstitute.org/gsea/msigdb/cards/REACTOME_SIGNALING_BY_ROBO_RECEPTOR |
| c2.cp.v5.0.entrez.gmt.msig.set | 0.0005 | 0.2 | 0.0833833 | 31 | 6 | 0.00019998 | KEGG_ALANINE_ASPARTATE_AND_GLUTAMATE_METABOLISM http://www.broadinstitute.org/gsea/msigdb/cards/KEGG_ALANINE_ASPARTATE_AND_GLUTAMATE_METABOLISM |
| c2.cp.v5.0.entrez.gmt.msig.set | 0.0005 | 0.3 | 0.0795841 | 31 | 6 | 0.00019998 | KEGG_ALANINE_ASPARTATE_AND_GLUTAMATE_METABOLISM http://www.broadinstitute.org/gsea/msigdb/cards/KEGG_ALANINE_ASPARTATE_AND_GLUTAMATE_METABOLISM |
| c2.cp.v5.0.entrez.gmt.msig.set | 0.0005 | 0.3 | 0.0545891* | 13 | 5 | 0.00009999 | REACTOME_ACTIVATION_OF_RAC http://www.broadinstitute.org/gsea/msigdb/cards/REACTOME_ACTIVATION_OF_RAC |
| c2.cp.v5.0.entrez.gmt.msig.set | 0.0005 | 0.5 | 0.0971806 | 19 | 5 | 0.00029997 | PID_ARF_3PATHWAY http://www.broadinstitute.org/gsea/msigdb/cards/PID_ARF_3PATHWAY |
| c2.cp.v5.0.entrez.gmt.msig.set | 0.0005 | 0.5 | 0.0971806 | 33 | 6 | 0.00029997 | PID_INTEGRIN_A4B1_PATHWAY http://www.broadinstitute.org/gsea/msigdb/cards/PID_INTEGRIN_A4B1_PATHWAY |
| c2.cp.v5.0.entrez.gmt.msig.set | 0.0005 | 0.5 | 0.079984 | 13 | 5 | 0.00019998 | REACTOME_ACTIVATION_OF_RAC http://www.broadinstitute.org/gsea/msigdb/cards/REACTOME_ACTIVATION_OF_RAC |
| c1.all.v5.0.entrez.gmt.msig.set | 0.0001 | 0.2 | 0.0255949 | 45 | 3 | 0.00029997 | chr18q12 http://www.broadinstitute.org/gsea/msigdb/cards/chr18q12 |
| c1.all.v5.0.entrez.gmt.msig.set | 0.0001 | 0.3 | 0.0995801 | 89 | 4 | 0.00209979 | chr10q23 http://www.broadinstitute.org/gsea/msigdb/cards/chr10q23 |
| c1.all.v5.0.entrez.gmt.msig.set | 0.0001 | 0.3 | 0.0153969 | 45 | 3 | 0.00019998 | chr18q12 http://www.broadinstitute.org/gsea/msigdb/cards/chr18q12 |
| c1.all.v5.0.entrez.gmt.msig.set | 0.0001 | 0.5 | 0.0125975 | 45 | 3 | 0.00019998 | chr18q12 http://www.broadinstitute.org/gsea/msigdb/cards/chr18q12 |
| c1.all.v5.0.entrez.gmt.msig.set | 0.0005 | 0.3 | 0.0987802 | 73 | 7 | 0.00119988 | chr13q12 http://www.broadinstitute.org/gsea/msigdb/cards/chr13q12 |
| c3.all.v5.0.entrez.gmt.msig.set | 0.0005 | 0.5 | 0.0915817 | 5 | 4 | 0.00049995 | GTCGATC,MIR-369-5P http://www.broadinstitute.org/gsea/msigdb/cards/GTCGATC,MIR-369-5P |
| c3.mir.v5.0.entrez.gmt.msig.set | 0.0005 | 0.2 | 0.025195 | 5 | 4 | 0.00019998 | GTCGATC,MIR-369-5P http://www.broadinstitute.org/gsea/msigdb/cards/GTCGATC,MIR-369-5P |
| c3.mir.v5.0.entrez.gmt.msig.set | 0.0005 | 0.5 | 0.0621876 | 5 | 4 | 0.00069993 | GTCGATC,MIR-369-5P http://www.broadinstitute.org/gsea/msigdb/cards/GTCGATC,MIR-369-5P |
| c3.tft.v5.0.entrez.gmt.msig.set | 0.00005 | 0.3 | 0.0957808 | 72 | 4 | 0.00179982 | V$GNCF_01 http://www.broadinstitute.org/gsea/msigdb/cards/V$GNCF_01 |
| c3.tft.v5.0.entrez.gmt.msig.set | 0.00005 | 0.5 | 0.0973805 | 72 | 4 | 0.00159984 | V$GNCF_01 http://www.broadinstitute.org/gsea/msigdb/cards/V$GNCF_01 |
| c4.cgn.v5.0.entrez.gmt.msig.set | 0.0005 | 0.3 | 0.0875825 | 35 | 6 | 0.00089991 | GNF2_PTX3 http://www.broadinstitute.org/gsea/msigdb/cards/GNF2_PTX3 |
| c4.cgn.v5.0.entrez.gmt.msig.set | 0.0005 | 0.3 | 0.0915817 | 75 | 7 | 0.0009999 | MORF_ATOX1 http://www.broadinstitute.org/gsea/msigdb/cards/MORF_ATOX1 |
| c4.cgn.v5.0.entrez.gmt.msig.set | 0.0005 | 0.5 | 0.0781844 | 75 | 7 | 0.0009999 | MORF_ATOX1 http://www.broadinstitute.org/gsea/msigdb/cards/MORF_ATOX1 |
| c5.all.v5.0.entrez.gmt.msig.set | 0.0005 | 0.2 | 0.079984 | 11 | 3 | 0.00019998 | ENDONUCLEASE_ACTIVITY_GO_0016893 http://www.broadinstitute.org/gsea/msigdb/cards/ENDONUCLEASE_ACTIVITY_GO_0016893 |
| c5.all.v5.0.entrez.gmt.msig.set | 0.0005 | 0.2 | 0.079984 | 13 | 3 | 0.00019998 | ENDORIBONUCLEASE_ACTIVITY http://www.broadinstitute.org/gsea/msigdb/cards/ENDORIBONUCLEASE_ACTIVITY |
| c5.bp.v5.0.entrez.gmt.msig.set | 0.0005 | 0.2 | 0.0881824 | 13 | 5 | 0.00049995 | PROTEIN_TETRAMERIZATION http://www.broadinstitute.org/gsea/msigdb/cards/PROTEIN_TETRAMERIZATION |
| c5.bp.v5.0.entrez.gmt.msig.set | 0.0005 | 0.5 | 0.0783843 | 13 | 5 | 0.00039996 | PROTEIN_TETRAMERIZATION http://www.broadinstitute.org/gsea/msigdb/cards/PROTEIN_TETRAMERIZATION |
| c7.all.v5.0.entrez.gmt.msig.set | 0.00001 | 0.2 | 0.0625875* | 185 | 4 | 0.00009999 | GSE29618_BCELL_VS_PDC_DAY7_FLU_VACCINE_UP http://www.broadinstitute.org/gsea/msigdb/cards/GSE29618_BCELL_VS_PDC_DAY7_FLU_VACCINE_UP |
| c7.all.v5.0.entrez.gmt.msig.set | 0.00001 | 0.3 | 0.069786 | 185 | 4 | 0.00019998 | GSE29618_BCELL_VS_PDC_DAY7_FLU_VACCINE_UP http://www.broadinstitute.org/gsea/msigdb/cards/GSE29618_BCELL_VS_PDC_DAY7_FLU_VACCINE_UP |
| c7.all.v5.0.entrez.gmt.msig.set | 0.00001 | 0.5 | 0.0575885* | 185 | 3 | 0.00009999 | GSE29618_BCELL_VS_PDC_DAY7_FLU_VACCINE_UP http://www.broadinstitute.org/gsea/msigdb/cards/GSE29618_BCELL_VS_PDC_DAY7_FLU_VACCINE_UP |
| h.all.v5.0.entrez.gmt.msig.set | 0.0005 | 0.5 | 0.019996 | 192 | 15 | 0.00089991 | HALLMARK_EPITHELIAL_MESENCHYMAL_TRANSITION http://www.broadinstitute.org/gsea/msigdb/cards/HALLMARK_EPITHELIAL_MESENCHYMAL_TRANSITION |
| h.all.v5.0.entrez.gmt.msig.set | 0.0005 | 0.5 | 0.0673865 | 111 | 8 | 0.00339966 | HALLMARK_UNFOLDED_PROTEIN_RESPONSE http://www.broadinstitute.org/gsea/msigdb/cards/HALLMARK_UNFOLDED_PROTEIN_RESPONSE |
| ***non-TRD vs. healthy controls*** | | | | | | | |
| kegg.set | 0.0005 | 0.2 | 0.0923815 | 54 | 8 | 0.00139986 | 05221 Acute_myeloid_leukemia |
| kegg.set | 0.0005 | 0.3 | 0.0675865 | 54 | 8 | 0.00109989 | 05221 Acute_myeloid_leukemia |
| kegg.set | 0.0005 | 0.5 | 0.0615877 | 54 | 8 | 0.00079992 | 05221 Acute_myeloid_leukemia |
| c2.cp.kegg.v5.0.entrez.gmt.msig.set | 0.0005 | 0.2 | 0.0673865 | 54 | 8 | 0.00109989 | KEGG_ACUTE_MYELOID_LEUKEMIA http://www.broadinstitute.org/gsea/msigdb/cards/KEGG_ACUTE_MYELOID_LEUKEMIA |
| c2.cp.kegg.v5.0.entrez.gmt.msig.set | 0.0005 | 0.3 | 0.089982 | 54 | 8 | 0.00169983 | KEGG_ACUTE_MYELOID_LEUKEMIA http://www.broadinstitute.org/gsea/msigdb/cards/KEGG_ACUTE_MYELOID_LEUKEMIA |
| c2.cp.kegg.v5.0.entrez.gmt.msig.set | 0.0005 | 0.5 | 0.0387922 | 54 | 8 | 0.00059994 | KEGG_ACUTE_MYELOID_LEUKEMIA http://www.broadinstitute.org/gsea/msigdb/cards/KEGG_ACUTE_MYELOID_LEUKEMIA |
| c2.cp.reactome.v5.0.entrez.gmt.msig.set | 0.0005 | 0.2 | 0.0291942* | 40 | 12 | 0.00009999 | REACTOME_NOTCH1_INTRACELLULAR_DOMAIN_REGULATES_TRANSCRIPTION http://www.broadinstitute.org/gsea/msigdb/cards/REACTOME_NOTCH1_INTRACELLULAR_DOMAIN_REGULATES_TRANSCRIPTION |
| c2.cp.reactome.v5.0.entrez.gmt.msig.set | 0.0005 | 0.2 | 0.0291942* | 64 | 13 | 0.00009999 | REACTOME_SIGNALING_BY_NOTCH1 http://www.broadinstitute.org/gsea/msigdb/cards/REACTOME_SIGNALING_BY_NOTCH1 |
| c2.cp.reactome.v5.0.entrez.gmt.msig.set | 0.0005 | 0.3 | 0.0841832 | 40 | 11 | 0.00049995 | REACTOME_NOTCH1_INTRACELLULAR_DOMAIN_REGULATES_TRANSCRIPTION http://www.broadinstitute.org/gsea/msigdb/cards/REACTOME_NOTCH1_INTRACELLULAR_DOMAIN_REGULATES_TRANSCRIPTION |
| c2.cp.reactome.v5.0.entrez.gmt.msig.set | 0.0005 | 0.3 | 0.0419916 | 64 | 12 | 0.00019998 | REACTOME_SIGNALING_BY_NOTCH1 http://www.broadinstitute.org/gsea/msigdb/cards/REACTOME_SIGNALING_BY_NOTCH1 |
| c2.cp.reactome.v5.0.entrez.gmt.msig.set | 0.0005 | 0.5 | 0.044791 | 40 | 11 | 0.00019998 | REACTOME_NOTCH1_INTRACELLULAR_DOMAIN_REGULATES_TRANSCRIPTION http://www.broadinstitute.org/gsea/msigdb/cards/REACTOME_NOTCH1_INTRACELLULAR_DOMAIN_REGULATES_TRANSCRIPTION |
| c2.cp.reactome.v5.0.entrez.gmt.msig.set | 0.0005 | 0.5 | 0.0633873 | 64 | 12 | 0.00029997 | REACTOME_SIGNALING_BY_NOTCH1 http://www.broadinstitute.org/gsea/msigdb/cards/REACTOME_SIGNALING_BY_NOTCH1 |
| c2.cp.v5.0.entrez.gmt.msig.set | 0.0005 | 0.2 | 0.0869826 | 27 | 6 | 0.00019998 | PID_TRAIL_PATHWAY http://www.broadinstitute.org/gsea/msigdb/cards/PID_TRAIL_PATHWAY |
| c2.cp.v5.0.entrez.gmt.msig.set | 0.0005 | 0.2 | 0.0869826 | 40 | 12 | 0.00019998 | REACTOME_NOTCH1_INTRACELLULAR_DOMAIN_REGULATES_TRANSCRIPTION http://www.broadinstitute.org/gsea/msigdb/cards/REACTOME_NOTCH1_INTRACELLULAR_DOMAIN_REGULATES_TRANSCRIPTION |
| c2.cp.v5.0.entrez.gmt.msig.set | 0.0005 | 0.2 | 0.0603879* | 64 | 13 | 0.00009999 | REACTOME_SIGNALING_BY_NOTCH1 http://www.broadinstitute.org/gsea/msigdb/cards/REACTOME_SIGNALING_BY_NOTCH1 |
| c2.cp.v5.0.entrez.gmt.msig.set | 0.0005 | 0.3 | 0.0675865* | 27 | 6 | 0.00009999 | PID_TRAIL_PATHWAY http://www.broadinstitute.org/gsea/msigdb/cards/PID_TRAIL_PATHWAY |
| c2.cp.v5.0.entrez.gmt.msig.set | 0.0005 | 0.5 | 0.0757848 | 27 | 6 | 0.00019998 | PID_TRAIL_PATHWAY http://www.broadinstitute.org/gsea/msigdb/cards/PID_TRAIL_PATHWAY |
| c2.cp.v5.0.entrez.gmt.msig.set | 0.0005 | 0.5 | 0.0757848 | 64 | 12 | 0.00019998 | REACTOME_SIGNALING_BY_NOTCH1 http://www.broadinstitute.org/gsea/msigdb/cards/REACTOME_SIGNALING_BY_NOTCH1 |
| c1.all.v5.0.entrez.gmt.msig.set | 0.0001 | 0.2 | 0.0141972 | 48 | 3 | 0.00019998 | chr10q25 http://www.broadinstitute.org/gsea/msigdb/cards/chr10q25 |
| c1.all.v5.0.entrez.gmt.msig.set | 0.0001 | 0.3 | 0.0133973* | 48 | 3 | 0.00009999 | chr10q25 http://www.broadinstitute.org/gsea/msigdb/cards/chr10q25 |
| c1.all.v5.0.entrez.gmt.msig.set | 0.0001 | 0.5 | 0.014997* | 48 | 3 | 0.00009999 | chr10q25 http://www.broadinstitute.org/gsea/msigdb/cards/chr10q25 |
| c1.all.v5.0.entrez.gmt.msig.set | 0.0001 | 0.5 | 0.0725855 | 62 | 3 | 0.00109989 | chr3q26 http://www.broadinstitute.org/gsea/msigdb/cards/chr3q26 |
| c1.all.v5.0.entrez.gmt.msig.set | 0.0005 | 0.2 | 0.019996* | 37 | 5 | 0.00009999 | chr5q12 http://www.broadinstitute.org/gsea/msigdb/cards/chr5q12 |
| c1.all.v5.0.entrez.gmt.msig.set | 0.0005 | 0.3 | 0.019796* | 37 | 5 | 0.00009999 | chr5q12 http://www.broadinstitute.org/gsea/msigdb/cards/chr5q12 |
| c1.all.v5.0.entrez.gmt.msig.set | 0.0005 | 0.5 | 0.0169966* | 37 | 5 | 0.00009999 | chr5q12 http://www.broadinstitute.org/gsea/msigdb/cards/chr5q12 |
| c3.mir.v5.0.entrez.gmt.msig.set | 0.00001 | 0.2 | 0.0831834 | 88 | 3 | 0.00279972 | AGGTGCA,MIR-500 http://www.broadinstitute.org/gsea/msigdb/cards/AGGTGCA,MIR-500 |
| c4.all.v5.0.entrez.gmt.msig.set | 0.00001 | 0.2 | 0.085183 | 71 | 4 | 0.00079992 | GNF2_XRCC5 http://www.broadinstitute.org/gsea/msigdb/cards/GNF2_XRCC5 |
| c4.cgn.v5.0.entrez.gmt.msig.set | 0.00001 | 0.2 | 0.0663867 | 71 | 4 | 0.00119988 | GNF2_XRCC5 http://www.broadinstitute.org/gsea/msigdb/cards/GNF2_XRCC5 |
| c4.cgn.v5.0.entrez.gmt.msig.set | 0.00001 | 0.3 | 0.0865827 | 71 | 4 | 0.00189981 | GNF2_XRCC5 http://www.broadinstitute.org/gsea/msigdb/cards/GNF2_XRCC5 |
| c4.cm.v5.0.entrez.gmt.msig.set | 0.00001 | 0.5 | 0.0869826 | 175 | 3 | 0.0039996 | MODULE_93 http://www.broadinstitute.org/gsea/msigdb/cards/MODULE_93 |
| c5.all.v5.0.entrez.gmt.msig.set | 0.0001 | 0.2 | 0.0579884* | 11 | 4 | 0.00009999 | NUCLEAR_MATRIX http://www.broadinstitute.org/gsea/msigdb/cards/NUCLEAR_MATRIX |
| c5.all.v5.0.entrez.gmt.msig.set | 0.0001 | 0.3 | 0.0883823 | 11 | 4 | 0.00019998 | NUCLEAR_MATRIX http://www.broadinstitute.org/gsea/msigdb/cards/NUCLEAR_MATRIX |
| c5.all.v5.0.entrez.gmt.msig.set | 0.0005 | 0.2 | 0.0581884* | 11 | 5 | 0.00009999 | NUCLEAR_MATRIX http://www.broadinstitute.org/gsea/msigdb/cards/NUCLEAR_MATRIX |
| c5.all.v5.0.entrez.gmt.msig.set | 0.0005 | 0.3 | 0.0655869* | 11 | 5 | 0.00009999 | NUCLEAR_MATRIX http://www.broadinstitute.org/gsea/msigdb/cards/NUCLEAR_MATRIX |
| c5.all.v5.0.entrez.gmt.msig.set | 0.0005 | 0.5 | 0.0591882* | 11 | 5 | 0.00009999 | NUCLEAR_MATRIX http://www.broadinstitute.org/gsea/msigdb/cards/NUCLEAR_MATRIX |
| c5.bp.v5.0.entrez.gmt.msig.set | 0.0005 | 0.3 | 0.0871826 | 17 | 4 | 0.00039996 | NEGATIVE_REGULATION_OF_DNA_METABOLIC_PROCESS http://www.broadinstitute.org/gsea/msigdb/cards/NEGATIVE_REGULATION_OF_DNA_METABOLIC_PROCESS |
| c5.cc.v5.0.entrez.gmt.msig.set | 0.0001 | 0.3 | 0.079784 | 11 | 3 | 0.00179982 | NUCLEAR_MATRIX http://www.broadinstitute.org/gsea/msigdb/cards/NUCLEAR_MATRIX |
| c5.cc.v5.0.entrez.gmt.msig.set | 0.0001 | 0.5 | 0.0863827 | 11 | 3 | 0.0019998 | NUCLEAR_MATRIX http://www.broadinstitute.org/gsea/msigdb/cards/NUCLEAR_MATRIX |

Table S4 Exon-specific eQTL findings from BRAINEAC

| **geneSymbol** | **marker** | **rsid** | **exprID** | **chr** | **start** | **stop** | **aveALL** | **CRBL** | **FCTX** | **HIPP** | **MEDU** | **OCTX** | **PUTM** | **SNIG** | **TCTX** | **THAL** | **WHMT** |
| --- | --- | --- | --- | --- | --- | --- | --- | --- | --- | --- | --- | --- | --- | --- | --- | --- | --- |
| HERC5 | chr4:90421353 | rs1908557 | 2735440 | chr4 | 89378307 | 89430798 | 4.1e-02 | 7.2e-01 | 1.9e-01 | 5.4e-01 | 8.9e-01 | 2.4e-02 | 6.1e-05 | 8.7e-01 | 8.6e-01 | 5.8e-01 | 2.0e-01 |
| FAM13A | chr4:90421353 | rs1908557 | 2777561 | chr4 | 89772171 | 89772314 | 1.5e-04 | 7.6e-03 | 5.1e-01 | 4.5e-02 | 1.2e-01 | 4.2e-01 | 8.9e-01 | 6.1e-01 | 3.8e-01 | 1.1e-02 | 1.7e-01 |
| FAM13A | chr4:90421353 | rs1908557 | t2777560 | chr4 | 89772171 | 89772314 | 1.5e-04 | 7.6e-03 | 5.1e-01 | 4.5e-02 | 1.2e-01 | 4.2e-01 | 8.9e-01 | 6.1e-01 | 3.8e-01 | 1.1e-02 | 1.7e-01 |
| FAM190A | chr4:90421353 | rs1908557 | 2735887 | chr4 | 91048684 | 92523040 | 7.2e-03 | 1.5e-03 | 5.6e-01 | 2.4e-01 | 9.2e-01 | 3.1e-03 | 3.8e-02 | 8.2e-01 | 7.3e-01 | 1.0e-01 | 8.2e-01 |
| MMRN1 | chr4:90421353 | rs1908557 | 2735777 | chr4 | 90800671 | 90875759 | 3.7e-01 | 4.4e-01 | 6.2e-01 | 2.4e-01 | 2.4e-01 | 1.5e-03 | 9.2e-01 | 9.6e-01 | 9.5e-01 | 2.5e-01 | 8.4e-01 |
| HERC5 | chr4:90421353 | rs1908557 | 2735442 | chr4 | 89378307 | 89430798 | 3.6e-01 | 1.1e-01 | 5.9e-01 | 1.4e-01 | 2.4e-01 | 6.5e-01 | 2.6e-01 | 7.8e-01 | 8.2e-01 | 4.9e-02 | 2.5e-03 |
| FAM13A | chr4:90421353 | rs1908557 | 2777514 | chr4 | 89645913 | 89744421 | 2.6e-03 | 5.7e-01 | 9.3e-01 | 3.2e-01 | 4.8e-01 | 2.6e-02 | 9.3e-01 | 1.8e-01 | 8.8e-01 | 8.1e-02 | 2.6e-02 |
| FAM13A | chr4:90421353 | rs1908557 | 2777577 | chr4 | 89776162 | 89978303 | 7.8e-01 | 6.5e-01 | 1.2e-01 | 7.6e-01 | 7.6e-01 | 1.2e-01 | 9.5e-01 | 3.0e-03 | 9.2e-01 | 1.0e+00 | 5.0e-02 |
| FAM13A | chr4:90421353 | rs1908557 | 2777536 | chr4 | 89645913 | 89744421 | 3.0e-03 | 3.6e-01 | 7.8e-01 | 2.5e-01 | 1.7e-01 | 4.2e-02 | 9.5e-01 | 4.4e-01 | 8.7e-01 | 7.2e-02 | 8.9e-03 |
| FAM13A | chr4:90421353 | rs1908557 | 2777543 | chr4 | 89645913 | 89744421 | 3.5e-03 | 2.7e-02 | 3.6e-01 | 2.2e-02 | 4.1e-01 | 7.7e-02 | 2.3e-01 | 1.5e-01 | 4.1e-01 | 4.9e-01 | 8.8e-03 |

Source: <http://braineac.org/> accessed on March 12, 2016**.** This exon-specific eQTL data set covering ten human brain regions where exon-specific RNA expression was quantified using Affymetrix Human Exon 1.0 ST arrays in 10 brain regions: cerebellar cortex (CRBL), frontal cortex (FCTX), hippocampus (HIPP), inferior olivary nucleus (sub-dissected from the medulla, MEDU), occipital cortex (OCTX), putamen (at the level of the anterior commissure, PUTM), substantia nigra (SNIG), temporal cortex (TCTX), thalamus (at the level of the lateral geniculate nucleus, THAL) and intralobular white matter (WHMT).

**Text S1** Genome Wide Association AnalysisWe restrict participants to a set of individuals who have >97% European ancestry, as determined through an analysis of local ancestry^1^. Briefly, the algorithm first partitions phased genomic data into short windows of about 100 SNPs. Within each window, we use a support vector machine (SVM) to classify individual haplotypes into one of 31 reference populations. The SVM classifications are then fed into a hidden Markov model (HMM) that accounts for switch errors and incorrect assignments, and gives probabilities for each reference population in each window. Finally, we used simulated admixed individuals to recalibrate the HMM probabilities so that the reported assignments are consistent with the simulated admixture proportions. The reference population data is derived from public datasets (the Human Genome Diversity Project, HapMap, and 1000 Genomes), as well as 23andMe customers who have reported having four grandparents from the same country.

A maximal set of unrelated individuals was chosen for each analysis using a segmental identity-by-descent (IBD) estimation algorithm.[^1^](#_ENREF_1) Individuals were defined as related if they shared more than 700 cM IBD, including regions where the two individuals share either one or both genomic segments identical-by-descent. This level of relatedness (roughly 20% of the genome) corresponds approximately to the minimal expected sharing between first cousins in an outbred population.

Participant genotype data were imputed against the September 2013 release of 1000 Genomes[^2^](#_ENREF_2) Phase1 reference haplotypes, phased with ShapeIt2. We phased and imputed data for each genotyping platform separately. We phased using a phasing tool Finch developed by 23andMe, Inc. which implements the Beagle[^3^](#_ENREF_3) haplotype graph-based phasing algorithm, modified to separate the haplotype graph construction and phasing steps. Finch extends the Beagle model to accommodate genotyping error and recombination, to handle cases where there are no consistent paths through the haplotype graph for the individual being phased. We constructed haplotype graphs for European and non-European samples on each 23andMe genotyping platform from a representative sample of genotyped individuals, and then performed out-of-sample phasing of all genotyped individuals against the appropriate graph.

In preparation for imputation, we split phased chromosomes into segments of no more than 10,000 genotyped SNPs, with overlaps of 200 SNPs. We excluded SNPs with Hardy-Weinberg equilibrium *P*<10^−20^, call rate < 95%, or with large allele frequency discrepancies compared to European 1000 Genomes reference data. Frequency discrepancies were identified by computing a 2x2 table of allele counts for European 1000 Genomes samples and 2000 randomly sampled 23andMe members with European ancestry, and identifying SNPs with a chi squared P<10^−15^. We imputed each phased segment against all-ethnicity 1000 Genomes haplotypes (excluding monomorphic and singleton sites) using Minimac2[^4^](#_ENREF_4), using 5 rounds and 200 states for parameter estimation.

# Association Test Results

For quality control of genotyped GWAS results, we excluded SNPs that were only genotyped on our “V1” and/or “V2” platforms due to small sample size, and SNPs on chrM or chrY because many of these are not currently called reliably. Using trio data, we excluded SNPs that failed a test for parent-offspring transmission; specifically, we regressed the child’s allele count against the mean parental allele count and excluded SNPs with fitted β < 0.6 and P < 10^−20^ for a test of β<1. We excluded SNPs with a Hardy-Weinberg P < 10^−20^ in Europeans; or a call rate of < 90%. We also tested genotyped SNPs for genotype date effects, and excluded SNPs with P < 10^−50^ by ANOVA of SNP genotypes against a factor dividing genotyping date into 20 roughly equal-sized buckets.

For imputed GWAS results, we excluded SNPs with avg.rsq < 0.5 or min.rsq < 0.3 in any imputation batch, as well as SNPs that had strong evidence of an imputation batch effect. The batch effect test is an F test from an ANOVA of the SNP dosages against a factor representing imputation batch; we excluded results with P<10^−50^.

When choosing between imputed and genotyped GWAS results, if either the imputed test passes quality control, or a genotyped test is unavailable, we report the imputed result; otherwise, we report the genotyped result.

Across all results, we excluded logistic regression results that did not converge due to complete separation, identified by abs(effect) > 10 or stderr > 10 on the log odds scale.

**Text S2** Possible causative genes near top bupropion hit rs1908557

We have identified a candidate genetic marker rs1908557 in the intergenic region between *GPRIN3* and *SNCA* for bupropion response with an association p-value passing genome wide significance. It is not clear if the intergenic variant has any biological impact on the neighboring gene *SNCA*, which is expressed in multiple brain regions (Figure S3). Intriguingly, SNCA encodes alpha-synuclein, which is a member of the synuclein family that also includes beta- and gamma-synuclein. Synucleins are abundantly expressed in the brain and alpha- and beta-synuclein inhibit phospholipase D2 selectively. *SNCA* may serve to integrate presynaptic signaling and membrane trafficking. Defects in *SNCA* have been implicated in the pathogenesis of Parkinson disease. *SNCA* peptides are a major component of amyloid plaques in the brains of patients with Alzheimer's disease. Hippocampal differential protein profiling also indicated stress susceptibility to be associated with deficits in synaptic vesicle release involving SNCA, SYN-1, and AP-3 in a chronic mild stress rat model of depression.[^5^](#_ENREF_5) eQTL data from BRAINEAC[**^6^**](#_ENREF_6) (<http://braineac.org/>) however suggested a subtle cis-eQTL relationship between rs1908557 and genes further away from rs1908557 such as family with sequence similarity 13 member A (*FAM13A*), multimerin 1 (*MMRN1*), HECT and RLD domain containing E3 ubiquitin protein ligase 5 (*HERC5*), and coiled-coil serine rich protein 1 (*CCSER1*, also known as *FAM190A*) (See Table S4 in Supplement 1). Both *FAM13A* and *CCSER1* are expressed in the cerebellum (Figure S3)**,** which regulates motor movements**.** Polymorphisms from *CCSER1* have been implicated in attention deficit/hyperactivity disorder (ADHD).[^7^](#_ENREF_7)   Brain imaging studies have shown reduction of the posterior inferior cerebellar lobules volume of ADHD boys and girls compared to controls, persistent with age and not present in unaffected siblings[**^8^**](#_ENREF_8)**^,^** [**^9^**](#_ENREF_9), suggesting that the cerebellum may be directly related to pathophysiology of ADHD. FAM13A on the other hand has been implicated in chronic obstructive pulmonary disease (COPD)[^10^](#_ENREF_10). The association finding between rs1908557 and bupropion response needs to be replicated in an independent population.

**Text S3** Discussion of variants reported in the prior candidate gene or GWAS studies

Among the candidate gene studies, brain-derived neurotrophic factor (*BDNF*), which has been implicated in neuronal plasticity and apoptosis, is one of the most studied candidate genes. BDNF exists in two forms. The precursor pro-BDNF is cleaved to produce mature BDNF. BDNF binds to its high affinity receptor tyro­sine kinase receptor B (*TRKB*) to enhance neuronal plasticity, while the binding of pro-BDNF to its other receptor p75 neu­rotrophin receptor (*P75NTR*) (alternatively named as *NGFR* or tumor necrosis factor superfamily member 16, *TNFRSF16*) results in programmed neuronal death, neurite retraction and synaptic pruning[^11^](#_ENREF_11). The relationship between polymorphisms from *BDNF*, *TRKB,* and *P75NTR* and antidepressant efficacy was extensively reviewed by Colle et al.,[^12^](#_ENREF_12) with the strongest evidence from Met allele (decrease dendritic trafficking activity) from rs6265 (Val66Met) being associated with better antidepressant efficacy in Asian population from multiple studies. In this current study Met allele of rs6265 was trended towards better response with a nominal association (p = 0.07) in patients receiving citalopram / escitalopram (OR = 1.1).

Polymorphism from pharmacokinetics (PK) and pharmacodynamics (PD) genes may likewise influence antidepressant efficacy. In the International Study to Predict Optimized Treatment in Depression (iSPOT-D), common homozygote variant rs10245483 (G/G) from *ABCB1* (*MDR1*) locus, which encodes P-glycoprotein, have been reported to be associated with better response to escitalopram and sertraline.[^13^](#_ENREF_13) In this study, the rare T allele of rs10245483 was trended towards worse response to citalopram / escitalopram and SSRI, (nominal p = 0.1 and 0.05, OR = 0.93 and 0.94, respectively), consistent with the finding from iSPOT-D. An intronic variant rs7997012 from Serotonin 2A Receptor (*HTR2A*) was reported by McMahon et al., to be associated with citalopram response in STAR*D samples.[^14^](#_ENREF_14) Intriguingly, the same variant was marginally associated with citalopram / escitalopram response vs. non-response (p = 0.05), although A allele was associated with worse citalopram / escitalopram response, opposite to the direction observed in STAR*D. Similar to the McMahon et al., there was no association for other *HTR2A* variants (rs1928040, rs6313, and rs6311) from the citalopram / escitalopram response vs. non-response analysis. Variant rs7997012 also exhibited a trend towards marginally association in SSRI response vs. non-response (p = 0.08), SSRI responders vs. healthy controls (p = 0.02), and citalopram / escitalopram responders vs. healthy controls (p = 0.09) analyses. The functional variants rs6313 and rs6311[^15^](#_ENREF_15)^,^ [^16^](#_ENREF_16) was demonstrated to impact promoter activity on the other hand exhibited marginal association in bupropion response vs. non-response (p = 0.05), bupropion responders vs. healthy controls (p = 0.04 and 0.05, respectively). Both SNPs were in strong LD with each other and to a less extent with rs1928040, but they were not in significant LD with rs7997012. Variant rs165599 from Catechol-O-Methyltransferase (*COMT*) was reported to be associated with duloxetine response in a cohort of 250 MDD patients of European decent.[^17^](#_ENREF_17) Specially, subjects with GG genotype had the greatest reduction in HAM-D_17_ score and subjects with AA genotypes had the least response. There was no association (p > 0.05) for this variant in any of our analyses. Likewise, several suggestive association signals from previous GWAS studies[^18-22^](#_ENREF_18) were previously reported. In the MARS, GENDEP, and STAR*D meta-analysis, rs2546057, rs12410462, rs17634917, and rs264272 were associated with percentage improvement (P <= 3.87 x10^-6^) and rs9601248, rs2125000, rs17710780, and rs9466930 were associated with remission (p <= 4.45 x 10^-6^).The T allele of rs9601248 was nominally associated with non-TRD (vs. TRD, OR = 1.12, p = 0.009) and bupropion response (vs. bupropion non-response OR = 1.10, p = 0.02) in this study. Additional variants identified from PGRN-AMPS GWAS study included rs11144870 SNP in riboflavin kinase (*RFK*) gene associated with eight week treatment response (OR = 0.42, p = 1.04×10^−6^) and rs915120 SNP in the G protein-coupled receptor kinase 5 (*GRK5*) gene associated with eight week remission (OR = 0.50, p = 1.15×10^−5^).[^21^](#_ENREF_21) Both variants were shown to be functional by influencing transcriptional activity in a reporter gene assay and altering nuclear protein binding in an electrophoretic mobility shift assay. In this study, the T allele of rs11144870 was associated with less chance of being TRD phenotype (vs. non-TRD or vs. healthy controls, p = 0.002 or 0.006, OR = 0.86 or 0.88, respectively, opposite to the direction for response / remission reported by the PGRN-AMPS study) but not with any of the other phenotypes. No association was observed for *GRK5* variant rs915120 or the 5-hydroxytryptamine serotonin receptor 1B (*HTR1B*) variants (p < 2 × 10^−5^ for rs1379887, rs7738598 and rs898040) highlighted in the PGRN-AMPS study.

Rs333079 from chromosome 1 near prokineticin 1 (*PROK1*) was reported to be suggestively associated with remission after up to 12 weeks of treatment in the entire GENDEP, MARS, and STAR*D samples (p = 3.07 x 10^-5^ in Supplementary Table S1b in the MARS, GENDEP, and STAR*D meta-analysis [^19^](#_ENREF_19)) and rs2232069 from chromosome 20 near prokineticin receptor 2 (*PROKR2*) was reported to be suggestively associated with early improvement (i.e. percentage improvement over 2 weeks of treatment) among SSRI-treated subjects in GENDEP and STAR*D (p_fixed_ = 0.0000176, p_random_ = 0.01996 in Supplementary Table S4a in the MARS, GENDEP, and STAR*D meta-analysis[^19^](#_ENREF_19)). Although both variants are not right on the *PROK1* and *PROKR2* genes, another variant rs4839421 also near *PROK1* (21,984 base pair away from *PROK1*, but not in LD with rs333079 (R-sq = 1.6196e-07, D' = 0.000457209)) was also suggestively associated with bupropion response (p = 4.07 x 10^-7^) in this study. Prok1 and Prok2 are cytokines exhibiting rhythmic expression in response to light in the zebrafish brain.[^23^](#_ENREF_23) The level of both Prok’s significantly increased at 5:00 (1h before the light was on).The level of Prok1 was maintained at a high expression level during the light period and gradually decreased before the light was off and reached its lowest level during the dark period. In contrast, the Prok2 transcript reached its highest level at dawn and then decreased to a basal level at 4 h after the light was on (10:00). Prok2 is expressed in the SCN and found to be circadian and light regulated.[^24^](#_ENREF_24)^,^ [^25^](#_ENREF_25) Light is the strongest factor that synchronizes the rhythms when the retina transmits the light impulses to the cells within SCN of the brain and the SCN later passes the signal through the peripheral tissue using Prok2.[^26^](#_ENREF_26)^,^ [^27^](#_ENREF_27) It is interesting that *PROK1* was not included in the kegg.set gene collection 04710 Circadian rhythm - mammal and it could have strengthened the enrichment of circadian rhythms gene set if included.

**Text S4** Additional discussions

Although the genetic correlation between bupropion response and non-TRD vs. TRD phenotype could not be estimated using LD score regression method, due to the negative h^2^ estimate for bupropion response phenotype. The variants showing suggestive association with bupropion response typically show a suggestive trend of association in TRD analysis as well. For example, the genome wide significant variant rs1908557 also showed a suggestive association in the TRD analysis (p = 0.002). Likewise, the circadian rhythm variant rs12137927 in *PER3*, rs2328741 in *GRM1* (p = 1.12 x 10^-5^), rs34337960 in *PRKCA* (p = 1.49 x 10^-5^) in bupropion response analysis showed suggestive associations in TRD (p = 0.03, 0.1, 0.01, respectively). The four responders vs. non-responders all had large standard error in h^2^ and genetic correlation estimates, perhaps due to the smaller sample size compared to (non-)responders vs. healthy controls analyses. The only significant genetic correlation is between SSRI responders vs. non-responders phenotype and non-TRD vs, TRD phenotype (p < 0.05).

**References**

1. Henn BM, Hon L, Macpherson JM, Eriksson N, Saxonov S, Pe'er I*, et al*. Cryptic distant relatives are common in both isolated and cosmopolitan genetic samples. *PloS one* 2012; **7**(4)**:** e34267.

2. Genomes Project C, Abecasis GR, Altshuler D, Auton A, Brooks LD, Durbin RM*, et al*. A map of human genome variation from population-scale sequencing. *Nature* 2010; **467**(7319)**:** 1061-1073.

3. Browning SR, Browning BL. Rapid and accurate haplotype phasing and missing-data inference for whole-genome association studies by use of localized haplotype clustering. *American journal of human genetics* 2007; **81**(5)**:** 1084-1097.

4. Fuchsberger C, Abecasis GR, Hinds DA. minimac2: faster genotype imputation. *Bioinformatics* 2015; **31**(5)**:** 782-784.

5. Henningsen K, Palmfeldt J, Christiansen S, Baiges I, Bak S, Jensen ON*, et al*. Candidate hippocampal biomarkers of susceptibility and resilience to stress in a rat model of depression. *Molecular & cellular proteomics : MCP* 2012; **11**(7)**:** M111 016428.

6. Ramasamy A, Trabzuni D, Guelfi S, Varghese V, Smith C, Walker R*, et al*. Genetic variability in the regulation of gene expression in ten regions of the human brain. *Nature neuroscience* 2014; **17**(10)**:** 1418-1428.

7. Lantieri F, Glessner JT, Hakonarson H, Elia J, Devoto M. Analysis of GWAS top hits in ADHD suggests association to two polymorphisms located in genes expressed in the cerebellum. *American journal of medical genetics Part B, Neuropsychiatric genetics : the official publication of the International Society of Psychiatric Genetics* 2010; **153B**(6)**:** 1127-1133.

8. Durston S, Hulshoff Pol HE, Schnack HG, Buitelaar JK, Steenhuis MP, Minderaa RB*, et al*. Magnetic resonance imaging of boys with attention-deficit/hyperactivity disorder and their unaffected siblings. *Journal of the American Academy of Child and Adolescent Psychiatry* 2004; **43**(3)**:** 332-340.

9. Castellanos FX, Lee PP, Sharp W, Jeffries NO, Greenstein DK, Clasen LS*, et al*. Developmental trajectories of brain volume abnormalities in children and adolescents with attention-deficit/hyperactivity disorder. *Jama* 2002; **288**(14)**:** 1740-1748.

10. Choo JY, Lee KY, Shin C, Kim S, Lee SK, Kang EY*, et al*. Quantitative analysis of lungs and airways with CT in subjects with the chronic obstructive pulmonary disease (COPD) candidate FAM13A gene: case control study for CT quantification in COPD risk gene. *Journal of computer assisted tomography* 2014; **38**(4)**:** 597-603.

11. Castren E, Rantamaki T. The role of BDNF and its receptors in depression and antidepressant drug action: Reactivation of developmental plasticity. *Developmental neurobiology* 2010; **70**(5)**:** 289-297.

12. Colle R, Deflesselle E, Martin S, David DJ, Hardy P, Taranu A*, et al*. BDNF/TRKB/P75NTR polymorphisms and their consequences on antidepressant efficacy in depressed patients. *Pharmacogenomics* 2015; **16**(9)**:** 997-1013.

13. Schatzberg AF, DeBattista C, Lazzeroni LC, Etkin A, Murphy GM, Jr., Williams LM. ABCB1 Genetic Effects on Antidepressant Outcomes: A Report From the iSPOT-D Trial. *The American journal of psychiatry* 2015; **172**(8)**:** 751-759.

14. McMahon FJ, Buervenich S, Charney D, Lipsky R, Rush AJ, Wilson AF*, et al*. Variation in the gene encoding the serotonin 2A receptor is associated with outcome of antidepressant treatment. *American journal of human genetics* 2006; **78**(5)**:** 804-814.

15. Parsons MJ, D'Souza UM, Arranz MJ, Kerwin RW, Makoff AJ. The -1438A/G polymorphism in the 5-hydroxytryptamine type 2A receptor gene affects promoter activity. *Biological psychiatry* 2004; **56**(6)**:** 406-410.

16. Polesskaya OO, Sokolov BP. Differential expression of the "C" and "T" alleles of the 5-HT2A receptor gene in the temporal cortex of normal individuals and schizophrenics. *Journal of neuroscience research* 2002; **67**(6)**:** 812-822.

17. Perlis RH, Fijal B, Adams DH, Sutton VK, Trivedi MH, Houston JP. Variation in catechol-O-methyltransferase is associated with duloxetine response in a clinical trial for major depressive disorder. *Biological psychiatry* 2009; **65**(9)**:** 785-791.

18. Garriock HA, Kraft JB, Shyn SI, Peters EJ, Yokoyama JS, Jenkins GD*, et al*. A genomewide association study of citalopram response in major depressive disorder. *Biological psychiatry* 2010; **67**(2)**:** 133-138.

19. Investigators G, Investigators M, Investigators SD. Common genetic variation and antidepressant efficacy in major depressive disorder: a meta-analysis of three genome-wide pharmacogenetic studies. *The American journal of psychiatry* 2013; **170**(2)**:** 207-217.

20. Ising M, Lucae S, Binder EB, Bettecken T, Uhr M, Ripke S*, et al*. A genomewide association study points to multiple loci that predict antidepressant drug treatment outcome in depression. *Archives of general psychiatry* 2009; **66**(9)**:** 966-975.

21. Ji Y, Biernacka JM, Hebbring S, Chai Y, Jenkins GD, Batzler A*, et al*. Pharmacogenomics of selective serotonin reuptake inhibitor treatment for major depressive disorder: genome-wide associations and functional genomics. *The pharmacogenomics journal* 2013; **13**(5)**:** 456-463.

22. Uher R, Perroud N, Ng MY, Hauser J, Henigsberg N, Maier W*, et al*. Genome-wide pharmacogenetics of antidepressant response in the GENDEP project. *The American journal of psychiatry* 2010; **167**(5)**:** 555-564.

23. Noonin C, Watthanasurorot A, Winberg S, Soderhall I. Circadian regulation of melanization and prokineticin homologues is conserved in the brain of freshwater crayfish and zebrafish. *Developmental and comparative immunology* 2013; **40**(2)**:** 218-226.

24. Cheng MY, Bullock CM, Li C, Lee AG, Bermak JC, Belluzzi J*, et al*. Prokineticin 2 transmits the behavioural circadian rhythm of the suprachiasmatic nucleus. *Nature* 2002; **417**(6887)**:** 405-410.

25. Cheng MY, Bittman EL, Hattar S, Zhou QY. Regulation of prokineticin 2 expression by light and the circadian clock. *BMC neuroscience* 2005; **6:** 17.

26. Wilking M, Ndiaye M, Mukhtar H, Ahmad N. Circadian rhythm connections to oxidative stress: implications for human health. *Antioxidants & redox signaling* 2013; **19**(2)**:** 192-208.

27. Belle MD, Piggins HD. Physiology. Circadian time redoxed. *Science* 2012; **337**(6096)**:** 805-806.
